# Intensification in pastoralist cereal use coincides with the expansion of trans-regional networks in the Eurasian Steppe

**DOI:** 10.1038/s41598-018-35758-w

**Published:** 2019-06-10

**Authors:** Alicia R. Ventresca Miller, Cheryl A. Makarewicz

**Affiliations:** 1 Graduate School Human Development in Landscapes, Christian-Albrechts Universität zu Kiel, Leibnizstrasse 3, 24118 Kiel, Germany; 2 Archaeological Stable Isotope Laboratory, Institute of Prehistoric and Protohistoric Archaeology, Christian-Albrechts Universität zu Kiel, Johanna Mestorf Strasse 2-6, 24118 Kiel, Germany; 3 Max Planck Institute for the Science of Human History, Department of Archaeology, Kahlaische Strasse 10, 07745 Jena, Germany

**Keywords:** Proteins, Stable isotope analysis

## Abstract

The pace of transmission of domesticated cereals, including millet from China as well as wheat and barley from southwest Asia, throughout the vast pastoralist landscapes of the Eurasian Steppe (ES) is unclear. The rich monumental record of the ES preserves abundant human remains that provide a temporally deep and spatially broad record of pastoralist dietary intake. Calibration of human δ^13^C and δ^15^N values against isotope ratios derived from co-occurring livestock distinguish pastoralist consumption of millet from the products of livestock and, in some regions, identify a considerable reliance by pastoralists on C_3_ crops. We suggest that the adoption of millet was initially sporadic and consumed at low intensities during the Bronze Age, with the low-level consumption of millet possibly taking place in the Minusinsk Basin perhaps as early as the late third millennium cal BC. Starting in the mid-second millennium cal BC, millet consumption intensified dramatically throughout the ES with the exception of both the Mongolian steppe where millet uptake was strongly delayed until the end of first millennium cal BC and the Trans-Urals where instead barley or wheat gained dietary prominence. The emergence of complex, trans-regional political networks likely facilitated the rapid transfer of cultivars across the steppe during the transition to the Iron Age.

## Introduction

**Figure 1:**
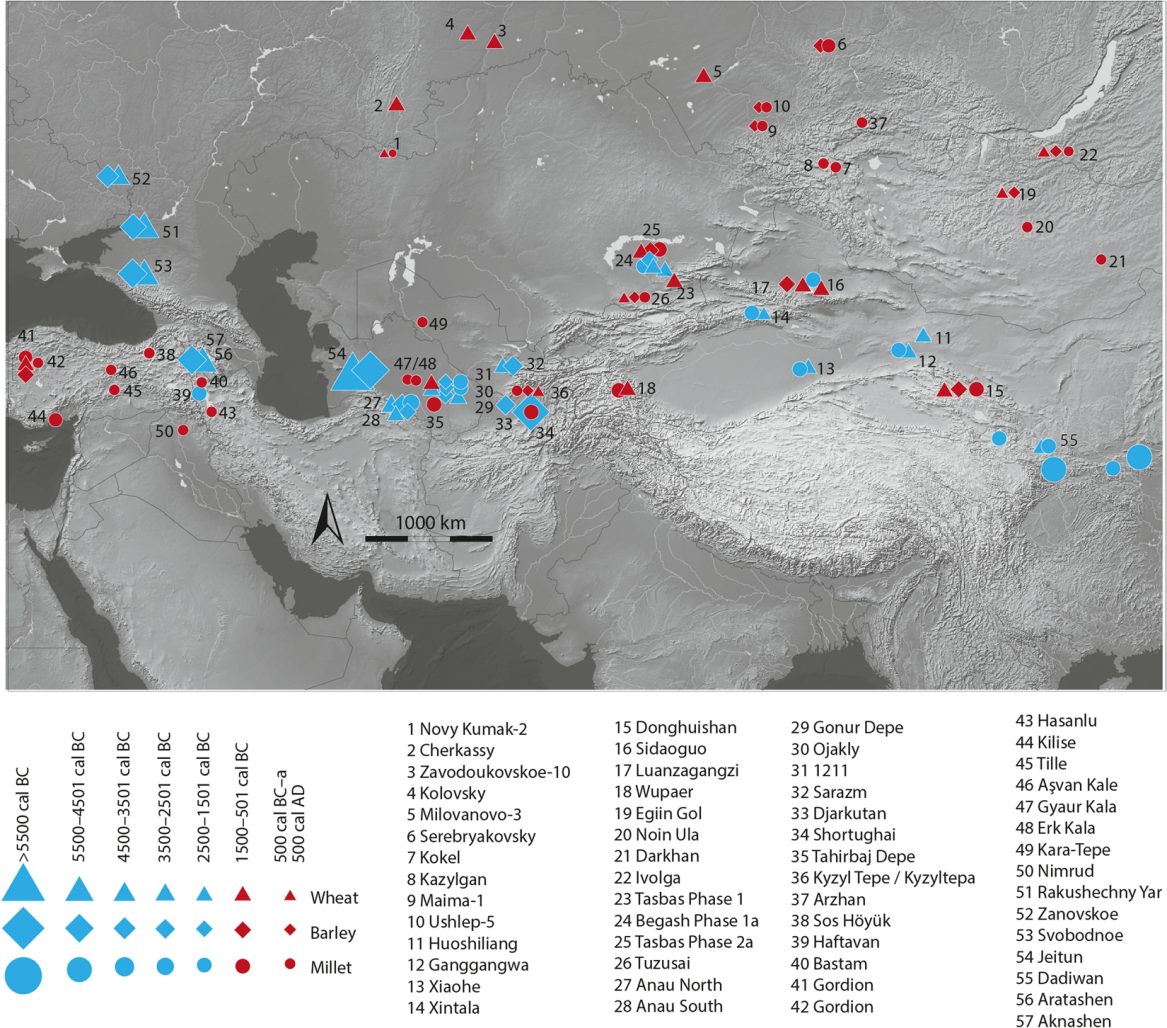
Distribution of millet, wheat and barley in the Eurasian steppe across time and space as identified in the carbonized seed record. Basemap constructed in ArcGIS 10 with hillshade generated using, http://www.naturalearthdata.com (freely available). Seed locations were generated based on published data using Adobe Illustrator CS.

The Eurasian Steppe (ES) is a vast cultural crossroads situated between two independent centers of crop domestication of southwest Asia and China and is a region key to understanding the spread of domesticated plants and animals (Fig. 1)^1–4^. Despite the expansive distances that characterize the steppe, the ES served for millennia as a dynamic socio-economic intersection through which commodities and technologies flowed via pastoralist communities who relied on domesticated livestock for subsistence. The transmission of cultivars across the ES was also facilitated by pastoralists who exchanged and, in some regions, cultivated cereals for subsistence and ritual use^3,5,6^. However, the pace of cultivar uptake and the intensity of cereal consumption by pastoralists remains poorly understood during two critical periods, from the third to second millennia cal BC when the translocation of wheat, barley, and millet grains into the ES first took place, and the mid-second through first millennium cal BC when inter-regional trade and exchange networks intensified and complex political structures emerged across the steppe^7–11^.

The paleobotanical record has provided important glimpses into the translocation of cultivars across the ES. After the initial domestication of wheat (*Triticum* sp.) and barley (*Hordeum sp*.) in southwest Asia by 8,500 cal BC, these cultivars spread into the Iranian Plateau by 6000 cal BC and into Pakistan by c. 5500 cal BC^12–15^. In southern Central Asia, domesticated forms of barley and wheat identified in Jeitun cultural contexts in Turkmenistan suggests these cultivars had spread to western Central Asia around 6100 cal BC^16,17^. Domesticated wheat and barley were cultivated in the southern Caucasus by c. 6000 cal BC^18,19^ and were present in the northern Caucasus by c. 4500 cal BC^20^. Wheat and barley spread into eastern Ukraine by 4000 cal BC and into the Crimean steppe by 3400 cal BC^19^. Notably, the carbonized seed record suggests there was a significant delay in the introduction of wheat and barley into the central ES and western China, which did not begin until the mid-third millennium cal BC^21–26^. The earliest evidence for the use of wheat and barley in the central ES is in the form of carbonized grains from southern Kazakhstan directly dated to c. 2500 cal BC^25^. In China, barley and wheat were present in Xinjiang by c. 2000 cal BC^23,26^ and in the Hexi corridor at 1700 cal BC^24^.

A considerable lag between initial millet domestication and its spread throughout the ES is also evident. Domesticated broomcorn millet (*Panicum miliaceum*) and foxtail millet (*Setaria italica*) seeds recovered from Neolithic agricultural settlements located in northeastern China dating to the early sixth millennium cal BC strongly suggests this region was a center for initial millet domestication^27,28^. Domesticated broomcorn and foxtail millet grains from Inner Mongolia directly dated to the mid-sixth millennium cal BC suggests that millet flowed relatively rapidly out of China to the northeast^29^. However, the Xinjiang region and ES remained devoid of broomcorn millet until the early second millennium cal BC^2,5,30^. Broomcorn millet was present on the western edges of the ES in southeastern Kazakhstan by 2200 cal BC, somewhat later than wheat^5^, and in Turkmenistan and Afghanistan by the mid-second millennium cal BC^3,6,31^. Overall, evidence for broomcorn millet in the ES at this time is sparse, limited to paleobotanical remains and impressions visible in ceramics from the Trans-Urals (late 2^nd^ to early 1^st^ mil. BC), Southern Siberia and the Altai (early 1^st^ mil. BC to 1^st^ cent. AD), and from Mongolia (late 1^st^ mil. BC)^3^ (Fig. 1). Recent direct dating of charred seed remains recovered from central and southeast Europe indicate that millet was present in the region by the mid second millennium cal BC^19^. In the Caucasus region, evidence for millet is limited to a single impression on pottery obtained from a broadly dated context dating to 6200–3300 cal BC^19^.

While the carbonized seed record has been instrumental in tracing the spread of millet, wheat, and barley throughout the ES, the temporal correspondence between initial use of cultivars and their incorporation into pastoralist diets remains under-defined, as does the degree to which the intensity of pastoralist cereal exploitation articulated with socio-political developments across the steppe. Dietary intake recorded in the isotope record of ancient human tissues have previously provided evidence for the spread of domesticated crops both westward across China^32^ and out of Mesoamerica into North America and South America^33,34^. We employ a similar approach to examine the spread of isotopically distinct C_4_ millet and C_3_ barley and wheat throughout the ES, but we further refine the approach by calibrating the distribution of human carbon and nitrogen isotopes against the isotopic composition of animals commonly exploited by pastoralists including domesticated sheep, goats, cattle and horses. This method takes into consideration the diverse environmental inputs that affect the isotopic composition of steppe foodwebs, in particular aridity and water availability which influences the natural abundance of C_3_ and C_4_ flora in steppic vegetational biomes and the carbon isotopic composition of C3 plants which underscore the isotopic composition of the animal-based portion of pastoralist diets.

**Figure 2:**
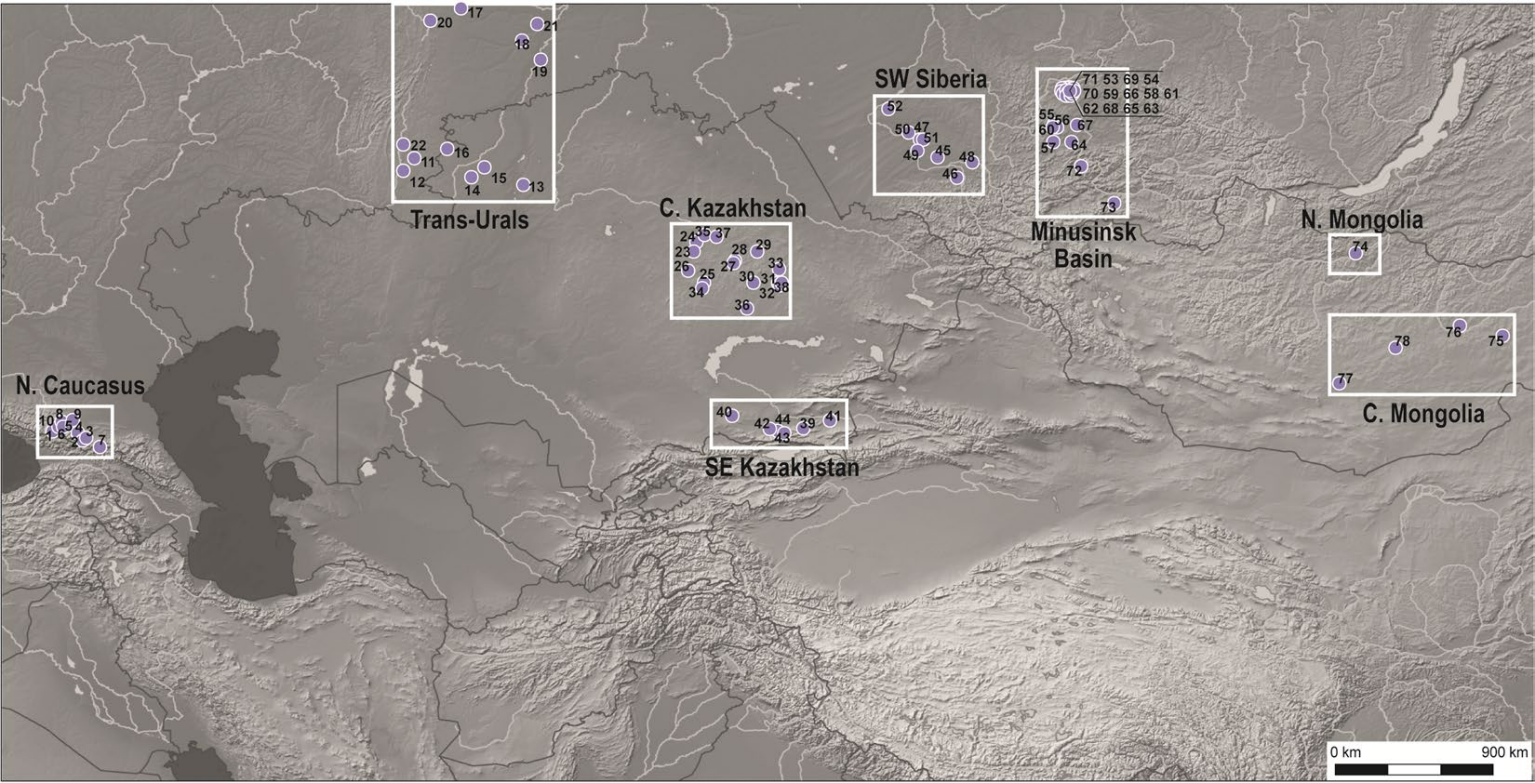
Locations of sites used in stable isotope meta-analysis: 1 Kabardinka, 2 Kudachurt, 3 Zaragizh, 4 Baksanyonok, 5 Gorjachevodskii 2, 6 Nezhinskaya, 7 Zamankul, 8 Zanozina Balka, 9 Inosemsevo, 10 Klin-Yar, 11 Kamennyi Ambar V, 12 Bolshekaragansky, 13 Bestamak, 14 Novoilinka, 15 Lisakovsk, 16 Isiney, 17 Kurtuguz I, 18 Murzino 1, 19 Skaty 1, 20 Shaidurikha, 21 Gayovsky I, 22 Pobedy, 23 Tengiszhol, 24 Temirkash, 25 Nurtaldy, 26 Dariya, 27 Tashik, 28 Aschisu, 29 Ayap-bergen, 30 Kopa-1, 31 Akimbek, 32 Tasyrbai 2, 33 Kyzylkol, 34 Kairakty, 35 Kudryavaya Sopka-1, 36 Kyzyl, 37 Karatugai, 38 Kent, 39 Kyzyl Bulak, 40 Oi-Dzailau VII, 41 Karatuma, 42 Kargaly 1, 43 Alatau 1, 44 Kamenka, 45 Itkul, 46 Ust-Isha, 47 Firsovo XI, 48 Solnts 5, 49 Tuzovskiye, 50 Teleutsky Vzvoz, 51 Firsovo XIV, 52 Chicha, 53 Afanasyeva Gora, 54 Karasuk III, 55 Uibat III, 56 Uibat V, 57 Verhniy Askiz I, 58 Bateni, 59 Melnichniy Log, 60 Okunev Ulus, 61 Solnechniy Log, 62 Upper Karasuk River, 63 Yarki, 64 Chernoye Ozero I, 65 Grishkin Log, 66 Lepeshki, 67 Nurilkov Ulus, 68 Podgornoye Ozero, 69 Saragash, 70 Saragshinskoe Ozero, 71 Saragashinskiy Spusk, 72 Ai-Dai, 73 Amyrlyg, 74 Egiin Gol, 75 Ulaanzuukh, 76 Daram uul, 77 Tevsh, 78 Baga Gazaryn Chuluu. Basemap constructed in ArcGIS 10 with hillshade generated using, http://www.naturalearthdata.com (freely available). Site locations generated in Adobe Illustrator CS6.

We report here a comprehensive meta-analysis of previously published carbon and nitrogen isotope values from humans and co-occurring livestock from Bronze Age and Iron Age sites located in the ES dating to ca 5000 cal BC to 400 AD (Fig. 2)^11,35–58^. The precise chronology of each cultural period varies across the ES reflecting regional differences in the pace of local social, economic, and political developments as well as the robustness of local radiocarbon chronologies. Here, we use previously established cultural-chronological sequences constructed from combined cultural materials and radiocarbon analyses (Table S1); no further modeling of radiocarbon determinations were undertaken due to the lack of information available regarding the type of material analyzed (e.g., wood charcoal, carbonized seeds, or human or animal bone collagen) and, in the case of human remains, the potential impact of freshwater reservoir effects on dates continue to be unresolved for the steppe (see discussion in methods). Carbon and nitrogen isotopic data sets are derived from the collagenous fraction of human skeletal remains from socially distinguished individuals interred in monumental mortuary structures that were a ubiquitous feature of ancient ES landscapes and considered to be those of steppe pastoralists^8,11,35^. These mortuary features maintained regionally distinctive styles and functions as commemorative spaces^59^, social integrative elements^60,61^, and focal points for political action^11,62^.

## Significant Differences in Linear Regression Slopes Between Humans and Livestock Indicate Pastoralist Cereal Consumption

**Figure 3:**
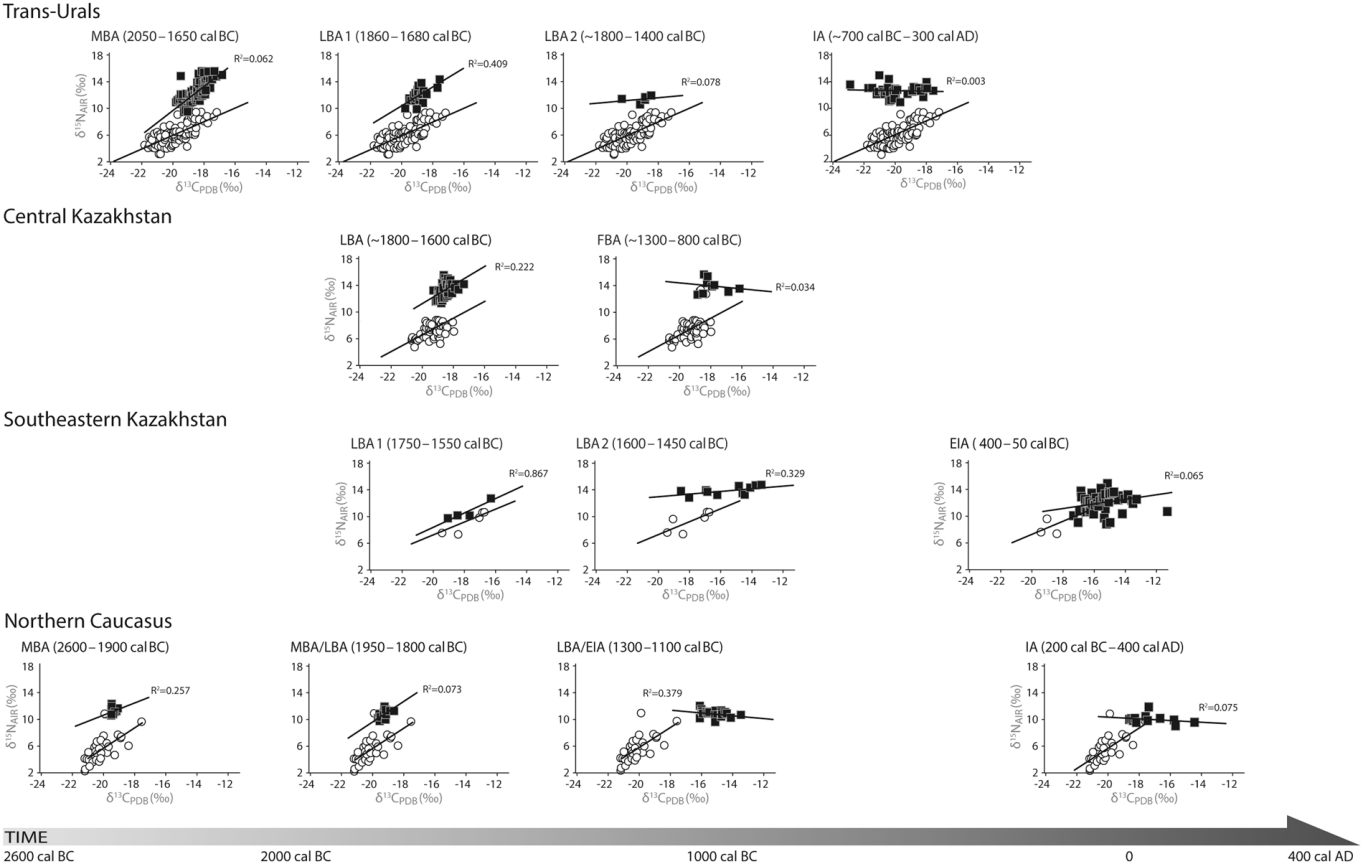
Carbon (δ^13^C) and nitrogen (δ^15^N) isotope values of humans (black squares) and livestock (open circles) for the Trans-Urals, Central Kazakhstan, Southeastern Kazakhstan and the Northern Caucasus.

**Figure 4:**
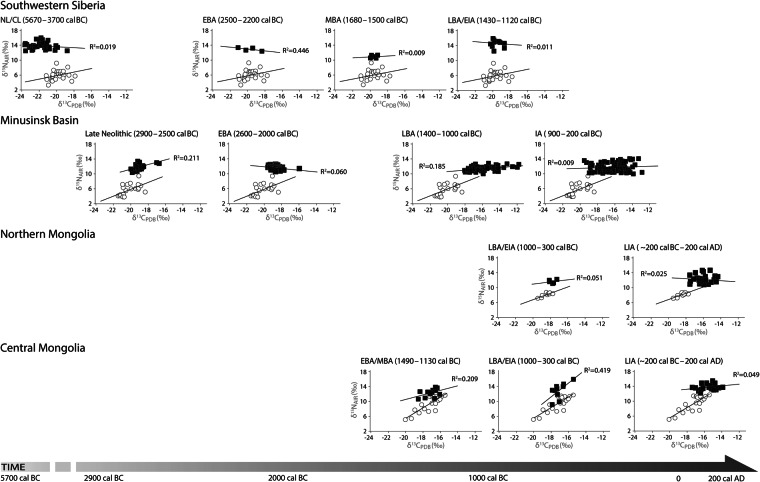
Carbon (δ^13^C) and nitrogen (δ^15^N) isotope values of humans (black squares) and livestock (open circles) for Southwestern Siberia, the Minusinsk Basin, Northern Mongolia and Central Mongolia.

Pronounced changes in the carbon and nitrogen isotopic composition of humans relative to co-occurring livestock indicate that a significant human dietary transition was underway by the mid-second millennium cal BC. Across the ES, Bronze Age human groups share carbon isospace with regionally corresponding livestock and exhibit a positive correlation between nitrogen and carbon isotope values, a pattern also observed in livestock (Figs 3 and 4). Bronze Age humans and livestock exhibit similar regression line slopes across the ES with the exception of paired groups from the Trans-Urals and Minusinsk Basin (Table S2). Isotopic congruence between humans and livestock indicate that the Bronze Age human dietome was primarily structured by the isotopic composition of steppe and forest steppe flora ingested by grazing livestock and, after accounting for trophic level fractionation effects, strongly suggests that Bronze Age pastoralist dietary intake in most regions relied heavily on animal products.

The shift in pastoralist food intake from diets based on the products of livestock including meat and milk as well as fish in some regions to diets that included an appreciable amount of cereals occurred at various timings and intensities across the ES. This dietary transition was likely underway throughout the mid-third and early second millennium cal BC in some regions, followed by relatively rapid uptake throughout the ES of cereal inclusive diets during the Iron Age starting in the mid-second millennium cal BC. This major dietary switchover to dedicated cereal consumption is evidenced in the isotopic record by (i) a continued positive correlation between δ^13^C and δ^15^N values exhibited in livestock but a breakdown in this isotopic relationship observed in contemporaneous humans indicating a dramatic departure from regional steppe dietomes (Figs 3 and 4), (ii) a significant shift in average δ^13^C values of Iron Age humans relative to Bronze Age humans (Table S3), and (iii) wide isotopic spacing between the carbon isotopes of Iron Age humans and reference livestock in most regions (Fig. S1). The contribution of wheat and barley to pastoralist diets that may also include millet remain invisible using this approach. We also calculated isotopic niches for each time period and region, to identify shifts in niche size and overlap, in an effort to clarify the introduction of new dietary inputs. An increase in isotopic niche size over time, as seen in density plots (S6), indicate an expansion of dietary diversity, in most regions, to include a ^13^C-enriched food source. The maximum percent overlap of Bayesian standard ellipse areas were plotted at a 99% confidence interval (CI) to highlight chronological variation in isotopic niches. Regionally, there was a decrease in the overlap of isotopic niches over time further supporting the introduction of a new food source in later periods.

## Regional Variation in the Intensification in Cereal Consumption

### Southwestern Siberia

Pastoralists in southwest Siberia appear to have consumed both quantities of fish and terrestrial animal protein in the Late Bronze and Early Iron Ages (LBA/EIA) during the mid to late second millennium cal BC indicated by high human bone collagen δ^15^N values averaging 15‰ and a c. 9‰ enrichment in human δ^15^N values compared to livestock^38^ (Figs 4, S1; Table S2). Bayesian ellipses and isotopic niches demonstrate a decrease of the isospace occupied by humans over time (Figs S2, S6.1, S6.2), suggesting that hunter-gatherer diets were more diverse than later pastoralist diets. The stable isotopic data do not confirm or reject the low-level consumption of cereals in later periods due to the high contribution of fish to diets in SW Siberia. Flotation at the LBA/EIA site confirms the presence of plant remains, but an absence of grains from domesticated cultivars, indicating that cereal crops did not figure into pastoralist diets in SW Siberia^38^.

### Minusinsk Basin

Low-level millet consumption by hunter-gatherer populations in the ES may have taken place in the Early Bronze Age (EBA) sometime during the late third to early second millennium cal BC suggested by i) the combination of a 1.6‰ increase on average in δ^13^C values (Table S3), ii) low δ^13^C values (c. −24‰) exhibited by Minusinsk Basin fish^49^, and iii) the breakdown of the positive linear relationship between carbon and nitrogen isotopes in EBA human collagens and livestock caused by the incorporation a ^13^C-enriched food source into the diet (Fig. 4; Table S2) (c.f.^49^). The absence of ^13^C-enriched fish in local waterways or indigenous C_4_ flora, indicated by low livestock δ^13^C values averaging 20‰, may indicate that low-level millet consumption is responsible for the isotopic patterns visible in EBA humans. We recognize that freshwater fish may have varied in isotopic composition and that fish could have been enriched in ^13^C relative to modern species. It may be that the consumption of fish, rather than millet, is responsible for the carbon isotopic pattern observed for this region and time period. Furthermore, in the absence of radiocarbon determinations from human individuals analyzed for carbon and nitrogen isotopes, some individuals in the EBA group could date to as late as c. 2000 cal BC. Millet was clearly an important part of pastoralist diets by the mid-second millennium cal BC in the Minusinsk Basin. This is indicated by the pronounced 2.7‰ increase, on average, in human δ^13^C values from the EBA to LBA (Table S3) and a shift in the carbon isospace occupied by LBA humans (EBA = −19.5‰ to −15.9‰; LBA = −18‰ to −11.8‰) confirming previous findings for the region^49^. High levels of millet consumption were maintained through the Iron Age, while fish regained dietary importance. Differences in the isospace occupied by temporally varied human groups were compared by plotting standard ellipse areas (SEA; Fig. S2) and as Bayesian permutations of standard ellipse areas (SEA_B;_ Fig. S6). The maximum percent overlap of SEA_B_’s of LN and EBA groups has a 99% probability of being below 60% (S6) while the EBA ellipse slope varies from the earlier period. The overlap visible in the EBA ellipse with the ellipses of ^13^C enriched LBA and IA humans (Figs S2 and S6) further suggests that dietary intake differed between these periods and that humans incorporated low levels of a ^13^C-enriched food source, such as millet, into the EBA human diet. A density plot demonstrates the confidence intervals of the different ellipse areas, demonstrating the expansion of isotopic niches over time. Isotopic niche sizes varied notably between the early (LN, EBA) and later (LBA, IA) groups, increasing in the later periods (S6.3-S6.5). The LBA and IA niches expanded greatly compared to earlier time slices, indicating the consumption of a ^13^C-enriched food source, which lends further support for the millet consumption in these periods.

### Southeastern Kazakhstan

No isotopic evidence for cereal consumption is visible in the remains of pastoralists inhabiting SE Kazakhstan in the early second millennium, although sample sizes are small (Fig. 3, LBA1) (Sensu^55^). By the mid-second millennium cal BC, the combination of a 2.3‰ increase on average in LBA2 human carbon isotope values and loss of a positive linear relationship between carbon and nitrogen isotope values in humans and livestock indicates that millet was incorporated into pastoralist diets at this time. The overlap in dating for the two LBA time slices suggests that communities had varied dietary intake in this region. Moderately high human δ^13^C values averaging 15.4‰ and a breakdown in the positive correlation between regression slopes of EIA humans and livestock (Fig. 3) indicates moderate levels of millet consumption continued through the end of first millennium cal BC. These findings are supported by Bayesian ellipses of humans (Fig. S2 and S6.6–S6.8) which demonstrate incomplete overlap (<40%) between LBA1 and LBA2. The separation of isotopic niches between LBA1 and LBA2 suggests dietary distinctions between pastoralist groups, while the overlap between ellipses for LBA2 and ^13^C enriched EIA groups suggests new dietary inputs, such as millet, for both groups.

### Central Kazakhstan

Low-level millet consumption is evident in pastoralist diets in Central Kazakhstan during the late second millennium cal BC at the tail end of the Bronze Age (Figs 2 and 3). Late Bronze Age (LBA) humans and livestock exhibit similar positive regression line slopes, while later Final Bronze Age (FBA) humans and livestock exhibit significantly different regression slopes (Table S2). This, in addition to the significant 0.6‰ enrichment in FBA human δ^13^C values relative to LBA humans, and moderate increase in the carbon isotopic spacing between humans and livestock during the FBA (Figs 3, S1; Tables S2, S3), suggest the addition to FBA pastoralist diets of a C_4_ food source outside of that typically available in this steppe environment. High δ^15^N values of humans, averaging 13.5‰, were maintained throughout the Bronze Age reflecting considerable animal protein intake. The maximum percent overlap of SEA_B_’s (LBA and FBA groups) have a 99% probability of being below 60% (S6) suggesting a change in human dietary intake over time. The expansion of the isotopic niche for FBA humans indicates that dietary intake included new dietary inputs, likely low levels of a ^13^C-enriched food source such as millet (Figs S6.9–S6.11).

### Mongolia

Millet appears to have circumvented the Mongolian steppe throughout much of the Iron Age. In N. Mongolia, the combination of a significant difference in regression line slopes computed for LIA humans and herbivores (Table S2), a significant 1.7‰ enrichment in the δ^13^C values of LIA humans relative to LBA/EIA humans (Table S3), and a wider 2.4‰ carbon isotopic spacing between humans and livestock (Fig. S1) together indicates that the incorporation of millet into pastoralist diets did not take place until the end of the first millennium cal BC. The absence of nitrogen isotopic change between LBA/EIA and LIA humans suggests little change in animal protein intake over time (Table S3). These findings are further supported by Bayesian standard ellipses that show the LBA/EIA and LIA overlapped less than 40%. Furthermore, the LIA ellipse demonstrates a greatly expanded niche, indicating a significant dietary change in this period that included the consumption of millet (Figs S2, S6.12–S6.14).

Millet appears to have been consumed in central Mongolia during the Late Iron Age. In contrast to similar regression line slopes calculated for Bronze Age humans, LIA humans yield a different carbon and nitrogen isotopic relationship. Regression line slopes of LIA humans were significantly different than those of earlier humans as well as livestock (Fig. 4, Table S2). The moderate, albeit significant, 1.6‰ enrichment in LIA human δ^13^C values relative to MBA humans, and moderate 1.5‰ carbon isotope spacing between LIA humans and livestock suggests low level incorporation of millet into the diets of pastoralists inhabiting the northern Gobi. The increase in the nitrogen isotopic spacing between humans and livestock over time, from 2.8 to 4.3‰ in the water poor Gobi steppe desert, likely reflects a shift in animal management strategies to include grazing of livestock on ^15^N enriched winter pastures during the LIA^53^. Bayesian standard ellipses (99%) indicate considerable overlap between the EBA/MBA and LBA/EIA (70%), demonstrating only slight variation in dietary intake. A decrease in the overlap between LBA/EIA and LIA ellipses (<45%) coincide with an expansion of the LIA ellipse toward higher carbon isotope values suggesting that dietary intake shifted in the later period to include a ^13^C-enriched food source such as millet (Figs S2, S6.15–S6.17).

### Trans-Urals

Millet contributed little, if at all, to pastoralist diets in the Trans-Urals. Instead, C_3_ cultivars such as wheat and barley appear to have been gradually incorporated into pastoralist diets during the mid-second millennium cal BC and became a dietary focus by the mid-first millennium cal BC during the Iron Age. During the early second millennium cal BC, the combination of high δ^15^N values averaging 12.3‰ and significant differences between positive regression slopes visible in human and livestock isospace indicates fish were an important part of the MBA human diet (Fig. 3). During the subsequent LBA 1 and LBA 2, which are contemporaneous periods, variation in dietary strategies emerges. During the LBA 1, at the site of Lisakovsk, the reduction in the range of nitrogen isotopes weighted toward lower δ^15^N values in humans combined with similar regression slopes exhibited by humans and livestock suggest that fish consumption decreased considerably by the early second millennium cal BC and was at least partially replaced by animal products derived from livestock. Pastoralist dietary intake appears to have shifted at the LBA 2 site of Isiney indicated by a 0.6‰ decrease in human δ^15^N values from the MBA, suggestive of a further reduction in fish intake. Significant differences between human and livestock regression slopes indicate Trans-Urals pastoralist diets included some cereals by the mid second-millennium cal BC (LBA 2, Fig. 3; Table S2). C_3_ cereals became an important part of pastoralist diets by the mid-first millennium cal BC during the Iron Age (IA) indicated by (i) the significant 0.8‰ depletion in human δ^13^C values relative to the preceding LBA 2 (Table S3), (ii) an expanded range of IA carbon isotopes heavily weighed to lower δ^13^C values between −17.5‰ to −22.8‰ (compared to all earlier periods which exhibit a restricted range of δ^13^C values from c. −16.8‰ to −20.3‰) (Tables S2, S3), and (iii) the absence of a positive linear relationship between carbon and nitrogen isotopes expressed in human collagens (Fig. 3). These findings are supported by differences between Bayesian standard ellipse areas. Overlap between the MBA and LBA 1 ellipses are high, reaching 85% (Figs S2, S6.18–S6.20) indicating similar dietary niches. Ellipses for LBA 1 (Lisakovsk) and LBA 2 (Isiney) have less of an overlap (60%), and the latter has an ellipse with an expanding niche width, suggesting a new dietary input (S6.19, S6.20). Dietary change is clear by the IA, where the isotopic niche has expanded to include a ^13^C-depleted food source, such as domesticated wheat or barley.

### The Northern Caucasus

The incorporation of millet into pastoralist diets took place in the Early Iron Age (EIA) during the late second millennium cal BC in the N. Caucasus, indicated by similar positive regression slopes exhibited by Bronze Age humans and livestock followed by a significant difference in this relationship during the Iron Age (Fig. 3). In addition, MBA and MBA/LBA humans exhibited only a 0.6‰ spacing between human and livestock δ^13^C values, reflecting pastoralist dietary intake driven by carbon isotopic variation in local pastures. A subsequent 5.0‰ and 2.7‰ spacing between LBA/EIA and IA humans and livestock, respectively, is consistent with the pronounced contribution of a C_4_ food source to human diets (Fig. S1). The significant c. 0.7‰ enrichment in ^15^N and depletion in ^13^C of IA humans relative to preceding LBA/EIA humans may suggest dietary diversification in the later period and the initial contribution of a C_3_ food source such as wheat or barley (Tables S2; Fig. S1). Isotopic niche widths were plotted as standard ellipse areas (SEA; Fig. S2) and as Bayesian permutations of standard ellipse areas (SEA_B;_ Fig. S6.21). SEAs demonstrate strongly overlapping ellipses for MBA and MBA/LBA groups, with low overlap between these groups and the IA (Fig. S2). There is also low overlap between the LBA/EIA and the IA suggesting significant variation in dietary intake between these periods. Plots of SEA_B_ for humans and animals through time further demonstrate differences between early (MBA, MBA/LBA) and later (LBA/EIA, IA) populations in the region (Fig. S6.21, S6.22). The expansion of isotopic niches in the later periods indicates that dietary intake differed between periods and that humans incorporated a ^13^C-enriched food source, such as millet, into their diets.

### Temporal disparity between first evidence of cereals, subsequent incorporation into pastoralist diets, and intensification of cereal consumption in the ES

A substantial lag time between initial translocation and intensive consumption of cultivars is evidenced in both the carbonized seed record and the stable isotopic record in several regions of the ES (Figs 1; S2, S3, S4). The earliest millet was present in SE Kazakhstan by 2200 cal BC^5^ but, as indicated in the human isotopic record, did not have a significant dietary impact until c. 1500 cal BC. Similarly, in the Trans-Urals indirectly dated wheat grains suggests the cultivar was present c. 1750 cal BC but was not an important part of the pastoralist diet until c. 1600 cal BC. This disparity in uptake could indicate the conversion of cereals from an exotic cultigen used in ceremonial contexts during the Bronze Age, as seen at the pastoralist settlement of Begash (where millet grains recovered from human burial cists points toward the ritual use of cereals in mortuary contexts)^5^, to a subsistence food that was consumed at varied intensities by different pastoral populations across the ES.

### Participation in trans-regional political exchange networks redirected pastoralist dietary intake

The marked intensification in the consumption of grains by pastoralists coincided with a shift in the configuration of socio-political relationships from internally-focused, community oriented interaction spheres, to externally oriented networks that facilitated the creation and maintenance of trans-regional political relationships across the ES. During the third and second millennia BC, Bronze Age interaction spheres facilitated the flow of locally available prestige goods and operated at regional scales^8,11,63,64^. Burial practices emphasized the collective, perhaps kin-based, interment of multiple individuals from the same community^8,65^ and monument construction facilitated local community cohesion and integration^11,66^. The emergence of trans-regional connections during the first millennium cal BC culminated in the formation of socio-political confederations in multiple regions^7–11,67^. This process was evidenced by a marked increase in the exchange and use of costly prestige goods sourced from diverse locales across Eurasia and China^11,68^, the construction of ostentatious mortuary monuments memorializing individuals, and the spread of horse-riding paraphernalia^7,35,69^. Furthermore, as horseback riding became the norm, based on the regular apperance of bits and bridles in mortuary contexts^35,70,71^, individuals and small groups were able to move at the speed of the horse rather than the herd, increasing the frequency and regularity of contact between communities as well as regions, facilitating the growth of networks, and amplifying the flows of goods and technologies. While Iron Age groups have generally been cast as highly nomadic pastoralists, it remains to be seen if they engaged in intensive agricultural production. Our findings indicate that it is precisely during this period of proposed nomadism when the consumption of cereals intensifies across Eurasia.

Considerable regional variation in the intensity of millet consumption by pastoralists across the ES is likely due to differences in the intensity of local cultivation, degree of local participation in trans-regional networks, and importance of access to cultivars in structuring socio-political relationships. In the case of the Minusinsk Basin during the late third millennium cal BC, possible low-level millet consumption (Fig. S3) coincided with the intensification of trans-regional interactions with communities associated with the Chemurchek (Qiemu’erqieke) tradition located across the Russian Altai, western Mongolia, and NW Xinjiang^72^, where similar flat bottomed jars, stone cist burials, and anthropomorphic stone stelae were recovered^73–75^. However, the carbonized seed record indicates millet was not used in Xinjiang until the early second millennium cal BC^23,76,77^ while high carbon isotope values associated with intensive millet consumption is not evident in the region until 900 cal BC^78,79^. The combined archaeological and isotopic data from the Minusinsk Basin may indicate millet was cultivated at an earlier date in Xinjiang or alternatively, albeit less likely given the absence of millet in the Baikal region, was introduced into the Minusinsk from sources in northeast China, where charred millet grains are present at c. 6000 cal BC^28^. Bronze daggers and socketed axes associated with the Andronovo or Karasuk cultures that are typical for the Minusinsk Basin were present in northeast China and indicate interactions between these regions during the late second millennium cal BC^80–83^.

The first isotopic evidence for low-level wheat or barley consumption occurs in the Trans-Urals and is visible at the beginning of the second millennium cal BC (LBA 2, Figs 3; S3). The chronological overlap between LBA 1 and LBA 2, but variation in dietary intake between humans assigned to each group suggests that Andronovo communities had diverse subsistence strategies. The initial influx of grains may have come from the southwest (Caucasus) region or from southern Central Asia, with the latter more likely as limited numbers of exotic items found in Andronovo sites are often connected to southern regions^8,82,84^. Intensification of wheat and barley consumption in the Trans-Urals occurred during the Iron Age when Sauro-Sarmatian and Sargat interaction spheres spread across large swaths of the Eurasian steppe, evident in shared prestige good assemblages, warrior equipment, and mortuary rituals^35^. This region was deeply involved in exchange with areas in central and southeastern Kazakhstan that consumed millet, yet Trans-Ural groups opted instead to grow wheat and/or barley. Early evidence for a dietary focus on wheat and/or barley, rather than millet, suggests that Trans-Urals populations may have been trading these cultivars to other areas of the steppe that lacked the water resources to cultivate these crops. Investments in different farming technique or a higher value placed on wheat and/or barley may also have been factors affecting the decision to focus on the production of these grains.

In SE Kazakhstan, diversification of pastoralism to include dedicated cultivation appears to have taken place at c. 1500 cal BC, indicated by cropping of millet, wheat, barley and peas and the local processing of harvested crops^1^ which roughly coincides with increased millet consumption seen in the stable isotopic record (Figs 3; S4). Early evidence for millet in SE Kazakhstan may have been introduced from western China, while wheat and barley were introduced from southern Central Asia, as interactions along the mountain corridor were longstanding^85^. During the second millennium cal BC, durable networks of interaction linked western China and southern Central Asia through SE Kazakhstan^1,85^.

Low–level consumption of millet in Central Kazakhstan, which occured several hundred years later, is associated with the emergence of regional centers and participation in pan-regional interaction spheres, from the Urals to the Altai, which may have facilitated access to cultivars^8,86^. The incorporation of millet into pastoralist diets, albeit at low-levels, in C. Kazakhstan by c. 1050 cal BC also coincided with a shift in socio-political relationships (Fig. S4). Late Bronze Age interactions were locally oriented, evidenced by a lack of imported goods and diverse mortuary rituals, while participation in wider trans-regional networks occurred during the FBA, specifically with areas of southeastern Kazakhstan where similar ceramic ornamentation and bronze ornamental goods have been identified^8,25,86,87^.

The intensification in millet consumption in the northern Caucasus by c. 1200 cal BC (Fig. S4) coincided with the emergence of an extensive exchange system centered in the Caucasus that facilitated movement of raw materials required for tin-bronze metal working and finished metal goods^88^. While millet has been identified in the southern Caucasus as early as c. 1600 cal BC, the introduction of this crop to mountainous zones only occurred as network interactions became externally focused. This only occurred after 1200 cal BC when imported goods such as Assyrian helmets were recovered from local burial contexts^89^ and locally made weapons and armor were exported as prestige goods^7^.

In the Mongolian steppe, millet consumption levels were the lowest in the ES for the Iron Age (Fig. S5) and suggests either local cultivation activities were small in scale or the Xiongnu elite engaged in indigenous forms of ideological expression centered on domesticated livestock and steppe nomadism^11,68^, or a combination of both. The late incorporation of millet into pastoralist diets in the Mongolian steppe coincided with macro-regional integrations of the Xiongnu political confederation at the end of the first millennium cal BC^10,11^. Notably, although millet was an important part of Iron Age steppic communities, nowhere in the ES did pastoralist millet intake approach the intensive levels of millet consumption seen in contemporaneous agricultural societies in China, where high δ^13^C values observed in human collagens ranging from −6.5 to −11‰ are common^90–92^.

## Discussion

The transformation of pastoralist dietary intake from a heavy reliance on livestock products, or hunting-fishing-gathering in some regions, during the Bronze Age to consumption of cereals during the transition to the Iron Age took place alongside a radical change in the scale of pastoralist interaction from inward focused interaction spheres to trans-regional, externally-oriented networks during the mid-second millennium BC. The apparently substantial temporal lag between the first appearance of cereals visible in the carbonized seed record and the regular inclusion of cereals into human diets indicated in the bone collagen isotopic record likely reflects the conversion of cereals from an exotic and rare cultigen, used in Bronze Age ritual contexts, to a subsistence food possibly used to demarcate elite status in pastoralist societies participating in increasingly interconnected trans-regional political networks. The first evidence for a dietary transition, and possibly millet consumption, in the ES appears to occur in the Minusinsk Basin which would suggest a route of grain translocation through western China (Xinjiang) or, less likely, across southern Russia originating in far northeastern China (Fig. S2). As millet next appears in SE Kazakhstan, the route through Xinjiang seems more likely (Fig. S4). Millet was then slowly incorporated into pastoral diets in central Kazakhstan and the N. Caucasus, while groups in the Trans-Urals region either consumed millet in very small amounts or opted to focus on wheat and barley (Figs S2–S4). The relatively minor contribution of millet to the diets of pastoralists inhabiting the Mongolian steppe throughout the first millennium AD (Figs S3, S4) suggests that populations maintained pastoralist lifeways connected to indigenous ideologies centered on human-animal relationships.

## Methods

Previously published human and livestock δ^13^C and δ^15^N values obtained from bone collagen were grouped according to broad environmental zones in order to account for variation in the isotopic composition of locally available dietary resources. Published human isotopic data sets that did not include paired livestock values were not included in this study. The ES is broadly divided into two environmental zones, the forest-steppe and grassland steppe which support C_3_ vegetation and mixed C_3_/C_4_ vegetation, respectively^93–95^. The overall carbon isotope composition of steppe and forest-steppe floral biomes varies according to regional precipitation levels, temperature, aridity, and topography which together influence the relative abundance of ^13^C depleted C_3_ and ^13^C enriched C_4_ flora^96^ and modify the carbon isotope composition of C_3_ plants. Here, the ES is divided into 8 zones according to location of sampled sites and regional differences in phytogeographic and climatic conditions that broadly determine the carbon isotope composition of regional floral biomes (Fig. 2). These zones include the (1) C_3_ mountainous uplands of the northern Caucasus, (2) largely C_3_ foothills and steppe of the Trans-Urals, (3) C_3_/C_4_ steppe of central Kazakhstan, (4) the C_3_/C_4_ steppe of southern Kazakhstan, (5) mainly C_3_ Baraba forest-steppe of SW Siberia, (6) largely C_3_ steppe/forest-steppe of the Minusinsk Basin, surrounded by high, densely forested mountains, (7) primarily C_3_ forest-steppe of northern Mongolia, and (8) C_3_/C_4_ Gobi steppe desert of south central Mongolia.

The broad differences in vegetation composition, aridity levels and topography characterizing each of these eight zones together influence the carbon and nitrogen isotope composition of the floral base of the foodweb. This is further reflected in the isotope composition of herbivorous consumers, in this case grazing and browsing livestock^97–99^. In order to distinguish the contribution of ^13^C enriched or ^13^C depleted agricultural cereals to pastoralist diets from foodstuffs that derive from steppe food resource pools, which include milk and meat products obtained from livestock grazing on ^13^C enriched C_4_ and water-stressed C_3_ graze, we used independent isotope reference sets that measure isotope variation in local foodwebs using δ^13^C and δ^15^N values of livestock that are spatially and temporally congruent to the human sample set. The δ^13^C and δ^15^N values of pastoralists whose dietary intake draws heavily from animal products should closely track those of domesticated herbivores alongside a trophic fractionation factor of + 3–6‰ in δ^15^N^100–102^ and +1‰ in δ^13^C^103,104^, represented by positive regression lines similar to those exhibited by co-occurring livestock ingesting local floral resources. Conversely, pastoralist diets characterized by a sustained consumption of ^13^C enriched millet or ^13^C depleted barley and wheat no longer track herbivores and instead exhibit significantly different regression lines from co-occurring livestock indicating consumption of dietary resources typically not available on steppe landscapes.

Average δ^13^C and δ^15^N values of humans grouped by region and chronological period were compared using an analysis of variance coupled with Tukey’s test^105^ to determine the statistical significance of differences between human diets across time and space (Table S3). T-tests of the slopes of regression lines, which indicate a relationship between two variables (δ^13^C, δ^15^N), were used to compare humans to livestock by region (Table S2). Inter-regional comparisons of the intensity of pastoralist cereal consumption were carried out by standardizing regional human collagen δ^13^C values against the δ^13^C values obtained from co-localized livestock (Fig. S1); this approach removes regionally specific environmental isotopic overprints that are incorporated into human tissue via ingestion of animal products. Similar standardization was carried out for human collagen δ^15^N values against those of regional livestock to evaluate variation in the nitrogen isotopic composition of human diets over time (Fig. S1). Standardization, or the magnitude of isotopic spacing, was calculated between herbivore and human values (∆_herbivore − human_) for each time period (Fig. S1).

Time slices within each region were established based on accepted chronological brackets defined by material culture types and stylistic forms associated with radiocarbon dates. The construction of robust radiocarbon chronologies in the Eurasian steppe is so far constrained as there are relatively few radiocarbon determinations available for this broad region. For example, recent Bayesian modeling of radiocarbon dates from Bronze Age and Early Iron Age^106^ Mongolia encompasses 1500 years of prehistory, yet is based on a sample of only 45 dates. This is one of the most robust chronological datasets currently available for Eurasia^106^ and similar in size to a dataset (n = 40) analyzed a decade ago for the Trans-Urals region^39^. Reported radiocarbon determinations are also frequently under-described as to the material dated which may have included wood charcoal, carbonized seeds, as well as human and faunal bone, substrates that may yield incongruent radiocarbon determinations depending on the biological history of the dated sample. Many of the sites in the Eurasian steppe are located alongside rivers and lakes, and human diets may have included freshwater resources which potentially result in anomalously old radiocarbon dates. The impact of the freshwater reservoir effect (FRE) on radiocarbon dates derived from human bone collagen in the study region has been extensively studied^41,107–109^ with results indicating substantial variation in FRE neccesitating assessment of radiocarbon dates on a case by case basis^108,110^. Resolving issues of small sample sizes, poor reporting of dated materials, and a full consideration of modern and archaeological isotope values to deal with the reservoir effect are well beyond the scope of this paper. Therefore, we used established radiocarbon date ranges from scholars that are specialists in their region and time period to determine date ranges for each time slice.

We chose to retain outliers present in stable isotopic datasets as they convey meaningful information regarding intra-community variation. In order to overcome issues with small sample sizes we employed SIBER (Stable Isotope Bayesian Ellipses in R) to construct Bayesian ellipse standard areas (SEA_B_) at a 99% confidence interval (CI) for each time period. In a Bayesian approach, a set of iterative draws (10^10^) from a simulation is used to construct an ellipse and derive metrics, such as the area, which is referred to as SEA_B_. This process is repeated for all simulated values, producing a range of probable values for the calculated metric. Bayesian ellipses take into account uncertainty in the sampled data and incorporate error arising from the sampling process. Ellipses are unbiased with respect to sample size and robust comparisons can be made between data sets^111^. We also used density plots by region that indicate the confidence intervals of standard ellipse areas for each human group respectively. Further, we computed the percent overlap for ellipses between periods when dietary shifts were occurring. These were computed at 99% confidence and represent the maximum amount of overlap of two Bayesian standard ellipse areas (Fig. S6).

## Electronic supplementary material

Figure S1, Figure S2, Figure S3, Figure S4, Figure S5, Figure S6, Table S1, Table S2, Table S3, Table S4, Table S5
