# Author Correction: Intensification in pastoralist cereal use coincides with the expansion of trans-regional networks in the Eurasian Steppe

**DOI:** 10.1038/s41598-022-19230-4

**Published:** 2022-09-01

**Authors:** Alicia R. Ventresca Miller, Cheryl A. Makarewicz

**Affiliations:** 1 Graduate School Human Development in Landscapes, Christian-Albrechts Universität zu Kiel, Leibnizstrasse 3, 24118 Kiel, Germany; 2 Archaeological Stable Isotope Laboratory, Institute of Prehistoric and Protohistoric Archaeology, Christian-Albrechts Universität zu Kiel, Johanna Mestorf Strasse 2-6, 24118 Kiel, Germany; 3 Department of Archaeology, Max Planck Institute for the Science of Human History, Kahlaische Strasse 10, 07745 Jena, Germany

Correction to: *Scientific Reports* 10.1038/s41598-018-35758-w, published online 10 June 2019

The Article contains an error.

Data points from the Minusinsk Basin – Okunevo period (− 17.7, 10.9 and − 15.9, 11.5), used for Figure 4 in this Article, were based on the results from Svyatko et al. in which carbon and nitrogen isotopic data were assigned to a cultural-chronology based on relative dating. In subsequent work from these authors^2–4^, skeletal remains from which isotope values had been measured were radiocarbon dated. This confirmed that two of the individuals (out of 21) originally attributed to the Early Bronze Age Okunevo culture were from later periods.

A corrected version of Minusinsk Basin panel in Figure 4 with the incorrect data points removed is shown below as Figure 1. The incorporation of these new data does not change our original findings.

**Figure 1:**
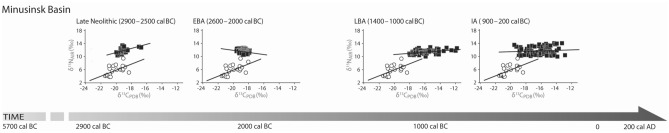
A corrected version of Minusinsk Basin panel from the original Figure 4.
