# Supplementary material for: Intensification in pastoralist cereal use coincides with the expansion of trans-regional networks in the Eurasian Steppe
Source: Sci Rep. 2019 Jun 10;9:8363. doi: 10.1038/s41598-018-35758-w (PMC6557825; doi:10.1038/s41598-018-35758-w)
Supplement: Supplementary file 1 — Figure S1, Figure S2, Figure S3, Figure S4, Figure S5, Figure S6, Table S1, Table S2, Table S3, Table S4, Table S5 [file 41598_2018_35758_MOESM1_ESM.pdf]

# Intensification in pastoralist cereal use coincides with the expansion of trans-regional networks in the Eurasian Steppe

Alicia R. Ventresca Miller[1,2*] and Cheryl A. Makarewicz[1]

[1]Institute for Prehistoric and Protohistoric Archaeology, Archaeological Stable Isotope Laboratory, Graduate School Human Development in Landscapes, Christian Albrechts University of Kiel, Johanna Mestorf Strasse 2-6, 24118 Kiel, Germany
[2]Max Planck Institute for the Science of Human History, Department of Archaeology, Kahlaische Strasse 10, 07745 Jena, Germany

+49 3641686742 ventrescamiller@shh.mpg.de Twitter: @steppebioarch
*Corresponding Author

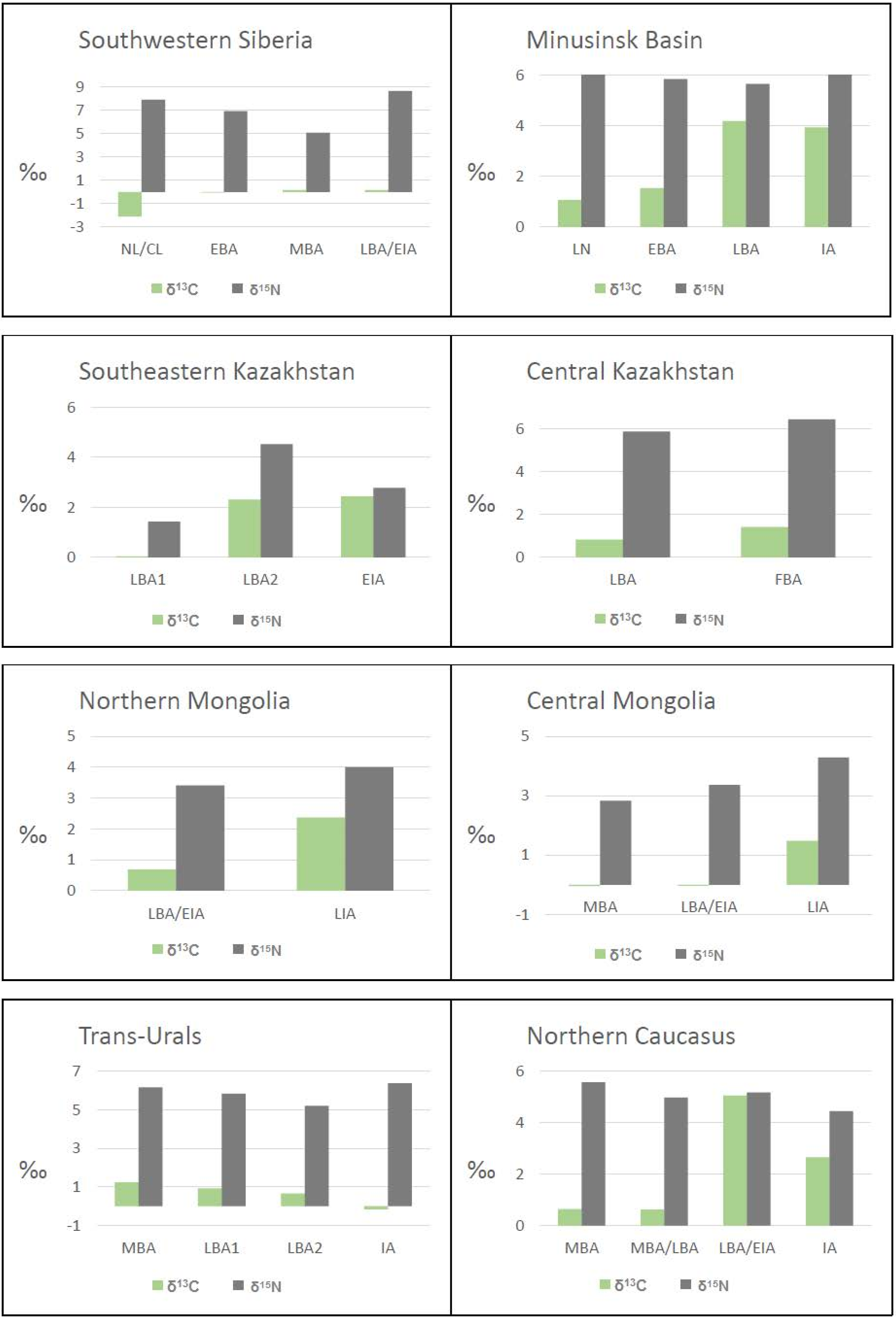
Southwestern Siberia
9
7
5
3
1
-1
-3
‰
NL/CL
EBA
MBA
LBA/EIA
δ13C
δ15N
Minusinsk Basin
6
4
2
0
‰
LN
EBA
LBA
IA
δ13C
δ15N
Southeastern Kazakhstan
6
4
2
0
‰
LBA1
LBA2
EIA
δ13C
δ15N
Central Kazakhstan
6
4
2
0
‰
LBA
FBA
δ13C
δ15N
Northern Mongolia
5
4
3
2
1
0
‰
LBA/EIA
LIA
δ13C
δ15N
Central Mongolia
5
3
1
-1
‰
MBA
LBA/EIA
LIA
δ13C
δ15N
Trans-Urals
7
5
3
1
-1
‰
MBA
LBA1
LBA2
IA
δ13C
δ15N
Northern Caucasus
6
4
2
0
‰
MBA
MBA/LBA
LBA/EIA
IA
δ13C
δ15N

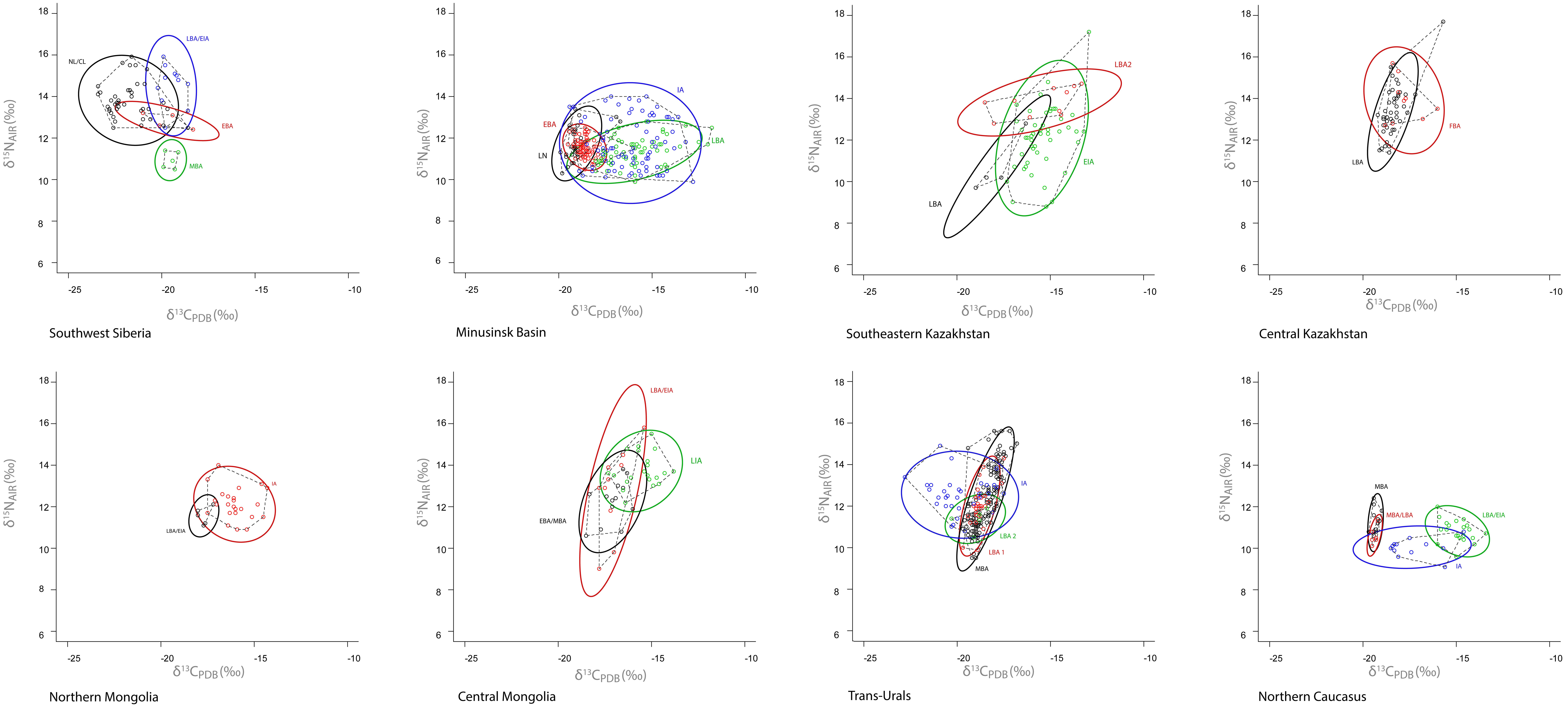

Southwest Siberia
NL/CL
LBA/EIA
EBA
MBA
Minusinsk Basin
IA
EBA
LN
LBA
Southeastern Kazakhstan
LBA2
EIA
LBA
Central Kazakhstan
FBA
LBA
Northern Mongolia
IA
LBA/EIA
Central Mongolia
LBA/EIA
LIA
EBA/MBA
Trans-Urals
IA
LBA 2
LBA 1
MBA
Northern Caucasus
MBA
MBA/LBA
LBA/EIA
IA
δ15NAIR (‰)
δ13CPDB (‰)

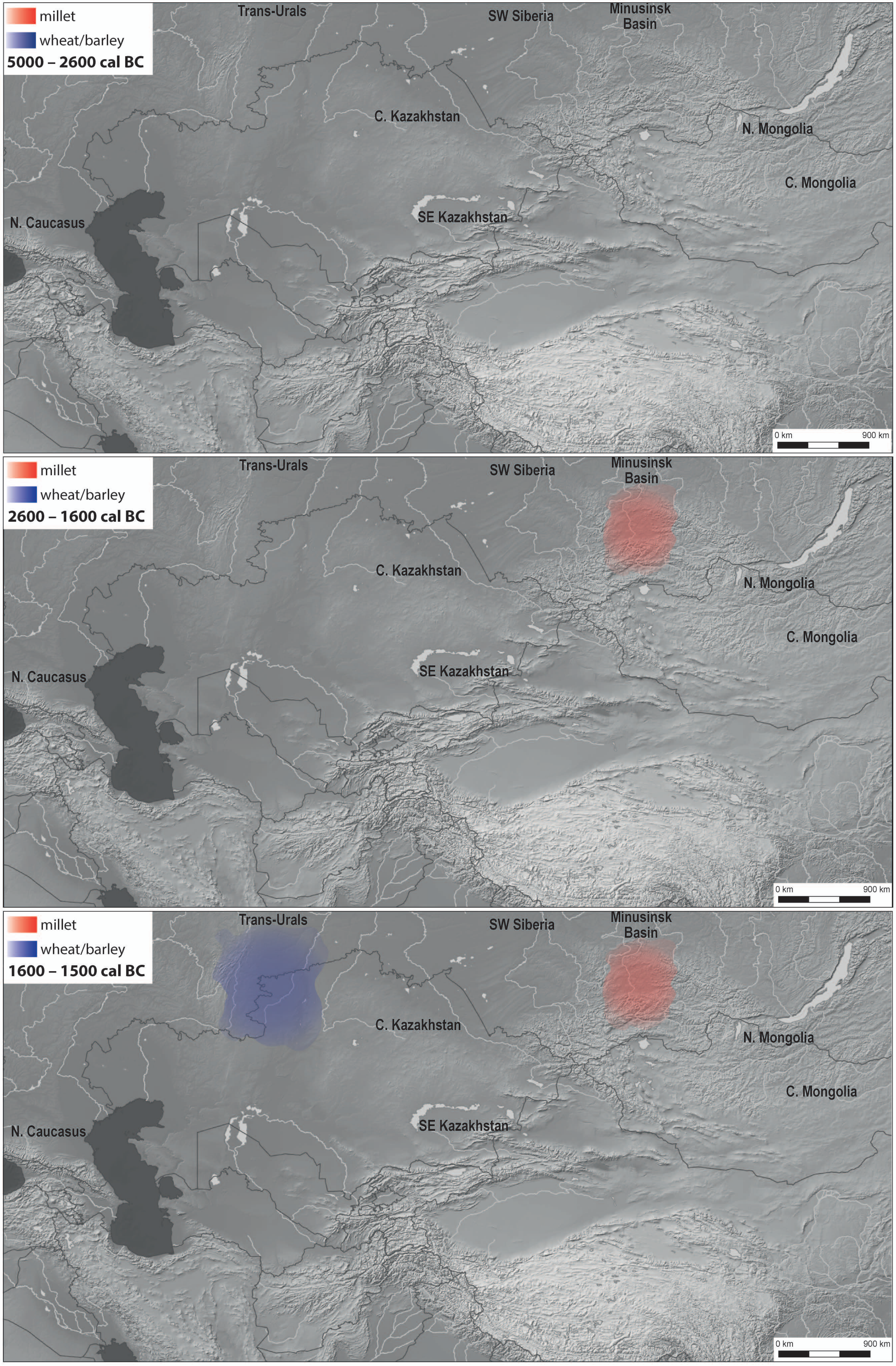

millet
wheat/barley
5000 – 2600 cal BC
Trans-Urals
SW Siberia
Minusinsk Basin
C. Kazakhstan
N. Mongolia
C. Mongolia
N. Caucasus
SE Kazakhstan
0 km
900 km
millet
wheat/barley
2600 – 1600 cal BC
Trans-Urals
SW Siberia
Minusinsk Basin
C. Kazakhstan
N. Mongolia
C. Mongolia
N. Caucasus
SE Kazakhstan
0 km
900 km
millet
wheat/barley
1600 – 1500 cal BC
Trans-Urals
SW Siberia
Minusinsk Basin
C. Kazakhstan
N. Mongolia
C. Mongolia
N. Caucasus
SE Kazakhstan
0 km
900 km

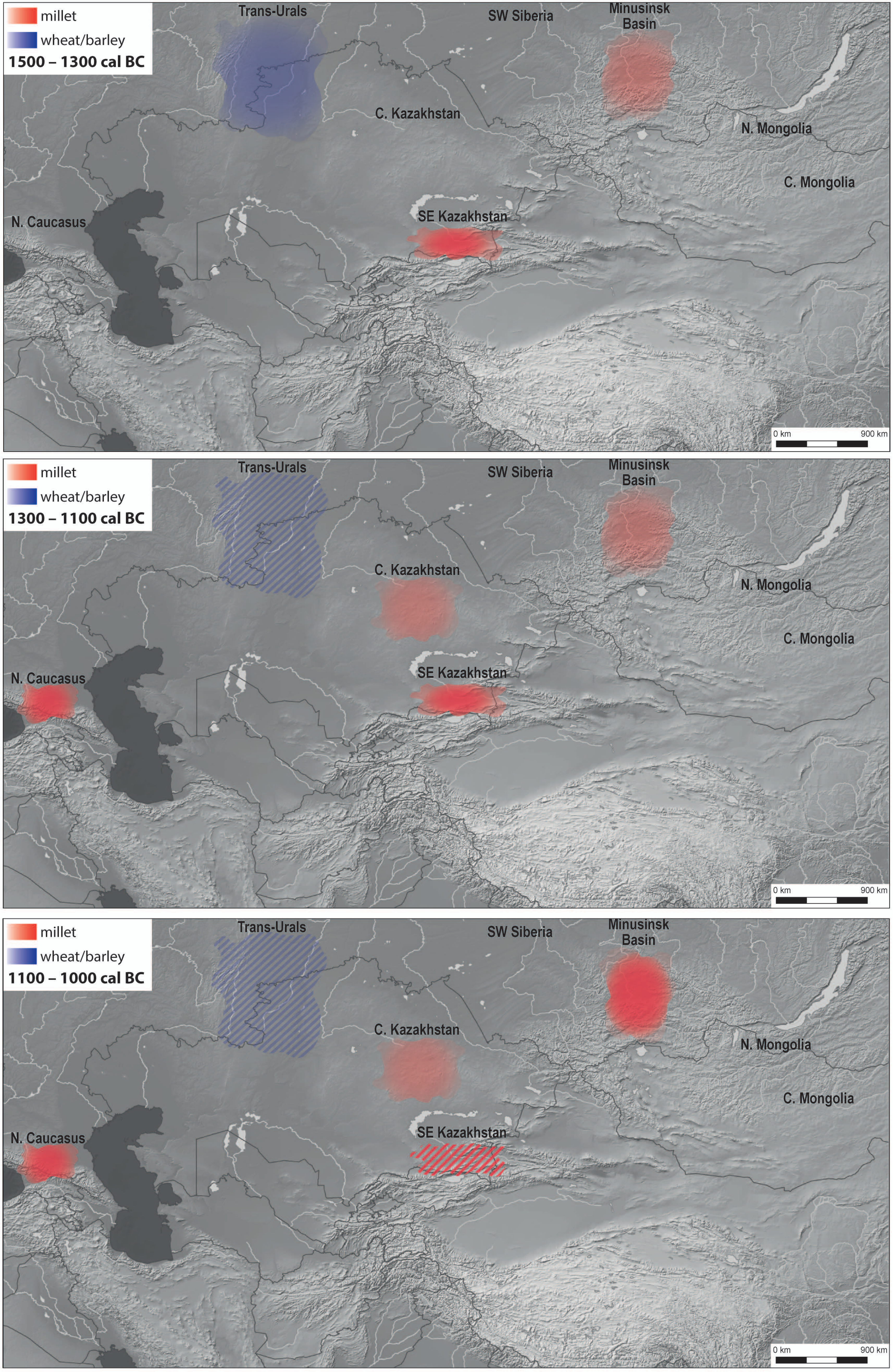
millet
wheat/barley
1500 – 1300 cal BC
Trans-Urals
SW Siberia
Minusinsk
Basin
C. Kazakhstan
N. Mongolia
C. Mongolia
N. Caucasus
SE Kazakhstan
0 km
900 km
millet
wheat/barley
1300 – 1100 cal BC
Trans-Urals
SW Siberia
Minusinsk
Basin
C. Kazakhstan
N. Mongolia
C. Mongolia
N. Caucasus
SE Kazakhstan
0 km
900 km
millet
wheat/barley
1100 – 1000 cal BC
Trans-Urals
SW Siberia
Minusinsk
Basin
C. Kazakhstan
N. Mongolia
C. Mongolia
N. Caucasus
SE Kazakhstan
0 km
900 km

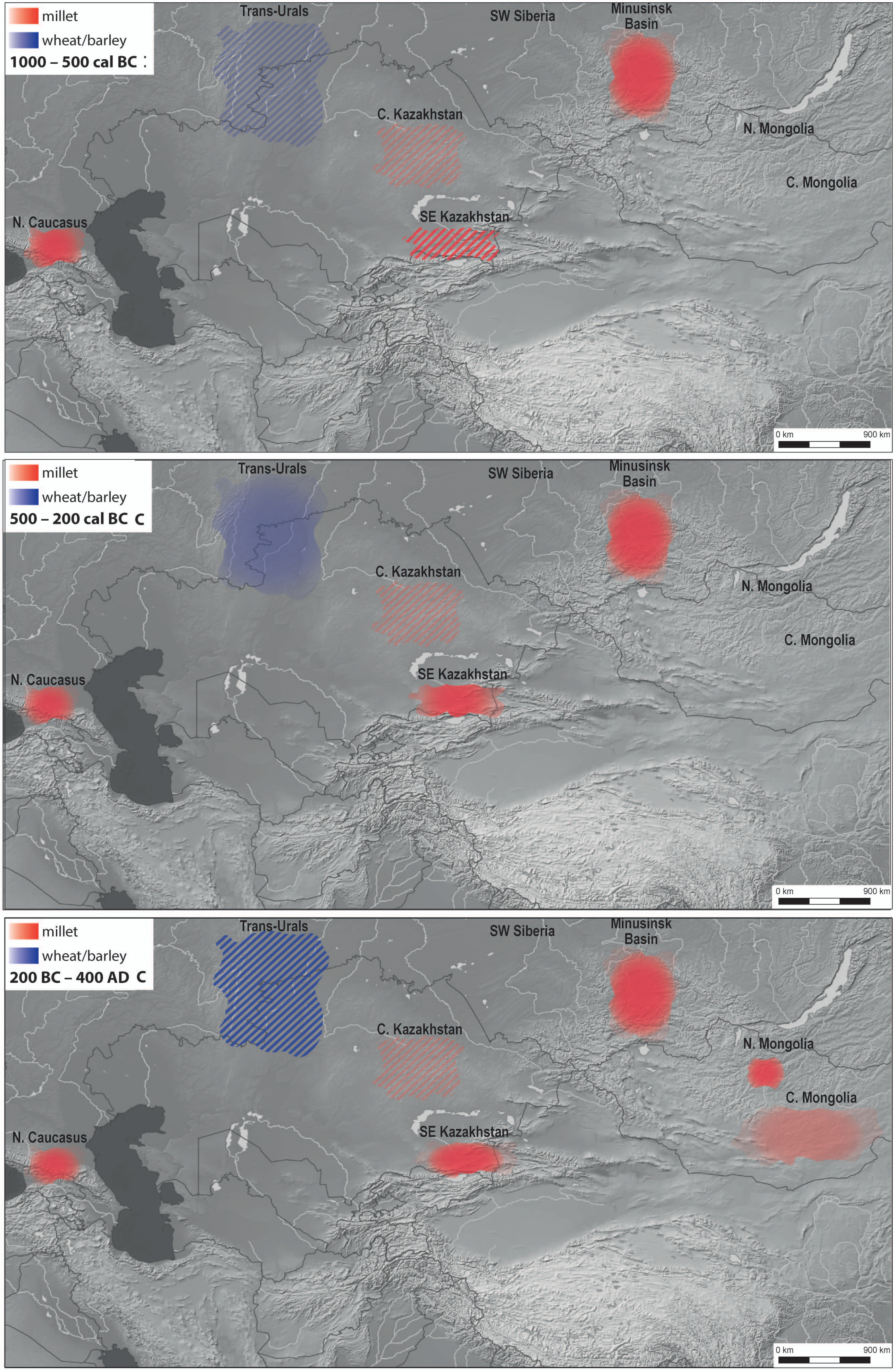
millet
wheat/barley
1000 – 500 cal BC
Trans-Urals
SW Siberia
Minusinsk Basin
C. Kazakhstan
N. Mongolia
C. Mongolia
SE Kazakhstan
N. Caucasus
0 km
900 km
millet
wheat/barley
500 – 200 cal BC C
Trans-Urals
SW Siberia
Minusinsk Basin
C. Kazakhstan
N. Mongolia
C. Mongolia
SE Kazakhstan
N. Caucasus
0 km
900 km
millet
wheat/barley
200 BC – 400 AD C
Trans-Urals
SW Siberia
Minusinsk Basin
C. Kazakhstan
N. Mongolia
C. Mongolia
SE Kazakhstan
N. Caucasus
0 km
900 km

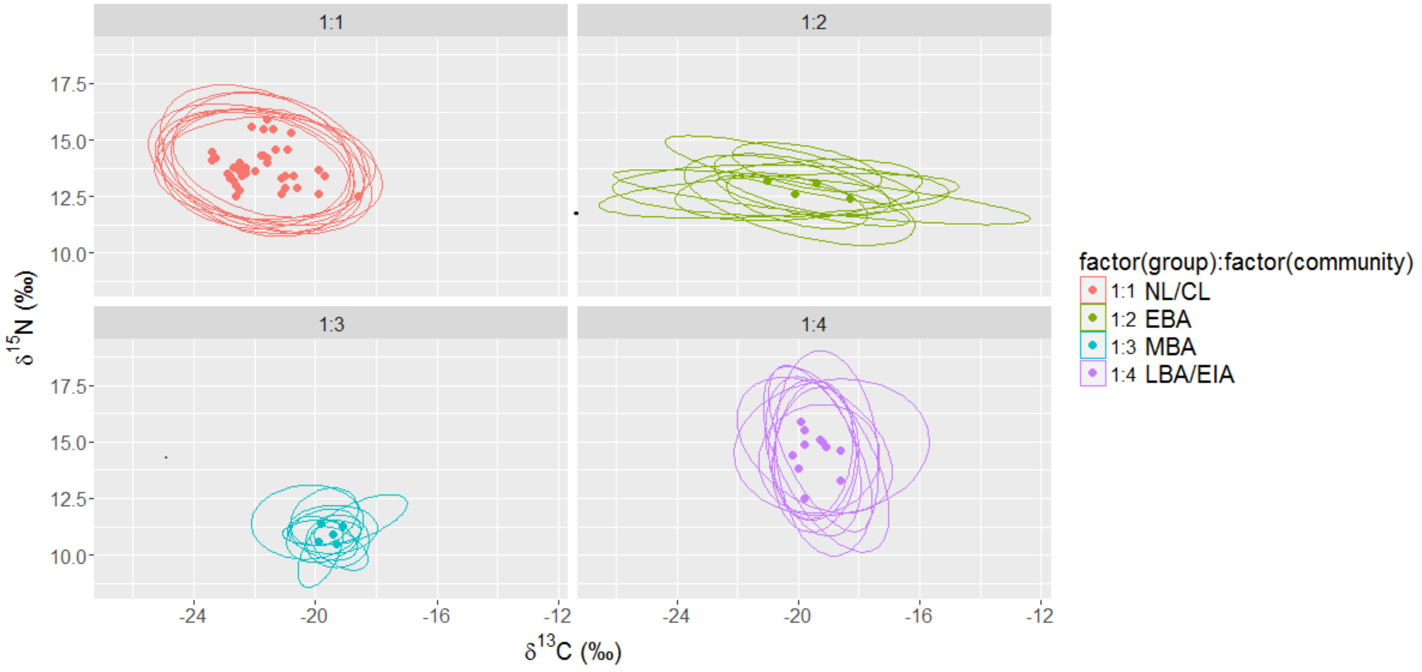


S6.1 $SEA_B$ (Baysian standard ellipse area) iterations (at 99% confidence) for humans within each time period in Southwestern Siberia

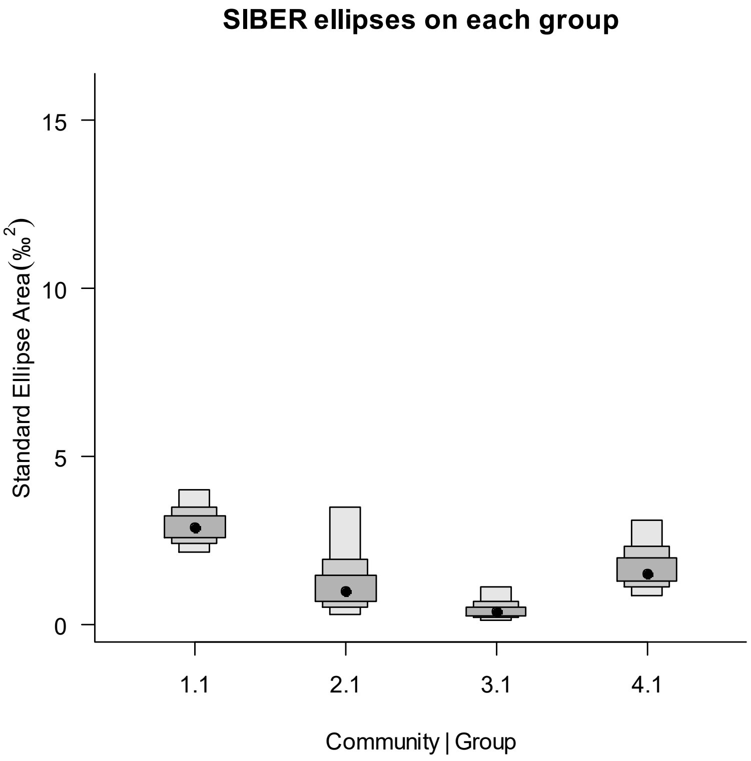


S6.2 Density plots for Southwest Siberia indicating the confidence intervals of SIBER ellipses for each human group. Black dots correspond to the mean standard ellipse area for each group while grey box areas reflect 95, 75, and 50% confidence intervals for each human group respectively.

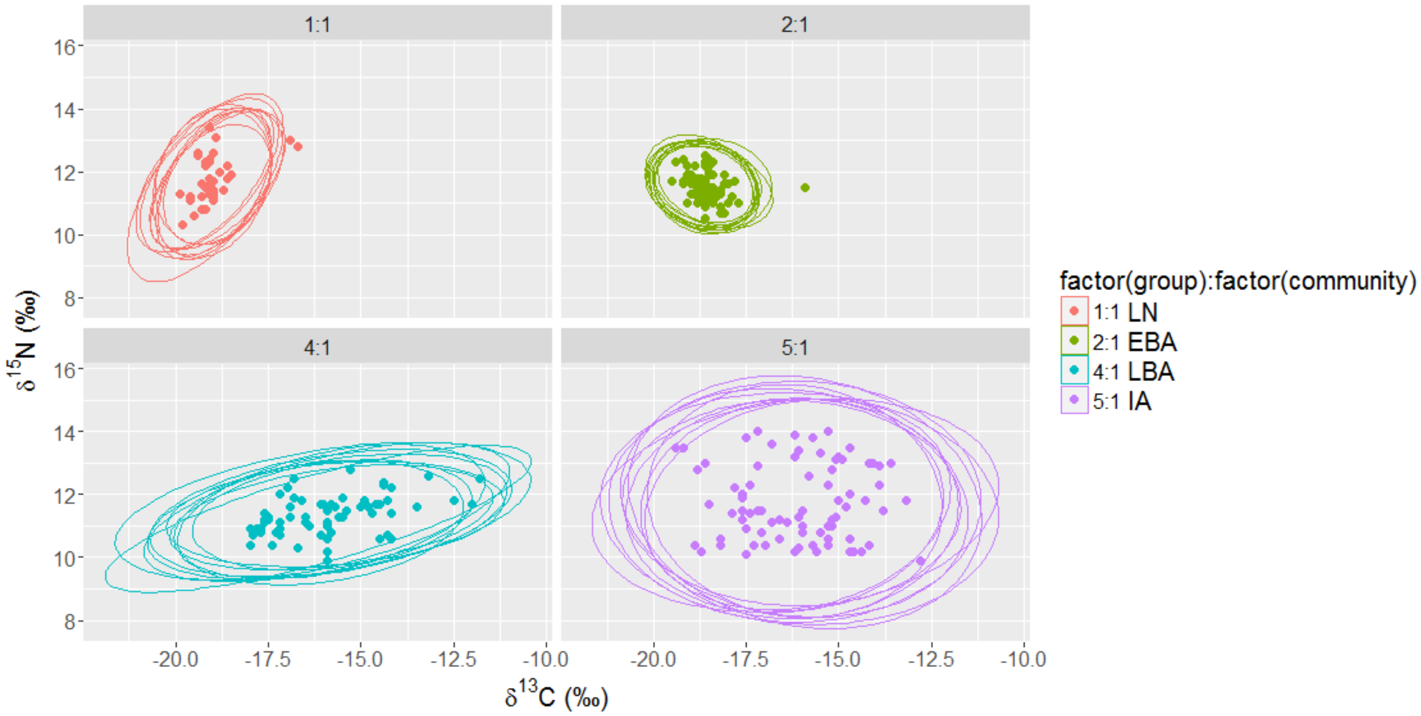


S6.3 $SEA_B$ (Baysian standard ellipse area) iterations (at 99% confidence) for humans within each time period in the Minusinsk Basin

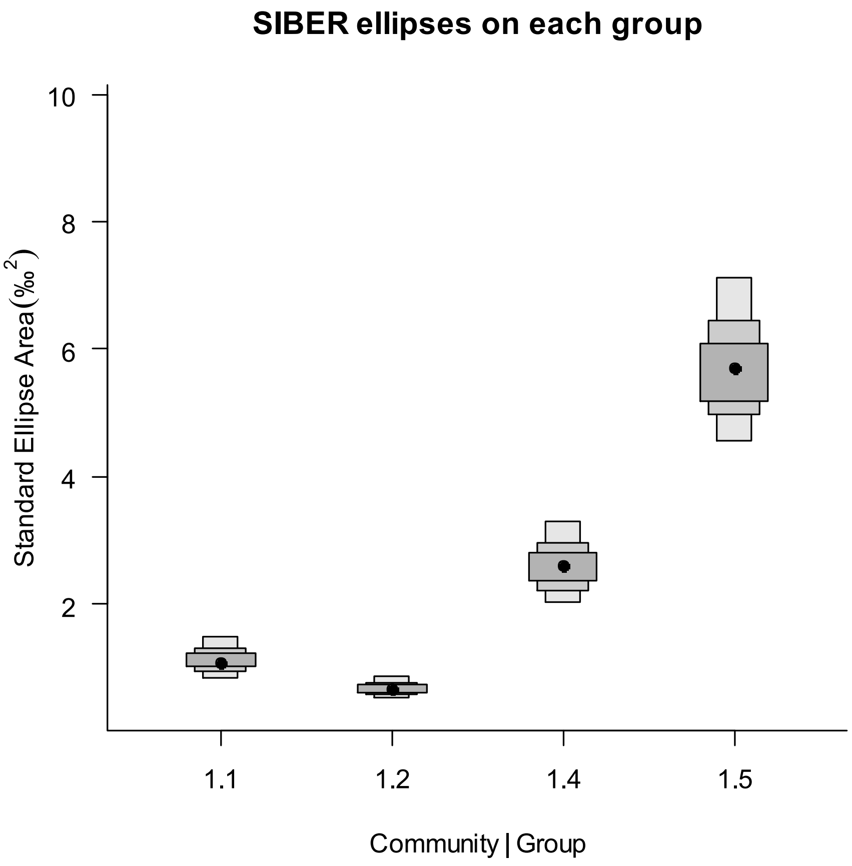


S6.4 Density plots for the Minusinsk Basin indicating the confidence intervals of SIBER ellipses for each human group. Black dots correspond to the mean standard ellipse area for each group while grey box areas reflect 95, 75, and 50% confidence intervals for each human group respectively.

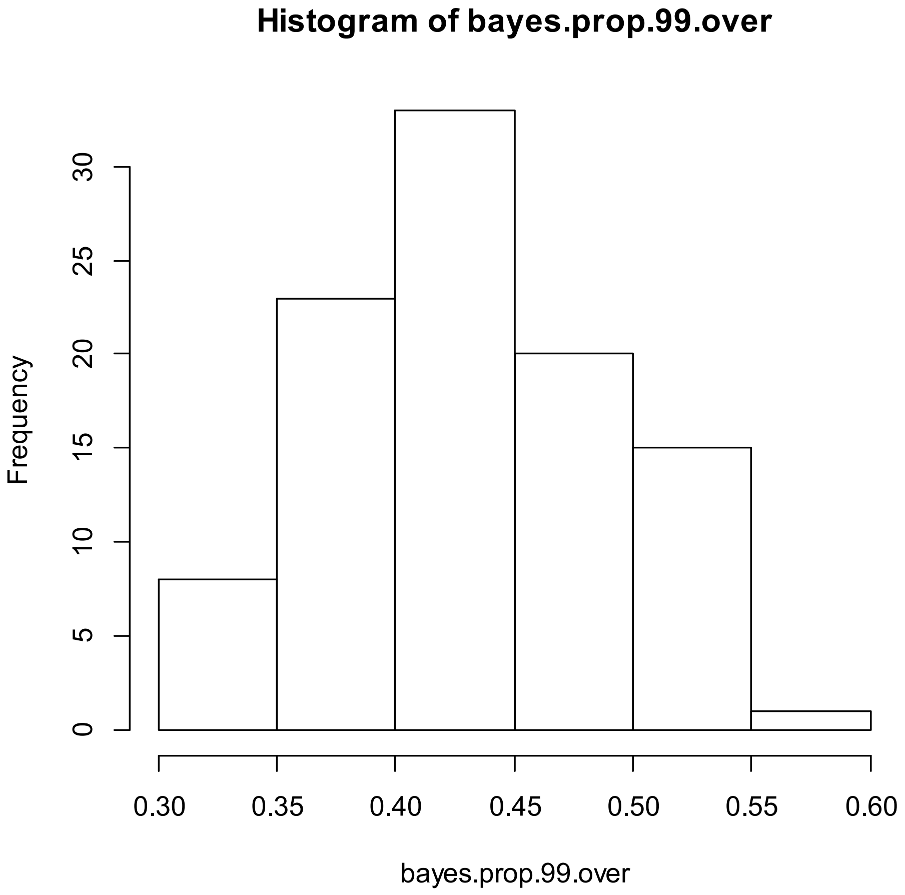


S6.5 Percent overlap of Bayesian standard ellipse areas for the LN and EBA in the Minusinsk Basin. This reflects the maximum percent of overlap of the two $SEA_B$ plots which have a 99% probability of overlapping at less than 60%.

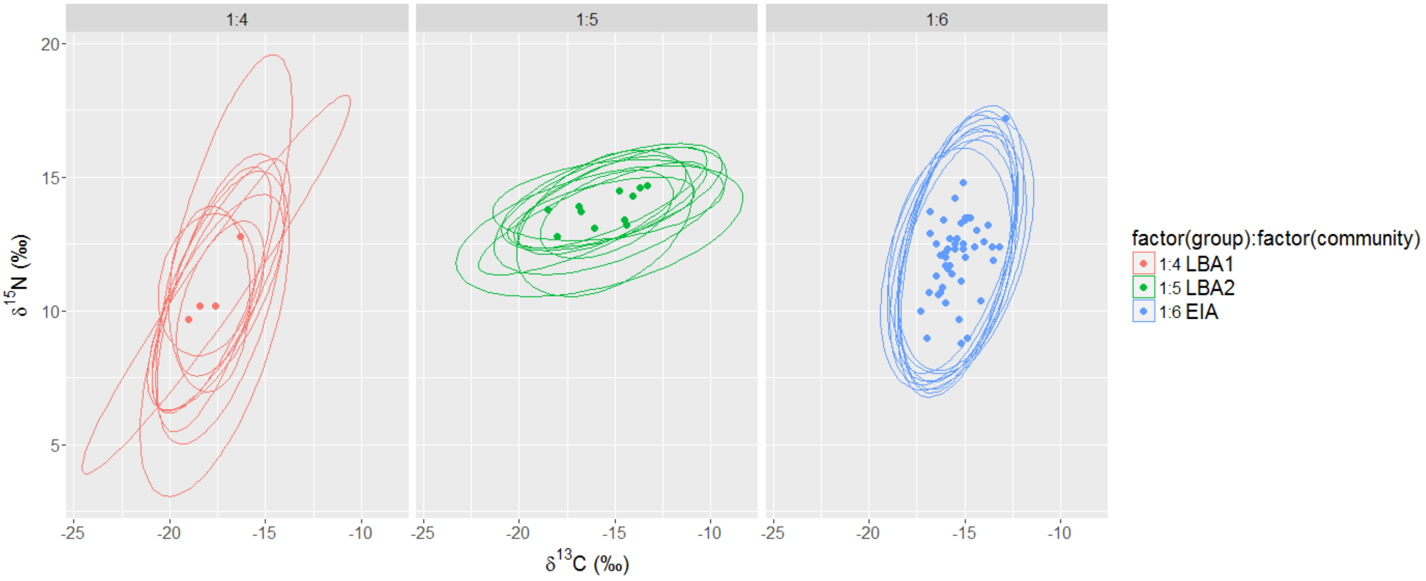


S6.6 $SEA_B$ (Baysian standard ellipse area) iterations (at 99% confidence) for humans within each time period in Southeastern Kazakhstan

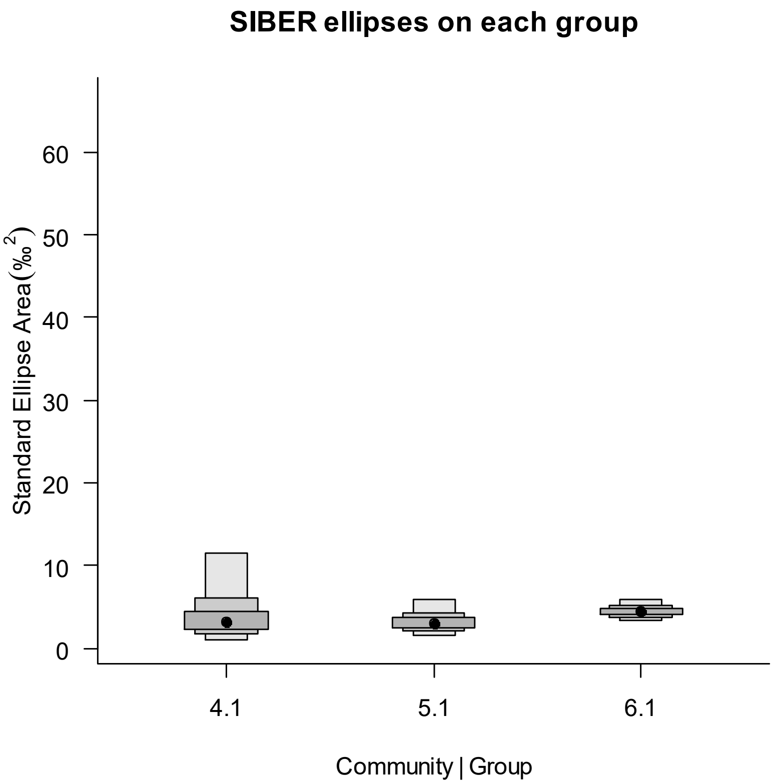


S6.7 Density plots for Southeastern Kazakhstan indicating the confidence intervals of SIBER ellipses for each human group. Black dots correspond to the mean standard ellipse area for each group while grey box areas reflect 95, 75, and 50% confidence intervals for each human group respectively.

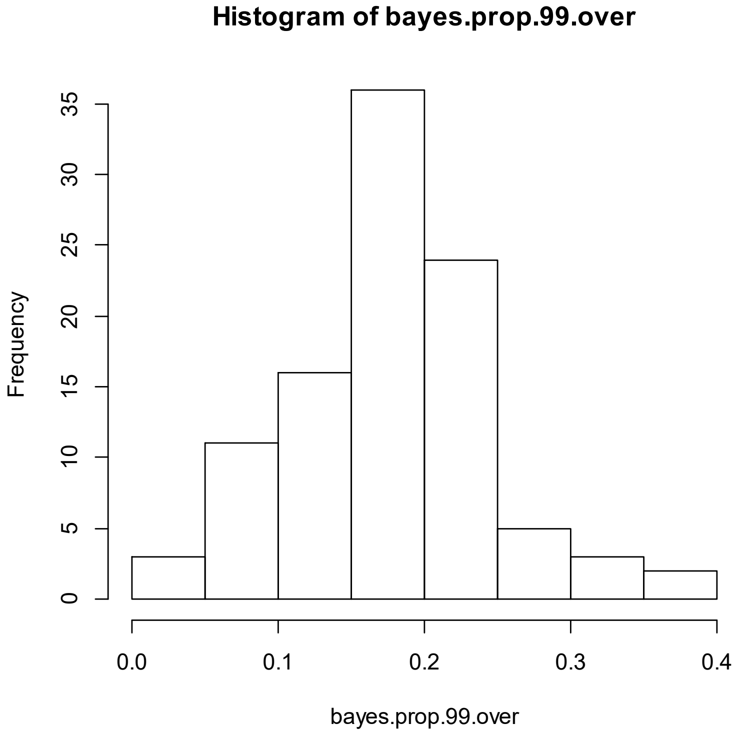


S6.8 Percent overlap of Bayesian standard ellipse areas for LBA1 and LBA2 in Southeastern Kazakhstan. This reflects the maximum percent of overlap of the two $SEA_B$ plots which have a 99% probability of overlapping at less than 40%.

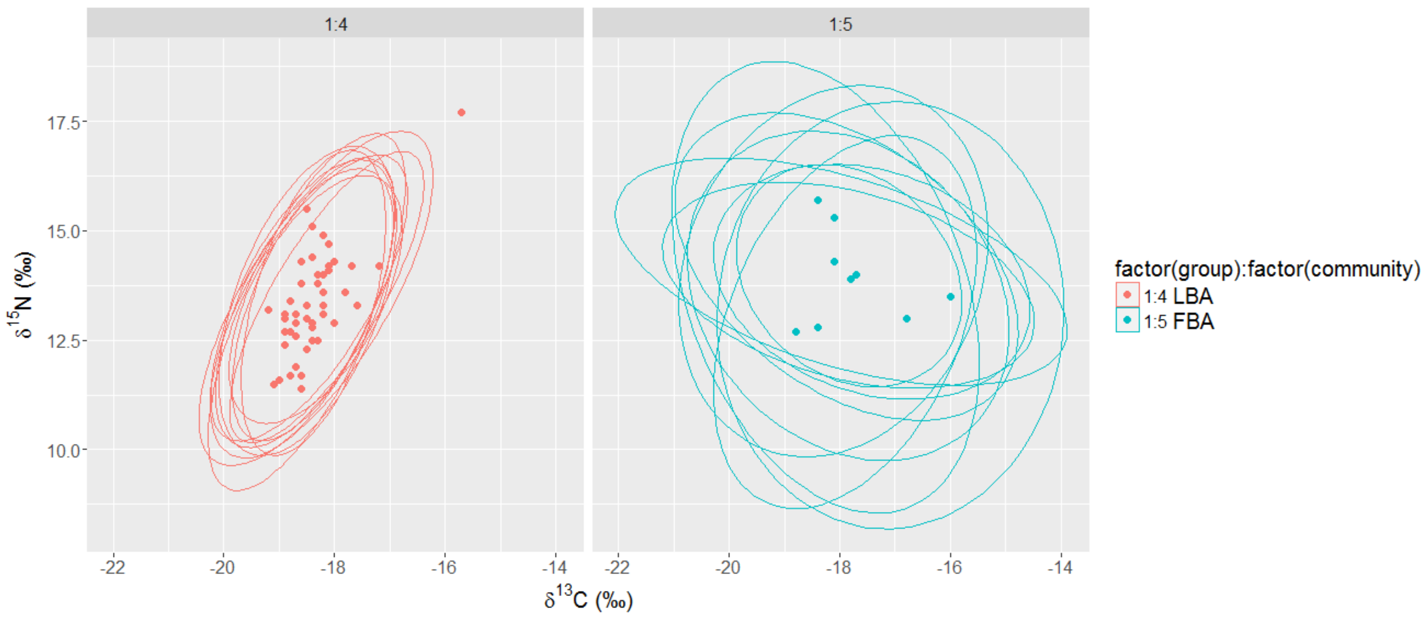


S6.9 $SEA_B$ (Baysian standard ellipse area) iterations (at 99% confidence) for humans within each time period in Central Kazakhstan

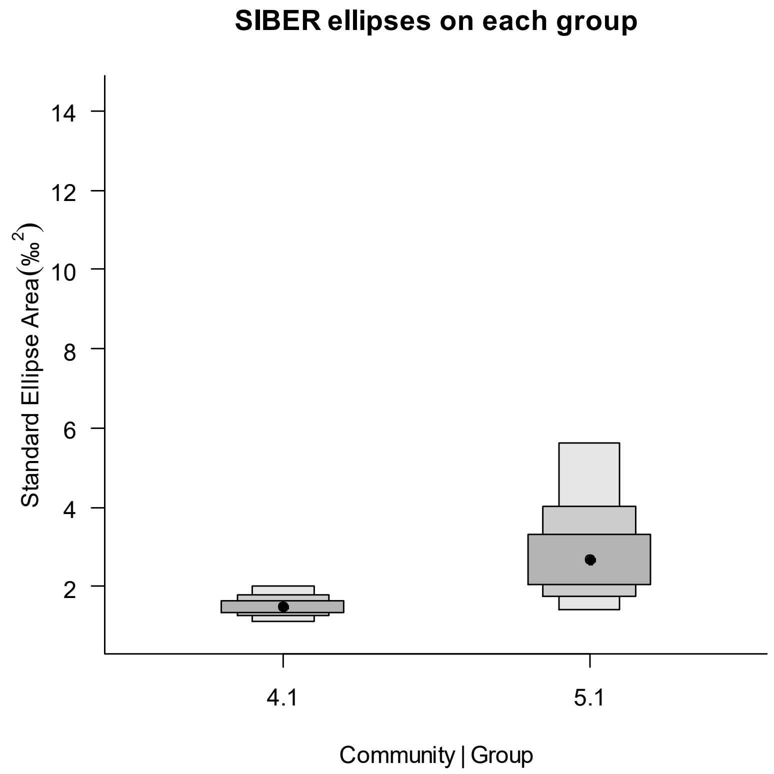


S6.10 Density plots for Central Kazakhstan indicating the confidence intervals of SIBER ellipses for each human group. Black dots correspond to the mean standard ellipse area for each group while grey box areas reflect 95, 75, and 50% confidence intervals for each human group respectively.

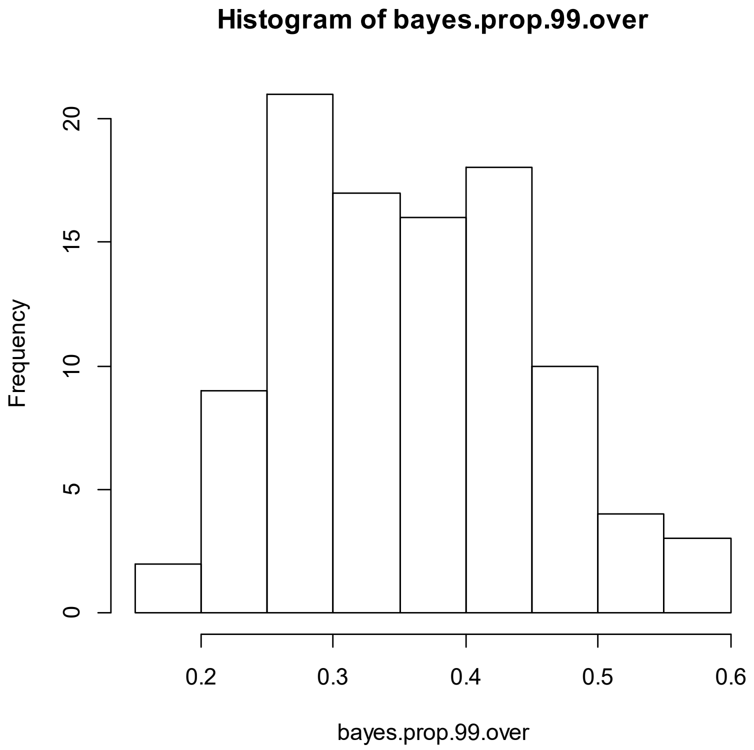


S6.11 Percent overlap of Bayesian standard ellipse areas for LBA and FBA in Central Kazakhstan. This reflects the maximum percent of overlap of the two $SEA_B$ plots which have a 99% probability of overlapping at less than 60%.

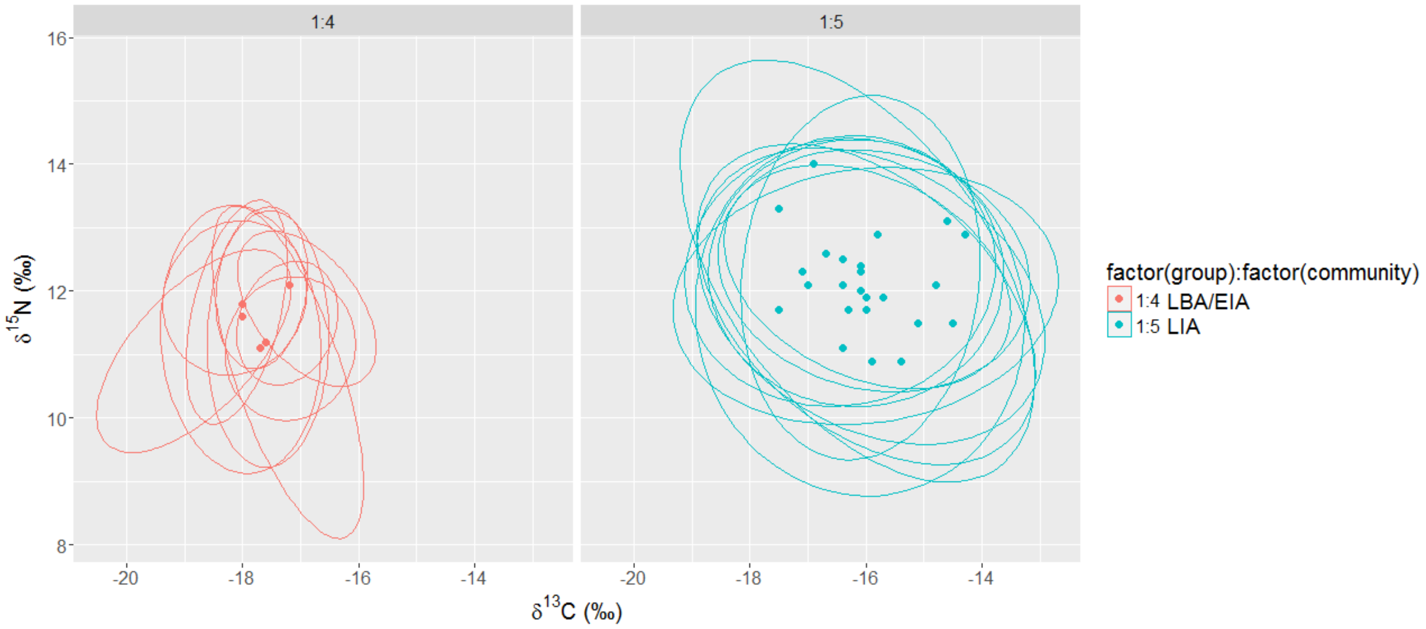


S6.12 $SEA_B$ (Baysian standard ellipse area) iterations (at 99% confidence) for humans within each time period in Northern Mongolia

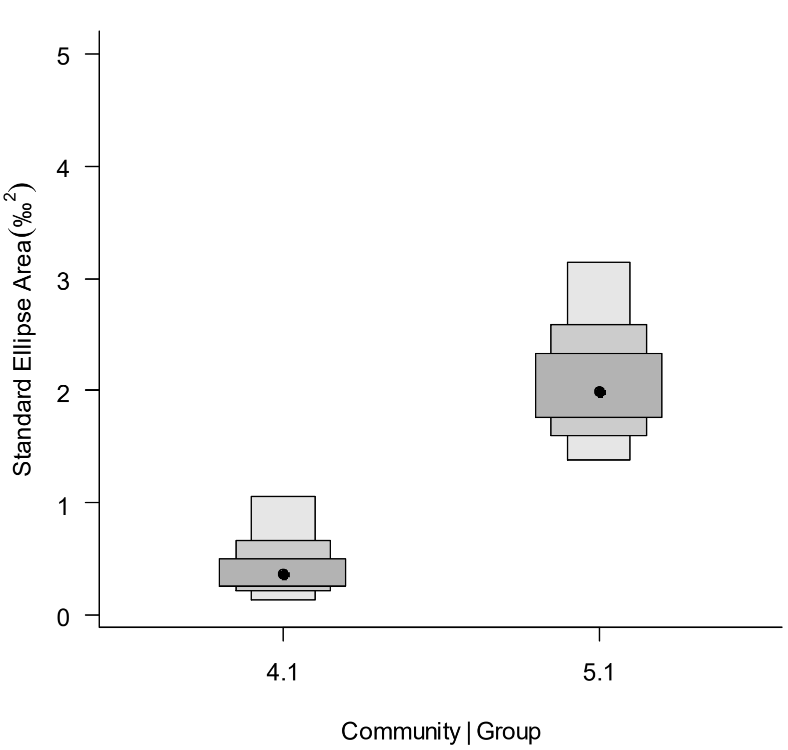


S6.13 Density plots for Northern Mongolia indicating the confidence intervals of SIBER ellipses for each human group. Black dots correspond to the mean standard ellipse area for each group while grey box areas reflect 95, 75, and 50% confidence intervals for each human group respectively.

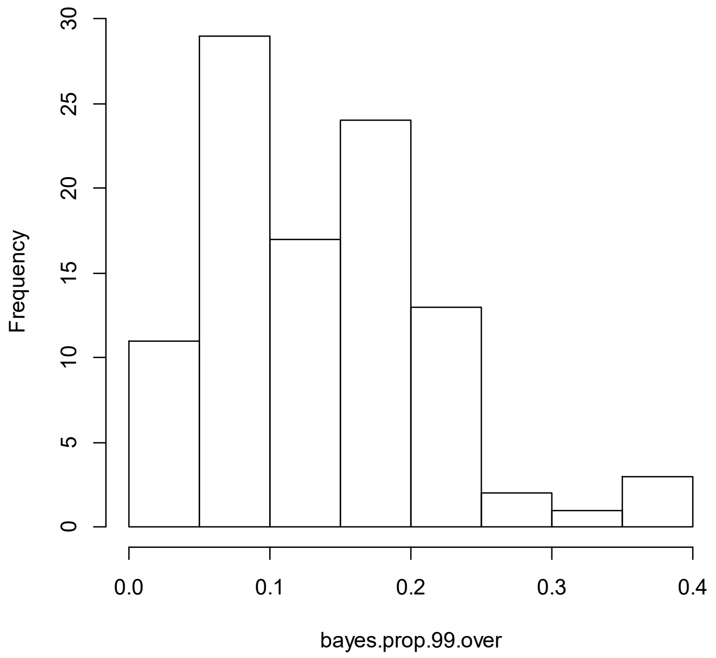


S6.14 Percent overlap of Bayesian standard ellipse areas for LBA/EIA and LIA in Northern Mongolia. This reflects the maximum percent of overlap of the two $SEA_B$ plots which have a 99% probability of overlapping at less than 40%.

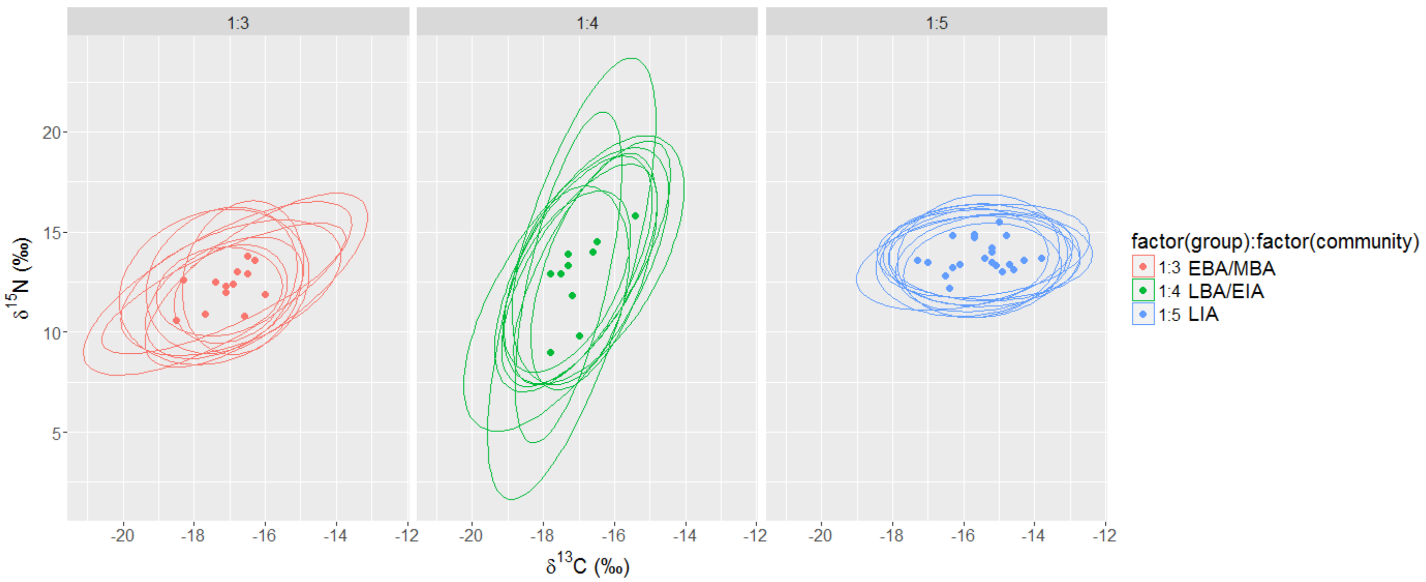


S6.15 $SEA_B$ (Baysian standard ellipse area) iterations (at 99% confidence) for humans within each time period in Central Mongolia

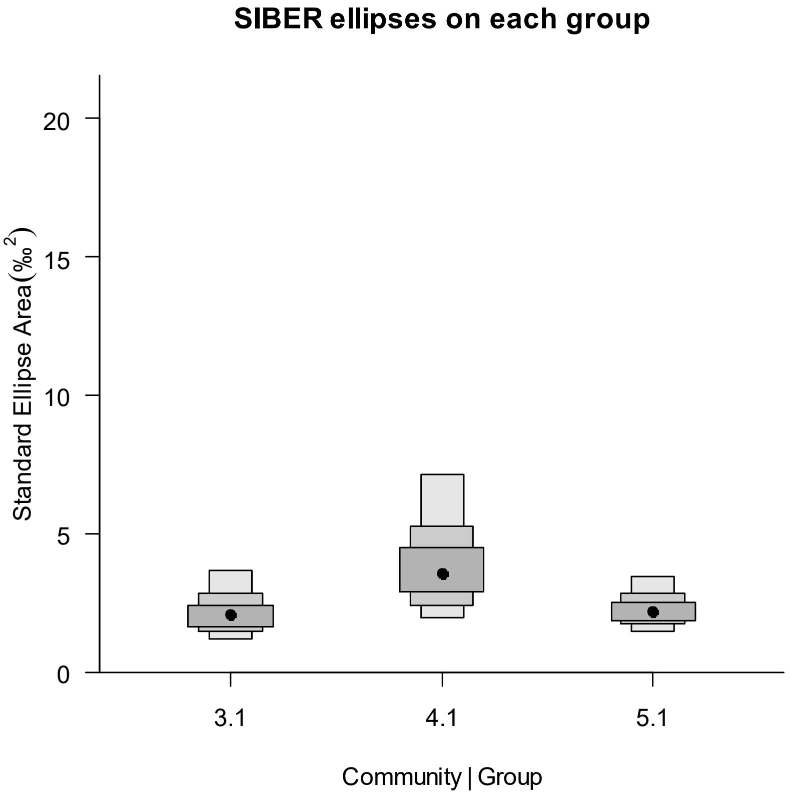


S6.16 Density plots for Central Mongolia indicating the confidence intervals of SIBER ellipses for each human group. Black dots correspond to the mean standard ellipse area for each group while grey box areas reflect 95, 75, and 50% confidence intervals for each human group respectively.

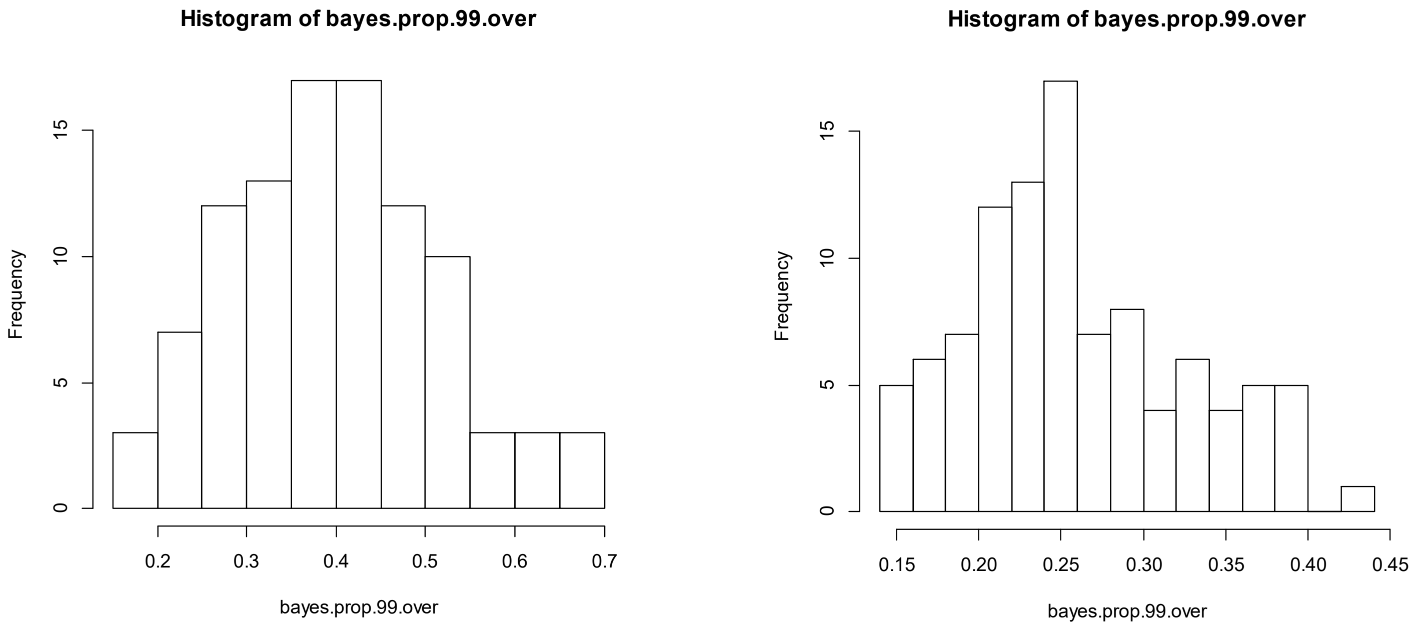


S6.17 Percent overlap of Bayesian standard ellipse areas for EBA/MBA and LBA/EIA (left) in Central Mongolia. This reflects the maximum percent of overlap of the two $SEA_B$ plots which have a 99% probability of overlapping up to 70%.

Percent overlap of Bayesian standard ellipse areas for LBA/EIA and LIA (right) in Central Mongolia. This reflects the maximum percent of overlap of the two $SEA_B$ plots which have a 99% probability of overlapping less than 45%.

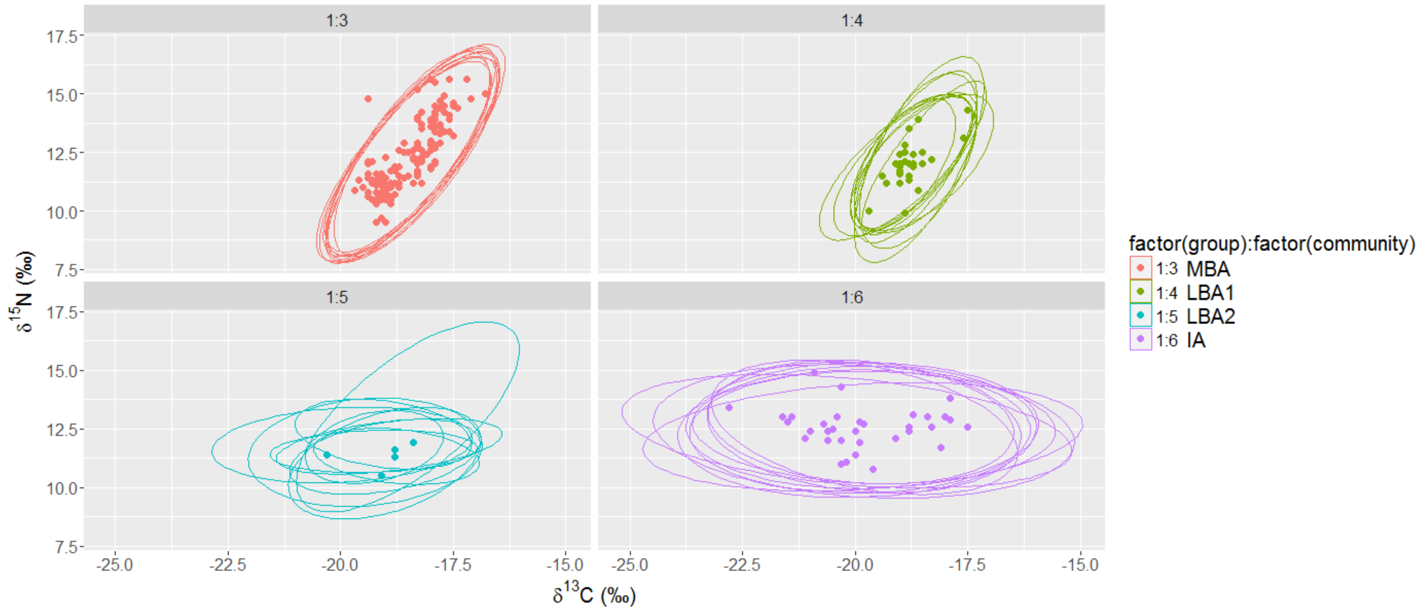


S6.18 $SEA_B$ (Baysian standard ellipse area) iterations (at 99% confidence) for humans within each time period in the Trans-Urals

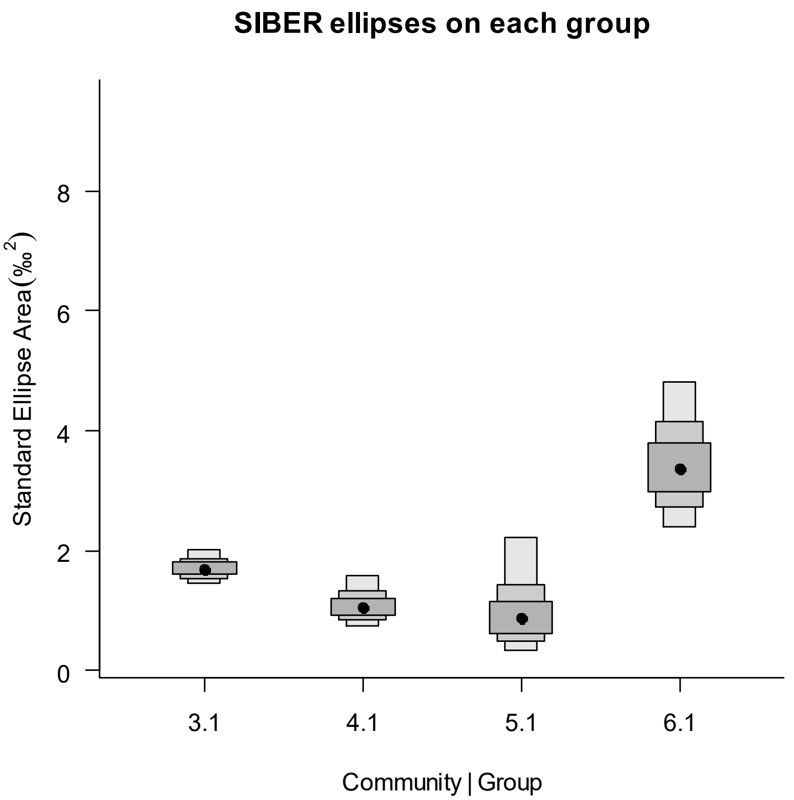


S6.19 Density plots for the Trans-Urals indicating the confidence intervals of SIBER ellipses for each human group. Black dots correspond to the mean standard ellipse area for each group while grey box areas reflect 95, 75, and 50% confidence intervals for each human group respectively.

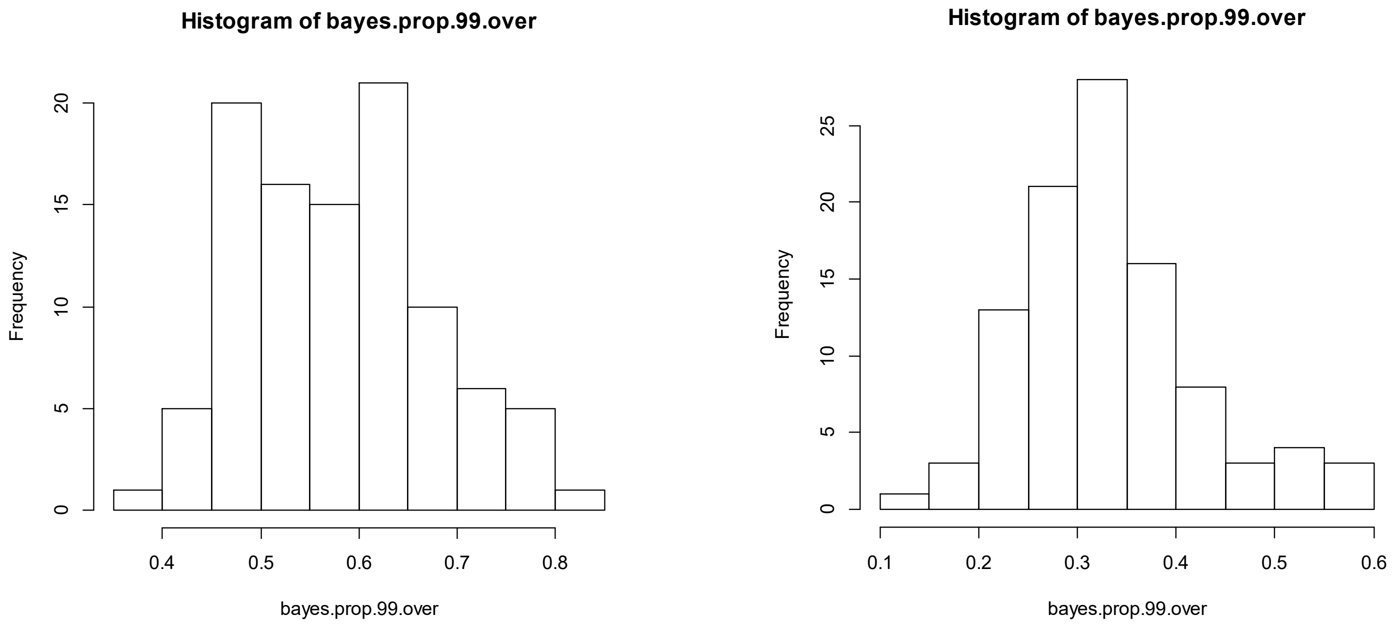


S6.20 Percent overlap of Bayesian standard ellipse areas for MBA and LBA1 (left) in the Trans-Urals. This reflects the maximum percent of overlap of the two $SEA_B$ plots which have a 99% probability of overlapping up to 85%.

Percent overlap of Bayesian standard ellipse areas for LBA1 and LBA2 (right) in the Trans-Urals. This reflects the maximum percent of overlap of the two $SEA_B$ plots which have a 99% probability of overlapping less than 60%.

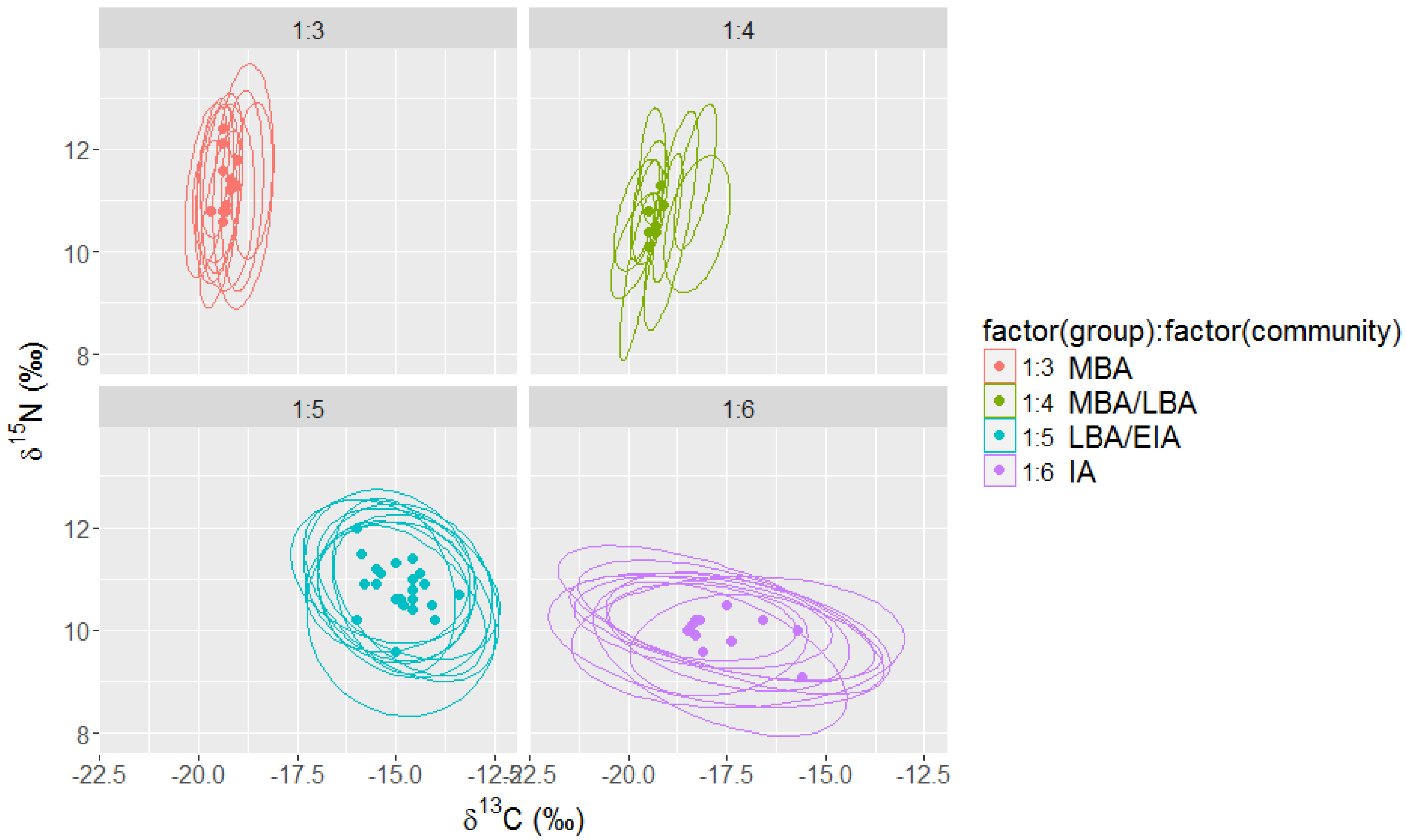


S6.21 $SEA_B$ (Baysian standard ellipse area) iterations (at 99% confidence) for humans within each time period in the Northern Caucasus

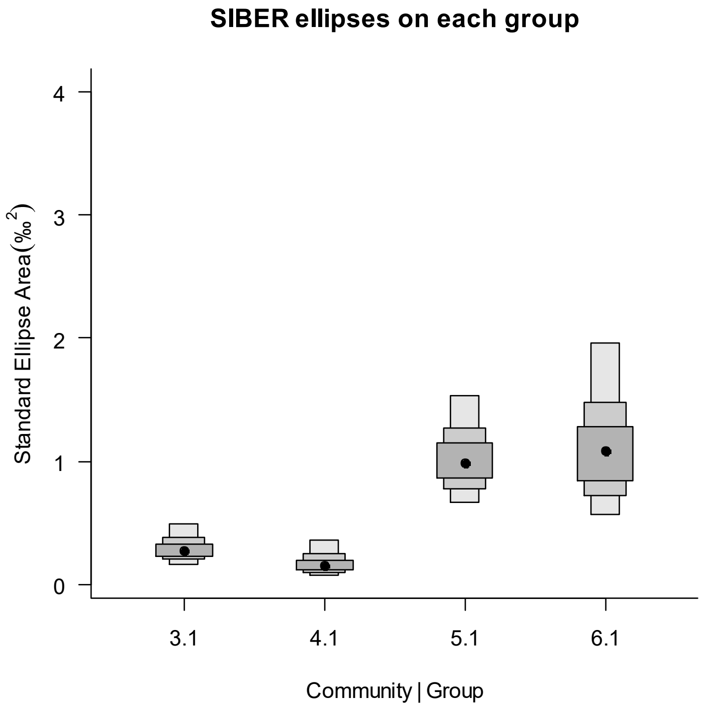


S6.22 Density plots for the Northern Caucasus indicating the confidence intervals of SIBER ellipses for each human group. Black dots correspond to the mean standard ellipse area for each group while grey box areas reflect 95, 75, and 50% confidence intervals for each human group respectively.

| Location | Sites for Stable Isotopic Data | Time Period | Archeological Culture(s) | Dates | Stable Isotope Citations | Radiocarbon Citations |
|---|---|---|---|---|---|---|
| Northern Caucasus | Kabardinka, Kudachurt, Zaragizh | Middle Bronze Age | Northcaucasian culture | 2570-1889 cal. BC | Knipper et al. in press; Holland et al. 2010 | Knipper et al. in press |
| Northern Caucasus | Kudachurt K14 | Middle Bronze Age / Late Bronze Age | Central caucasian culture | 1925-1784 cal. BC | Knipper et al. in press | Knipper et al. in press |
| Northern Caucasus | Klin-Yar | Late Bronze Age / Early Iron Age | Koban | 1300-1100 cal. BC | Higham et al. 2010 | Higham et al. 2010 |
| Northern Caucasus | Klin-Yar | Iron Age | Sarmatian | 200 BC - 400 AD | Higham et al. 2010 | Higham et al. 2010 |
| Trans-Urals | Kamennyi Ambar V, Bolshekaragansky, Bestamak, Novoilinka | Middle Bronze Age | Sintashta / Sintashta-Petrovka | 2050-1639 cal. BC | Privat 2004; Ventresca Miller et al. 2014; Motuzaite Matuzeviciute et al. 2015; Hanks et al. in press | Hanks et al. 2007; Shevnina et al. 2013 |
| Trans-Urals | Lisakovsk | Late Bronze Age (LBA1) | Andronovo | 1860-1680 cal. BC | Ventresca Miller et al. 2014 | Panyushkina et al. 2010 |
| Trans-Urals | Isiney | Late Bronze Age (LBA2) | Andronovo | 1800-1400 BC | Privat 2004 | Privat 2004 |
| Trans-Urals | Kurtuguz I, Murzino 1, Skaty 1, Shaidurikha, Gayovsky I, Pobedy | Iron Age | Gorkhovo, Sargat-Gorkhovo, Sargat, Sarmatian | 700-300 BC | Privat 2004 | Privat 2004; Hanks 2002 |
| Central Kazakhstan | Tengiszhol, Temirkash, Nurtaldy, Dariya, Tashik, Aschisu, Ayap-bergen, Kopa-1, Akimbek, Tasyrbai 2, Kyzylkol, Kairakty | Late Bronze Age | Andronovo (Early Alakul', Alakul', Fedorovo) | 1800-1600 BC | Lightfoot et al. 2014 | Lightfoot et al. 2014 |
| Central Kazakhstan | Temirkash, Kudryavaya Sopka-1, Kyzyl, Karatugai, Kent | Final Bronze Age | Sargarinsko-Alekseevsk / Begazy-Dandybai | 1300-800 cal. BC | Lightfoot et al. 2014 | Lightfoot et al. 2014 |

| Location | Sites for Stable Isotopic Data | Time Period | Archeological Culture(s) | Dates | Stable Isotope Citations | Radiocarbon Citations |
|---|---|---|---|---|---|---|
| Southeastern Kazakhstan | Kyzyl Bulak | Late Bronze Age 2 | Andronovo | 1754-1536 cal. BC | Motuzaite Matuzeviciute et al. 2015 | Motuzaite Matuzeviciute et al. 2015 |
| Southeastern Kazakhstan | Oi-Dzailau VII | Late Bronze Age 1 | Andronovo | 1608-1427 cal. BC | Motuzaite Matuzeviciute et al. 2015 | Motuzaite Matuzeviciute et al. 2015 |
| Southeastern Kazakhstan | Karatuma, Kargaly 1, Alatau 1, Kamenka | Early Iron Age | Saka | 384-56 cal. BC | Motuzaite Matuzeviciute et al. 2015 | Motuzaite Matuzeviciute et al. 2015; Chang et al. 2003 |
| Southwestern Siberia | Itkul, Ust-Isha, Firsovo XI, Solnts 5, Tuzovskiye | Neolithic / Chalcolithic | Kuznetski-Altaisky / Bolshemysky | 5667-3701 cal. BC | Motuzaite Matuzeviciute et al. 2016 | Motuzaite Matuzeviciute et al. 2016 |
| Southwestern Siberia | Teleutsky | Early Bronze Age | Elunino | 2500-2200 cal. BC | Motuzaite Matuzeviciute et al. 2016 | Motuzaite Matuzeviciute et al. 2016 |
| Southwestern Siberia | Firsovo XIV | Middle Bronze Age | Andronovo (Fedorovo) | 1684-1512 cal. BC | Motuzaite Matuzeviciute et al. 2016 | Motuzaite Matuzeviciute et al. 2016 |
| Southwestern Siberia | Chicha | Late Bronze Age | Irmen | 1430 to 1120 cal. BC | Privat et al. 2005; Privat 2004 | Privat et al. 2005 |
| Minusinsk Basin | Afanasyeva Gora, Karasuk III | Late Neolithic | Afanasyev/Afanasyevo | 2900-2500 cal. BC | Svyatko et al. 2007; Svyatko et al. 2013 | Svyatko et al. 2009 |
| Minusinsk Basin | Uibat III, Uibat V, Verhniy Askiz I | Early Bronze Age | Okunev/ Okunevo | 2600-1900 cal. BC | Svyatko et al. 2007; Svyatko et al. 2013 | Svyatko et al. 2009 |
| Minusinsk Basin | Bateni Sovhoz, Melnichniy Log, Okunev Ulus, Solnechniy Log, Upper Karasuk River, Yarki | Late Bronze Age | Karasuk | 1400-1000 cal. BC | Svyatko et al. 2007; Svyatko et al. 2013 | Svyatko et al. 2009 |
| Minusinsk Basin | Bateni -Saragash, Chernoye Ozero I, Grishkin Log, Lepeshki, Nurilkov Ulus, Okunev Ulus, Podgornoye Ozero, Saragash, Saragshinskoe Ozero, Saragashinskiy Spusk, Yarki, Ai-Dai, Amyrlyg | Iron Age | Tagar, Uyuk | 900 BC-200 AD (Svyatko et al. 2013); 740-410 cal. BC (Murphy et al. 2013) | Svyatko et al. 2007; Svyatko et al. 2013; Murphy et al. 2013 | Svyatko et al. 2009; Murphy et al. 2013 |

| Location | Sites for Stable Isotopic Data | Time Period | Archeological Culture(s) | Dates | Stable Isotope Citations | Radiocarbon Citations |
|---|---|---|---|---|---|---|
| Northern Mongolia | Egiin Gol | Late Iron Age | Xiongnu | 200 cal. BC - 200 AD | Machicek 2011 | Honeychurch 2015 |
| Central Mongolia | Ulaanzuukh | Early Bronze Age / Middle Bronze Age | Ulaanzuukh-Tevsh Culture | 1493-1127 cal. BC | Fenner et al. 2014 | Fenner et al. 2014 |
| Central Mongolia | Daram uul, Tevsh, Baga Gazaryn Chuluu | Late Bronze Age / Early Iron Age | Slab Grave Culture | 1000-300 cal. BC | Machicek 2010; Yoneda et al. 2016 | Honeychurch 2015; Yoneda et al. 2016 |
| Central Mongolia | Baga Gazaryn Chuluu | Late Iron Age | Xiongnu | 200 BC - 200 AD | Machicek 2010; Makarewicz 2015 | Honeychurch 2015 |

**North Caucasus**

| *T-test Slopes* | *Time Period (Standardized)* | *df* | *p-value* | |
|---|---|---|---|---|
| MBA compared to Herbivores | Bronze Age | 41 | 0.546535483 | |
| MBA/LBA compared to Herbivores | Bronze Age | 34 | 0.996928173 | |
| LBA/EIA compared to Herbivores | Bronze to Iron Age Transition | 51 | 0.000002913 | * |
| IA compared to Herbivores | Iron Age | 41 | 0.000010538 | * |

**Tran-Urals**

| *T-test Slopes* | | *df* | *p-value* | |
|---|---|---|---|---|
| MBA compared to Herbivores | Bronze Age | 198 | 0.001097908 | * |
| LBA1 compared to Herbivores | Bronze Age | 87 | 0.239796290 | |
| LBA2 compared to Herbivores | Bronze Age | 65 | 0.032200333 | * |
| IA compared to Herbivores | Iron Age | 92 | 0.000000004 | * |

**C. Kazakhstan**

| *T-test Slopes* | | *df* | *p-value* | |
|---|---|---|---|---|
| LBA compared to Herbivores | Bronze Age | 109 | 0.652185983 | |
| FBA compared to Herbivores | Bronze to Iron Age Transition | 70 | 0.006710573 | * |

**Southeast Kazakhstan**

| *T-test Slopes* | | *df* | *p-value* |
|---|---|---|---|
| LBA1 compared to Herbivores | Bronze Age | 6 | 0.816914764 |
| LBA2 compared to Herbivores | Bronze Age | 13 | 0.083157221 |
| EIA compared to Herbivores | Iron Age | 51 | 0.369670734 |

**Southwest Siberia**

| *T-test Slopes* | | *df* | *p-value* | |
|---|---|---|---|---|
| NL/CL compared to Herbivores | Neolithic/Chalcolithic | 67 | 0.046207365 | * |
| EBA compared to Herbivores | Bronze Age | 31 | 0.033279060 | * |
| MBA compared to Herbivores | Bronze Age | 32 | 0.578966682 | |
| LBA compared to Herbivores | Bronze Age | 38 | 0.316442990 | |

**Minusinsk Basin**

| *T-test Slopes* | | *df* | *p-value* | |
|---|---|---|---|---|
| LN compared to Herbivores | Late Neolithic | 68 | 0.393182734 | |
| EBA compared to Herbivores | Bronze Age | 87 | 0.000644054 | * |
| LBA compared to Herbivores | Bronze Age | 84 | 0.018311235 | * |
| IA compared to Herbivores | Iron Age | 96 | 0.004208451 | * |

**N. Mongolia**

| *T-test Slopes* | | *df* | *p-value* | |
|---|---|---|---|---|
| LBA/EIA compared to Herbivores | Bronze to Iron Age Transition | 11 | 0.438387750 | |
| LIA compared to Herbivores | Late Iron Age | 30 | 0.000956529 | * |

**C. Mongolia**

| *T-test Slopes* | | *df* | *p-value* | |
|---|---|---|---|---|
| MBA compared to Herbivores | Bronze Age | 29 | 0.166165836 | |
| LBA/EIA compared to Herbivores | Bronze to Iron Age Transition | 26 | 0.412231823 | |
| LIA compared to Herbivores | Late Iron Age | 38 | 0.000935154 | * |

| CARBON | | | |
|---|---|---|---|
| **N. Caucasus** | **Difference** | **adj. p-value** | |
| MBA-MBA/LBA | 0.231201 | 0.872975 | |
| LBA/EIA-MBA/LBA | 4.494351 | 0.000000 | * |
| LBA/EIA-MBA | 4.263150 | 0.000000 | * |
| IA-MBA | 1.308910 | 0.000034 | * |
| IA-MBA/LBA | 1.540111 | 0.000054 | * |
| LBA/EIA-IA | 2.954240 | 0.000000 | * |
| **Trans-Urals** | **Difference** | **adj. p-value** | |
| LBA-LBA2 | 0.065224 | 0.995682 | |
| MBA-LBA2 | 0.304144 | 0.652608 | |
| MBA-LBA | 0.238920 | 0.257208 | |
| LBA2-IA | 1.149469 | 0.000323 | * |
| LBA-IA | 1.214693 | 0.000000 | * |
| MBA-IA | 1.453613 | 0.000000 | * |
| **C. Kazakhstan** | **Difference** | **adj. p-value** | |
| FBA-LBA | 0.427088 | 0.033429 | * |
| **SE Kazakhstan** | **Difference** | **adj. p-value** | |
| LBA2-LBA1 | 1.252161 | 0.136631 | |
| EIA-LBA2 | 0.700268 | 0.148132 | |
| EIA-LBA1 | 1.952429 | 0.003453 | * |
| **SW Siberia** | **Difference** | **adj. p-value** | |
| EBA-NLCL | 1.831607 | 0.003909 | * |
| MBA-NLCL | 1.590800 | 0.005973 | * |
| LBAEIA-NLCL | 2.447708 | 0.000000 | * |
| EBA-MBA | 0.240807 | 0.982764 | |
| LBAEIA-EBA | 0.616101 | 0.701619 | |
| LBAEIA-MBA | 0.856908 | 0.370713 | |
| **Minusinsk Basin** | **Difference** | **adj. p-value** | |
| EBA-LN | 0.478055 | 0.116139 | |
| LBA-LN | 3.147395 | 0.000000 | * |
| IA-LN | 2.872110 | 0.000000 | * |
| LBA-EBA | 2.669340 | 0.000000 | * |
| IA-EBA | 2.394054 | 0.000000 | * |
| LBA-IA | 0.275286 | 0.483199 | |
| **C. Mongolia** | **Difference** | **adj. p-value** | |
| MBA-LBA/EIA | 0.224917 | 0.770246 | |
| LIA-MBA | 0.867589 | 0.007260 | * |
| LIA-LBA/EIA | 1.092505 | 0.001800 | * |
| **N. Mongolia** | **Difference** | **adj. p-value** | |
| IA-LBA/EIA | 1.620677 | 0.000562 | * |

| NITROGEN | | | |
|---|---|---|---|
| **N. Caucasus** | **Difference** | **adj. p-value** | |
| MBA-MBA/LBA | 0.593338 | 0.043090 | * |
| LBA/EIA-MBA/LBA | 0.346415 | 0.329705 | |
| MBA-LBA/EIA | 0.246922 | 0.412598 | |
| MBA-IA | 1.178833 | 0.000000 | * |
| MBA/LBA-IA | 0.585495 | 0.055772 | |
| LBA/EIA-IA | 0.931911 | 0.000005 | * |
| **Trans-Urals** | **Difference** | **adj. p-value** | |
| LBA-LBA2 | 0.485674 | 0.751793 | |
| MBA-LBA2 | 0.605032 | 0.538335 | |
| MBA-LBA | 0.119359 | 0.950663 | |
| IA-LBA2 | 1.663601 | 0.003652 | * |
| IA-LBA | 1.177928 | 0.000204 | * |
| IA-MBA | 1.058569 | 0.000005 | * |
| **C. Kazakhstan** | **Difference** | **adj. p-value** | |
| FBA-LBA | 0.008228 | 0.982259 | |
| **SE Kazakhstan** | **Difference** | **adj. p-value** | |
| LBA2-LBA1 | 1.993808 | 0.031947 | * |
| LBA2-EIA | 1.800856 | 0.000378 | * |
| EIA-LBA1 | 0.192952 | 0.957366 | |
| **SW Siberia** | **Difference** | **adj. p-value** | |
| NLCL-EBA | 0.607357 | 0.530428 | |
| NLCL-MBA | 2.443612 | 0.000001 | * |
| LBAEIA-NLCL | 1.134757 | 0.001425 | * |
| EBA-MBA | 1.836255 | 0.011541 | * |
| LBAEIA-EBA | 1.742114 | 0.005014 | * |
| LBAEIA-MBA | 3.578368 | 0.000000 | * |
| **Minusinsk Basin** | **Difference** | **adj. p-value** | |
| LN-EBA | 0.184032 | 0.611923 | |
| LN-LBA | 0.424974 | 0.027870 | * |
| LN-IA | 0.028490 | 0.997397 | |
| EBA-LBA | 0.240942 | 0.311893 | |
| IA-EBA | 0.155542 | 0.652203 | |
| IA-LBA | 0.396484 | 0.019548 | * |
| **C. Mongolia** | **Difference** | **adj. p-value** | |
| LBA/EIA-MBA | 0.523005 | 0.541613 | |
| LIA-MBA | 0.414063 | 0.573183 | |
| LBA/EIA-LIA | 0.108942 | 0.967636 | |
| **N. Mongolia** | **Difference** | **adj. p-value** | |
| IA-LBA/EIA | 0.493648 | 0.179341 | |

| | $\delta^{13}C$ (‰) | | | | $\delta^{15}N$ (‰) | | | |
|---|---|---|---|---|---|---|---|---|
| **Northern Caucasus** | Min | Max | Mean | SD | Min | Max | Mean | SD |
| Humans EBA | -18.5 | -16.0 | -17.1 | 0.8 | 10.6 | 13.8 | 12.3 | 1.0 |
| Humans MBA | -17.8 | -15.4 | -17.0 | 0.7 | 9.0 | 15.8 | 12.8 | 2.1 |
| Humans MBA/LBA | -17.3 | -13.8 | -15.5 | 0.9 | 12.2 | 15.5 | 13.7 | 0.8 |
| Humans LBA/EIA | -20.0 | -13.2 | -17.0 | 1.5 | 5.1 | 12.6 | 9.4 | 2.2 |
| Humans IA | -18.5 | -14.4 | -17.3 | 1.3 | 9.1 | 11.9 | 10.1 | 0.6 |
| Livestock | -21.1 | -17.5 | -20.0 | 0.8 | 2.4 | 10.9 | 5.7 | 1.8 |
| **Trans-Urals** | Min | Max | Mean | SD | Min | Max | Mean | SD |
| Humans MBA | -19.7 | -16.8 | -18.5 | 0.6 | 9.5 | 15.6 | 12.3 | 1.4 |
| Humans LBA | -19.7 | -17.5 | -18.8 | 0.4 | 9.9 | 14.3 | 12.0 | 1.0 |
| Humans LBA2 | -20.3 | -18.4 | -19.0 | 0.7 | 10.5 | 11.9 | 11.4 | 0.5 |
| Humans IA | -22.8 | -17.5 | -19.8 | 1.3 | 10.8 | 14.9 | 12.6 | 0.9 |
| Livestock | -21.7 | -17.1 | -19.7 | 1.0 | 3.2 | 9.4 | 6.2 | 1.5 |
| Fish | -27.3 | -23.7 | -25.7 | 1.3 | 10.2 | 14.3 | 12.9 | 1.4 |
| **Central Kazakhstan** | Min | Max | Mean | SD | Min | Max | Mean | SD |
| Humans LBA | -19.2 | -15.7 | -18.4 | 0.6 | 11.4 | 17.7 | 13.3 | 1.2 |
| Humans FBA | -18.8 | -16.0 | -17.8 | 0.9 | 12.7 | 15.7 | 13.9 | 1.1 |
| Livestock | -20.6 | -17.9 | -19.2 | 0.7 | 4.8 | 13.3 | 7.5 | 1.6 |
| **Southeastern Kazakhstan** | Min | Max | Mean | SD | Min | Max | Mean | SD |
| Humans LBA1 | -19.0 | -16.3 | -17.8 | 1.2 | 9.7 | 12.8 | 10.7 | 1.4 |
| Humans LBA2 | -18.5 | -13.3 | -15.6 | 1.8 | 12.8 | 14.7 | 13.8 | 0.6 |
| Humans EIA | -17.3 | -12.9 | -15.4 | 1.0 | 8.8 | 17.2 | 12.1 | 1.5 |
| Livestock | -19.4 | -16.7 | -17.9 | 1.2 | 7.4 | 10.7 | 9.3 | 1.5 |
| **Southwest Siberia** | Min | Max | Mean | SD | Min | Max | Mean | SD |
| Humans LN/CL | -23.4 | -18.6 | -21.8 | 1.1 | 12.5 | 15.9 | 13.8 | 0.9 |
| Humans EBA | -21.0 | -18.3 | -19.7 | 1.2 | 12.4 | 13.2 | 12.8 | 0.4 |
| Humans MBA | -19.9 | -19.1 | -19.5 | 0.3 | 10.5 | 11.4 | 11.0 | 0.4 |
| Humans LBA | -20.2 | -18.6 | -19.5 | 0.6 | 12.5 | 15.9 | 14.5 | 1.0 |
| Livestock | -21.4 | -17.7 | -19.7 | 0.9 | 3.3 | 9.2 | 5.9 | 1.2 |
| Fish | -25.3 | -15.9 | -22.4 | 2.0 | 7.8 | 13.8 | 10.5 | 1.5 |
| **Minusinsk Basin** | Min | Max | Mean | SD | Min | Max | Mean | SD |
| Humans LN | -19.9 | -16.7 | -19.0 | 0.5 | 10.3 | 13.4 | 11.7 | 0.7 |
| Humans EBA | -19.5 | -15.9 | -18.6 | 0.5 | 10.5 | 12.5 | 11.5 | 0.5 |
| Humans LBA | -18.0 | -11.8 | -15.9 | 1.5 | 9.9 | 12.8 | 11.3 | 0.6 |
| Humans IA | -19.4 | -12.8 | -16.2 | 1.5 | 9.9 | 14.0 | 11.7 | 1.2 |
| Livestock | -21.4 | -18.3 | -20.1 | 1.0 | 3.8 | 9.4 | 5.7 | 1.6 |
| Fish | -26.7 | -23.1 | -24.4 | 1.5 | 8.8 | 14.9 | 11.5 | 2.4 |
| **Northern Mongolia** | Min | Max | Mean | SD | Min | Max | Mean | SD |
| Humans LBA/EIA | -18.0 | -17.2 | -17.7 | 0.4 | 11.1 | 12.1 | 11.5 | 0.4 |
| Humans LIA | -17.5 | -14.3 | -16.0 | 0.9 | 10.9 | 14.0 | 12.1 | 0.8 |
| Livestock | -19.4 | -17.7 | -18.4 | 0.5 | 7.2 | 8.7 | 8.1 | 0.5 |
| **Central Mongolia** | Min | Max | Mean | SD | Min | Max | Mean | SD |
| Humans MBA | -18.5 | -16.0 | -17.1 | 0.8 | 10.6 | 13.8 | 12.3 | 1.0 |
| Humans LBA/EIA | -17.8 | -15.4 | -17.0 | 0.7 | 9.0 | 15.8 | 12.8 | 2.1 |
| Humans LIA | -17.3 | -13.8 | -15.5 | 0.9 | 12.2 | 15.5 | 13.7 | 0.8 |
| Livestock | -20.0 | -13.2 | -17.0 | 1.5 | 5.1 | 12.6 | 9.4 | 2.2 |

| Original Sample Number | Site | Location | Period | $\delta^{13}C$ | $\delta^{15}N$ | Species (as identified in original publication) | Original Citation |
|---|---|---|---|---|---|---|---|
| KAB/1/12 | Kabardinka | N. Caucasus | MBA | -19.4 | 12.4 | Homo sapiens | Knipper et al. 2018 |
| KAB/1/13 | Kabardinka | N. Caucasus | MBA | -19.1 | 11.3 | Homo sapiens | Knipper et al. 2018 |
| KAB/2/2 | Kabardinka | N. Caucasus | MBA | -19.4 | 10.6 | Homo sapiens | Knipper et al. 2018 |
| KAB/1/3 | Kabardinka | N. Caucasus | MBA | -19.2 | 11.4 | Homo sapiens | Knipper et al. 2018 |
| KAB/1/1 | Kabardinka | N. Caucasus | MBA | -19.2 | 11.2 | Homo sapiens | Knipper et al. 2018 |
| KAB/1/4 | Kabardinka | N. Caucasus | MBA | -19.3 | 10.9 | Homo sapiens | Knipper et al. 2018 |
| KAB/1/6 | Kabardinka | N. Caucasus | MBA | -19.4 | 10.6 | Homo sapiens | Knipper et al. 2018 |
| KAB/1/9A | Kabardinka | N. Caucasus | MBA | -19.4 | 12.1 | Homo sapiens | Knipper et al. 2018 |
| KAB/1/9B | Kabardinka | N. Caucasus | MBA | -19.4 | 10.8 | Homo sapiens | Knipper et al. 2018 |
| KAB/1/10 | Kabardinka | N. Caucasus | MBA | -19.4 | 11.6 | Homo sapiens | Knipper et al. 2018 |
| KAB/2/1 | Kabardinka | N. Caucasus | MBA | -19.7 | 10.8 | Homo sapiens | Knipper et al. 2018 |
| KUD1/2 | Kudachurt | N. Caucasus | MBA | -19.0 | 11.8 | Homo sapiens | Hollund et al. 2010 |
| Zar1/1 | Zaragiž | N. Caucasus | MBA | -19.3 | 10.8 | Homo sapiens | Hollund et al. 2010 |
| Zar1/2 | Zaragiž | N. Caucasus | MBA | -19.4 | 10.8 | Homo sapiens | Hollund et al. 2010 |
| KUD 14/186, ind. I | Kudachurt K14 | N. Caucasus | MBA/LBA | -19.2 | 11.3 | Homo sapiens | Knipper et al. 2018 |
| KUD 14/186, ind. II | Kudachurt K14 | N. Caucasus | MBA/LBA | -19.5 | 10.8 | Homo sapiens | Knipper et al. 2018 |
| KUD 14/186, ind. III | Kudachurt K14 | N. Caucasus | MBA/LBA | -19.3 | 10.5 | Homo sapiens | Knipper et al. 2018 |
| KUD 14/186, ind. IV | Kudachurt K14 | N. Caucasus | MBA/LBA | -19.5 | 10.4 | Homo sapiens | Knipper et al. 2018 |
| KUD 14/186, ind. V | Kudachurt K14 | N. Caucasus | MBA/LBA | -19.1 | 10.9 | Homo sapiens | Knipper et al. 2018 |
| KUD 14/186, ind. VII | Kudachurt K14 | N. Caucasus | MBA/LBA | -19.3 | 10.4 | Homo sapiens | Knipper et al. 2018 |
| KUD 14/212 | Kudachurt K14 | N. Caucasus | MBA/LBA | -19.5 | 10.1 | Homo sapiens | Knipper et al. 2018 |
| 11 | Klin-Yar | N. Caucasus | LBA/EIA | -14.6 | 11.4 | Homo sapiens | Higham et al. 2010 |
| 172 | Klin-Yar | N. Caucasus | LBA/EIA | -15.5 | 11.2 | Homo sapiens | Higham et al. 2010 |
| 181 | Klin-Yar | N. Caucasus | LBA/EIA | -15.0 | 11.3 | Homo sapiens | Higham et al. 2010 |
| 187 | Klin-Yar | N. Caucasus | LBA/EIA | -15.8 | 10.9 | Homo sapiens | Higham et al. 2010 |
| 189 | Klin-Yar | N. Caucasus | LBA/EIA | -14.6 | 10.6 | Homo sapiens | Higham et al. 2010 |
| 190 | Klin-Yar | N. Caucasus | LBA/EIA | -16.0 | 10.2 | Homo sapiens | Higham et al. 2010 |

| | | | | | | | |
|---|---|---|---|---|---|---|---|
| 193 | Klin-Yar | N. Caucasus | LBA/EIA | -15.5 | 10.9 | Homo sapiens | Higham et al. 2010 |
| 206 | Klin-Yar | N. Caucasus | LBA/EIA | -14.4 | 11.1 | Homo sapiens | Higham et al. 2010 |
| 207 | Klin-Yar | N. Caucasus | LBA/EIA | -15.0 | 9.6 | Homo sapiens | Higham et al. 2010 |
| 208 | Klin-Yar | N. Caucasus | LBA/EIA | -15.0 | 10.6 | Homo sapiens | Higham et al. 2010 |
| 211 | Klin-Yar | N. Caucasus | LBA/EIA | -15.8 | 10.9 | Homo sapiens | Higham et al. 2010 |
| 214 | Klin-Yar | N. Caucasus | LBA/EIA | -14.8 | 10.5 | Homo sapiens | Higham et al. 2010 |
| 220 | Klin-Yar | N. Caucasus | LBA/EIA | -14.1 | 10.5 | Homo sapiens | Higham et al. 2010 |
| 236 | Klin-Yar | N. Caucasus | LBA/EIA | -15.4 | 11.1 | Homo sapiens | Higham et al. 2010 |
| 241 | Klin-Yar | N. Caucasus | LBA/EIA | -15.9 | 11.5 | Homo sapiens | Higham et al. 2010 |
| 277 | Klin-Yar | N. Caucasus | LBA/EIA | -14.3 | 10.9 | Homo sapiens | Higham et al. 2010 |
| 285 | Klin-Yar | N. Caucasus | LBA/EIA | -14.0 | 10.2 | Homo sapiens | Higham et al. 2010 |
| 315 | Klin-Yar | N. Caucasus | LBA/EIA | -14.6 | 11.0 | Homo sapiens | Higham et al. 2010 |
| 320 | Klin-Yar | N. Caucasus | LBA/EIA | -13.4 | 10.7 | Homo sapiens | Higham et al. 2010 |
| 323 | Klin-Yar | N. Caucasus | LBA/EIA | -14.9 | 10.6 | Homo sapiens | Higham et al. 2010 |
| 324 | Klin-Yar | N. Caucasus | LBA/EIA | -14.6 | 10.4 | Homo sapiens | Higham et al. 2010 |
| 349 | Klin-Yar | N. Caucasus | LBA/EIA | -14.3 | 10.9 | Homo sapiens | Higham et al. 2010 |
| 355 | Klin-Yar | N. Caucasus | LBA/EIA | -16.0 | 12.0 | Homo sapiens | Higham et al. 2010 |
| 377 | Klin-Yar | N. Caucasus | LBA/EIA | -14.6 | 10.8 | Homo sapiens | Higham et al. 2010 |
| 235 | Klin-Yar | N. Caucasus | IA | -18.2 | 10.2 | Homo sapiens | Higham et al. 2010 |
| 385 | Klin-Yar | N. Caucasus | IA | -18.3 | 10.2 | Homo sapiens | Higham et al. 2010 |
| 387 | Klin-Yar | N. Caucasus | IA | -18.5 | 10.0 | Homo sapiens | Higham et al. 2010 |
| 342B | Klin-Yar | N. Caucasus | IA | -17.4 | 9.8 | Homo sapiens | Higham et al. 2010 |
| 351A | Klin-Yar | N. Caucasus | IA | -15.6 | 9.1 | Homo sapiens | Higham et al. 2010 |
| 351B | Klin-Yar | N. Caucasus | IA | -16.6 | 10.2 | Homo sapiens | Higham et al. 2010 |
| 361A | Klin-Yar | N. Caucasus | IA | -17.5 | 10.5 | Homo sapiens | Higham et al. 2010 |
| 361B | Klin-Yar | N. Caucasus | IA | -15.7 | 10.0 | Homo sapiens | Higham et al. 2010 |
| 370 | Klin-Yar | N. Caucasus | IA | -18.1 | 9.6 | Homo sapiens | Higham et al. 2010 |
| 372 | Klin-Yar | N. Caucasus | IA | -18.3 | 9.9 | Homo sapiens | Higham et al. 2010 |

| | | | | | | | |
|---|---|---|---|---|---|---|---|
| 379 | Klin-Yar | N. Caucasus | IA | -18.4 | 10.1 | Homo sapiens | Higham et al. 2010 |
| 386/2 | Klin-Yar | N. Caucasus | IA | -18.2 | 10.2 | Homo sapiens | Higham et al. 2010 |
| Kurgan 1, Grab 9C | Kabardinka | N. Caucasus | MBA | -20.0 | 6.6 | sheep/goat | Knipper et al. in press |
| K3,G3 | Gorjačevodskij 2 | N. Caucasus | EBA | -19.5 | 7.4 | horse | Hollund et al. 2010 |
| K3,G26 | Inozcemevo | N. Caucasus | EBA | -19.3 | 5.9 | sheep/goat | Hollund et al. 2010 |
| K3,G8 | Inozcemevo | N. Caucasus | EBA | -19.0 | 5.5 | sheep | Hollund et al. 2010 |
| K3,G6 | Gorjačevodskij 2 | N. Caucasus | EBA | -20.0 | 5.1 | sheep | Hollund et al. 2010 |
| 367 | Klin-Yar | N. Caucasus | E. Med. | -21.1 | 2.4 | Horse | Higham et al. 2010 |
| 387 | Klin-Yar | N. Caucasus | IA | -19.8 | 10.9 | Ovicaprid | Higham et al. 2010 |
| 388 | Klin-Yar | N. Caucasus | IA | -21.1 | 4.3 | Horse | Higham et al. 2010 |
| Ex1 | Klin-Yar | N. Caucasus | | -20.5 | 6.0 | Horse | Higham et al. 2010 |
| Ex1S1 | Klin-Yar | N. Caucasus | | -20.3 | 6.8 | Ovicaprid | Higham et al. 2010 |
| Ex1S1 | Klin-Yar | N. Caucasus | | -19.9 | 7.7 | Ovicaprid | Higham et al. 2010 |
| 363 | Klin-Yar | N. Caucasus | E. Med. | -17.5 | 9.8 | Cow | Higham et al. 2010 |
| 384 | Klin-Yar | N. Caucasus | E. Med. | -20.4 | 6.2 | Cow | Higham et al. 2010 |
| 347 | Klin-Yar | N. Caucasus | E. Med. | -20.4 | 5.3 | Horse | Higham et al. 2010 |
| 357 | Klin-Yar | N. Caucasus | E. Med. | -20.1 | 5.1 | Horse | Higham et al. 2010 |
| 360 | Klin-Yar | N. Caucasus | E. Med. | -20.3 | 4.9 | Horse | Higham et al. 2010 |
| 363 | Klin-Yar | N. Caucasus | E. Med. | -20.8 | 3.1 | Horse | Higham et al. 2010 |
| 371 | Klin-Yar | N. Caucasus | E. Med. | -21.1 | 2.7 | Horse | Higham et al. 2010 |
| 384 | Klin-Yar | N. Caucasus | E. Med. | -20.1 | 7.0 | Ovicaprid | Higham et al. 2010 |
| 366 | Klin-Yar | N. Caucasus | LBA/EIA | -18.3 | 6.2 | Cow | Higham et al. 2010 |
| Koban | Klin-Yar | N. Caucasus | LBA/EIA | -19.0 | 7.8 | Cow | Higham et al. 2010 |
| Koban | Klin-Yar | N. Caucasus | LBA/EIA | -18.8 | 7.3 | Cow | Higham et al. 2010 |
| Koban | Klin-Yar | N. Caucasus | LBA/EIA | -19.7 | 6.5 | Ovicaprid | Higham et al. 2010 |
| Koban | Klin-Yar | N. Caucasus | LBA/EIA | -19.2 | 4.8 | Ovicaprid | Higham et al. 2010 |
| 370 | Klin-Yar | N. Caucasus | IA | -21.0 | 4.2 | Horse | Higham et al. 2010 |

| | | | | | | | |
|---|---|---|---|---|---|---|---|
| 372 | Klin-Yar | N. Caucasus | IA | -20.9 | 5.2 | Horse | Higham et al. 2010 |
| 372 | Klin-Yar | N. Caucasus | IA | -20.6 | 3.7 | Horse | Higham et al. 2010 |
| 378 | Klin-Yar | N. Caucasus | IA | -20.9 | 4.1 | Horse | Higham et al. 2010 |
| 360 | Klin-Yar | N. Caucasus | | -19.7 | 5.1 | Cow | Higham et al. 2010 |
| 361A | Klin-Yar | N. Caucasus | | -18.8 | 7.5 | Cow | Higham et al. 2010 |
| 341 | Klin-Yar | N. Caucasus | | -20.2 | 5.7 | Horse | Higham et al. 2010 |
| 357 | Klin-Yar | N. Caucasus | | -19.6 | 6.0 | Horse | Higham et al. 2010 |
| 360 | Klin-Yar | N. Caucasus | | -20.3 | 4.9 | Horse | Higham et al. 2010 |
| 361B | Klin-Yar | N. Caucasus | | -20.2 | 3.8 | Horse | Higham et al. 2010 |
| 363B | Klin-Yar | N. Caucasus | | -20.1 | 4.3 | Horse | Higham et al. 2010 |
| 378 | Klin-Yar | N. Caucasus | | -20.4 | 4.1 | Horse | Higham et al. 2010 |
| BES 8 | Bestamak | Trans-Urals | MBA | -18.8 | 10.7 | Homo sapiens | Privat 2004 |
| BES 16 | Bestamak | Trans-Urals | MBA | -19.2 | 10.5 | Homo sapiens | Privat 2004 |
| BES 13 | Bestamak | Trans-Urals | MBA | -18.0 | 13.6 | Homo sapiens | Privat 2004 |
| BES 11 | Bestamak | Trans-Urals | MBA | -17.8 | 12.9 | Homo sapiens | Privat 2004 |
| BES 17 | Bestamak | Trans-Urals | MBA | -18.7 | 11.2 | Homo sapiens | Privat 2004 |
| BES 9 | Bestamak | Trans-Urals | MBA | -19.1 | 10.5 | Homo sapiens | Privat 2004 |
| BES 15 | Bestamak | Trans-Urals | MBA | -18.7 | 11.0 | Homo sapiens | Privat 2004 |
| BES 14 | Bestamak | Trans-Urals | MBA | -18.9 | 10.6 | Homo sapiens | Privat 2004 |
| BES 12 | Bestamak | Trans-Urals | MBA | -18.9 | 11.0 | Homo sapiens | Privat 2004 |
| BES 18 | Bestamak | Trans-Urals | MBA | -19.3 | 10.5 | Homo sapiens | Privat 2004 |
| BES 6 | Bestamak | Trans-Urals | MBA | -19.4 | 10.8 | Homo sapiens | Privat 2004 |
| BES 10 | Bestamak | Trans-Urals | MBA | -18.4 | 12.3 | Homo sapiens | Privat 2004 |
| BES 19 | Bestamak | Trans-Urals | MBA | -19.0 | 10.8 | Homo sapiens | Privat 2004 |
| BES 7 | Bestamak | Trans-Urals | MBA | -19.0 | 11.0 | Homo sapiens | Privat 2004 |
| B 3501 | Bestamak | Trans-Urals | MBA | -19.4 | 11.6 | Homo sapiens | Privat 2004 |
| B 3507 | Bestamak | Trans-Urals | MBA | -19.2 | 11.6 | Homo sapiens | Privat 2004 |

| | | | | | | | |
|---|---|---|---|---|---|---|---|
| B 3508 | Bestamak | Trans-Urals | MBA | -19.2 | 11.1 | Homo sapiens | Privat 2004 |
| B 3512 | Bestamak | Trans-Urals | MBA | -19.4 | 11.4 | Homo sapiens | Privat 2004 |
| B 3513 | Bestamak | Trans-Urals | MBA | -19.4 | 12.1 | Homo sapiens | Privat 2004 |
| B 3515 | Bestamak | Trans-Urals | MBA | -19.6 | 11.3 | Homo sapiens | Privat 2004 |
| B 3518 | Bestamak | Trans-Urals | MBA | -18.8 | 11.9 | Homo sapiens | Privat 2004 |
| B 3520 | Bestamak | Trans-Urals | MBA | -19.2 | 11.3 | Homo sapiens | Privat 2004 |
| B 3523 | Bestamak | Trans-Urals | MBA | -18.7 | 12.6 | Homo sapiens | Privat 2004 |
| B 3531 | Bestamak | Trans-Urals | MBA | -18.6 | 12.5 | Homo sapiens | Privat 2004 |
| B 3532 | Bestamak | Trans-Urals | MBA | -19.5 | 11.0 | Homo sapiens | Privat 2004 |
| B 3534 | Bestamak | Trans-Urals | MBA | -19.0 | 9.5 | Homo sapiens | Privat 2004 |
| B 3538 | Bestamak | Trans-Urals | MBA | -19.2 | 11.1 | Homo sapiens | Privat 2004 |
| B 3540 | Bestamak | Trans-Urals | MBA | -17.6 | 14.1 | Homo sapiens | Privat 2004 |
| B 3542 | Bestamak | Trans-Urals | MBA | -19.0 | 11.1 | Homo sapiens | Privat 2004 |
| B 3545 | Bestamak | Trans-Urals | MBA | -18.7 | 11.5 | Homo sapiens | Privat 2004 |
| B 3547 | Bestamak | Trans-Urals | MBA | -19.3 | 12.1 | Homo sapiens | Privat 2004 |
| B 3550 | Bestamak | Trans-Urals | MBA | -19.4 | 12.0 | Homo sapiens | Privat 2004 |
| B 3558 | Bestamak | Trans-Urals | MBA | -18.3 | 14.0 | Homo sapiens | Privat 2004 |
| B 3566 | Bestamak | Trans-Urals | MBA | -18.7 | 11.9 | Homo sapiens | Privat 2004 |
| B 3575 | Bestamak | Trans-Urals | MBA | -18.9 | 11.2 | Homo sapiens | Privat 2004 |
| BKAR 4 | Bolshekaragansky | Trans-Urals | MBA | -19.3 | 10.5 | Homo sapiens | Privat 2004 |
| BKAR 12 | Bolshekaragansky | Trans-Urals | MBA | -18.9 | 11.7 | Homo sapiens | Privat 2004 |
| BKAR 14 | Bolshekaragansky | Trans-Urals | MBA | -19.2 | 10.8 | Homo sapiens | Privat 2004 |
| BKAR 6 | Bolshekaragansky | Trans-Urals | MBA | -18.3 | 11.6 | Homo sapiens | Privat 2004 |
| BKAR 13 | Bolshekaragansky | Trans-Urals | MBA | -19.3 | 11.2 | Homo sapiens | Privat 2004 |
| BKAR 7 | Bolshekaragansky | Trans-Urals | MBA | -18.9 | 10.5 | Homo sapiens | Privat 2004 |
| BKAR 8 | Bolshekaragansky | Trans-Urals | MBA | -18.2 | 11.2 | Homo sapiens | Privat 2004 |
| BKAR 1 | Bolshekaragansky | Trans-Urals | MBA | -18.0 | 11.8 | Homo sapiens | Privat 2004 |

| | | | | | | | |
|---|---|---|---|---|---|---|---|
| BKAR 9 | Bolshekaragansky | Trans-Urals | MBA | -18.3 | 11.5 | Homo sapiens | Privat 2004 |
| BKAR 3 | Bolshekaragansky | Trans-Urals | MBA | -18.9 | 10.3 | Homo sapiens | Privat 2004 |
| BKAR 11 | Bolshekaragansky | Trans-Urals | MBA | -19.1 | 10.6 | Homo sapiens | Privat 2004 |
| BKAR 10 | Bolshekaragansky | Trans-Urals | MBA | -19.2 | 9.5 | Homo sapiens | Privat 2004 |
| BKAR 2 | Bolshekaragansky | Trans-Urals | MBA | -19.1 | 9.7 | Homo sapiens | Privat 2004 |
| BKAR 5 | Bolshekaragansky | Trans-Urals | MBA | -19.2 | 10.3 | Homo sapiens | Privat 2004 |
| KAM 31 | Kamenni Ambar | Trans-Urals | MBA | -18.3 | 12.0 | Homo sapiens | Privat 2004 |
| KAM 19 | Kamenni Ambar V | Trans-Urals | MBA | -17.8 | 13.3 | Homo sapiens | Privat 2004 |
| KAM 37 | Kamenni Ambar V | Trans-Urals | MBA | -17.9 | 12.7 | Homo sapiens | Privat 2004 |
| KAM 28 | Kamenni Ambar V | Trans-Urals | MBA | -18.9 | 11.7 | Homo sapiens | Privat 2004 |
| KAM 22 | Kamenni Ambar V | Trans-Urals | MBA | -18.5 | 11.5 | Homo sapiens | Privat 2004 |
| KAM 32 | Kamenni Ambar V | Trans-Urals | MBA | -17.6 | 13.4 | Homo sapiens | Privat 2004 |
| KAM 24 | Kamenni Ambar V | Trans-Urals | MBA | -18.0 | 12.8 | Homo sapiens | Privat 2004 |
| KAM 36 | Kamenni Ambar V | Trans-Urals | MBA | -17.5 | 14.6 | Homo sapiens | Privat 2004 |
| KAM 38 | Kamenni Ambar V | Trans-Urals | MBA | -18.3 | 12.7 | Homo sapiens | Privat 2004 |
| KAM 26 | Kamenni Ambar V | Trans-Urals | MBA | -19.3 | 11.3 | Homo sapiens | Privat 2004 |
| KAM 34 | Kamenni Ambar V | Trans-Urals | MBA | -17.1 | 14.8 | Homo sapiens | Privat 2004 |
| KAM 25 | Kamenni Ambar V | Trans-Urals | MBA | -18.3 | 13.9 | Homo sapiens | Privat 2004 |
| KAM 21 | Kamenni Ambar V | Trans-Urals | MBA | -18.4 | 12.3 | Homo sapiens | Privat 2004 |
| KAM 33 | Kamenni Ambar V | Trans-Urals | MBA | -17.6 | 13.4 | Homo sapiens | Privat 2004 |
| KAM 35 | Kamenni Ambar V | Trans-Urals | MBA | -17.7 | 14.2 | Homo sapiens | Privat 2004 |
| KAM 29 | Kamenni Ambar V | Trans-Urals | MBA | -18.0 | 11.9 | Homo sapiens | Privat 2004 |
| KAM 30 | Kamenni Ambar V | Trans-Urals | MBA | -18.8 | 11.7 | Homo sapiens | Privat 2004 |
| KAM 23 | Kamenni Ambar V | Trans-Urals | MBA | -17.9 | 12.0 | Homo sapiens | Privat 2004 |
| KAM 27 | Kamenni Ambar V | Trans-Urals | MBA | -17.8 | 13.7 | Homo sapiens | Privat 2004 |
| KA-5 A1010 | Kamennyi Ambar | Trans-Urals | MBA | -17.8 | 13.3 | Homo sapiens | Hanks et al. 2018 |
| KA-5 A0946 | Kamennyi Ambar | Trans-Urals | MBA | -18.6 | 12.9 | Homo sapiens | Hanks et al. 2018 |

| | | | | | | | |
|---|---|---|---|---|---|---|---|
| KA-5 A0954 | Kamennyi Ambar | Trans-Urals | MBA | -18.0 | 15.6 | Homo sapiens | Hanks et al. 2018 |
| KA-5 A0962 | Kamennyi Ambar | Trans-Urals | MBA | -17.9 | 15.5 | Homo sapiens | Hanks et al. 2018 |
| KA-5 A1000 | Kamennyi Ambar | Trans-Urals | MBA | -17.8 | 14.7 | Homo sapiens | Hanks et al. 2018 |
| KA-5 A1002 | Kamennyi Ambar | Trans-Urals | MBA | -17.7 | 14.9 | Homo sapiens | Hanks et al. 2018 |
| KA-5 A1015 | Kamennyi Ambar | Trans-Urals | MBA | -19.1 | 11.2 | Homo sapiens | Hanks et al. 2018 |
| KA-5 A0942 | Kamennyi Ambar | Trans-Urals | MBA | -18.3 | 12.7 | Homo sapiens | Hanks et al. 2018 |
| KA-5 A0949 | Kamennyi Ambar | Trans-Urals | MBA | -17.7 | 13.4 | Homo sapiens | Hanks et al. 2018 |
| KA-5 A0952 | Kamennyi Ambar | Trans-Urals | MBA | -18.2 | 13.7 | Homo sapiens | Hanks et al. 2018 |
| KA-5 A0953 | Kamennyi Ambar | Trans-Urals | MBA | -17.9 | 13.9 | Homo sapiens | Hanks et al. 2018 |
| KA-5 A0955 | Kamennyi Ambar | Trans-Urals | MBA | -17.8 | 14.4 | Homo sapiens | Hanks et al. 2018 |
| KA-5 A0963 | Kamennyi Ambar | Trans-Urals | MBA | -17.6 | 15.6 | Homo sapiens | Hanks et al. 2018 |
| KA-5 A0965 | Kamennyi Ambar | Trans-Urals | MBA | -17.5 | 14.6 | Homo sapiens | Hanks et al. 2018 |
| KA-5 A0966 | Kamennyi Ambar | Trans-Urals | MBA | -17.2 | 15.6 | Homo sapiens | Hanks et al. 2018 |
| KA-5 A0968 | Kamennyi Ambar | Trans-Urals | MBA | -17.9 | 13.4 | Homo sapiens | Hanks et al. 2018 |
| KA-5 A0969 | Kamennyi Ambar | Trans-Urals | MBA | -17.9 | 12.1 | Homo sapiens | Hanks et al. 2018 |
| KA-5 A0984 | Kamennyi Ambar | Trans-Urals | MBA | -17.4 | 14.4 | Homo sapiens | Hanks et al. 2018 |
| KA-5 A1006 | Kamennyi Ambar | Trans-Urals | MBA | -17.7 | 14.5 | Homo sapiens | Hanks et al. 2018 |
| KA-5 A0940 | Kamennyi Ambar | Trans-Urals | MBA | -18.3 | 12.9 | Homo sapiens | Hanks et al. 2018 |
| KA-5 A0945 | Kamennyi Ambar | Trans-Urals | MBA | -18.2 | 12.2 | Homo sapiens | Hanks et al. 2018 |
| KA-5 A0950 | Kamennyi Ambar | Trans-Urals | MBA | -17.9 | 13.6 | Homo sapiens | Hanks et al. 2018 |
| KA-5 A0951 | Kamennyi Ambar | Trans-Urals | MBA | -18.0 | 13.9 | Homo sapiens | Hanks et al. 2018 |
| KA-5 A0960 | Kamennyi Ambar | Trans-Urals | MBA | -18.0 | 12.9 | Homo sapiens | Hanks et al. 2018 |
| KA-5 A0964 | Kamennyi Ambar | Trans-Urals | MBA | -18.0 | 13.0 | Homo sapiens | Hanks et al. 2018 |
| KA-5 A0970 | Kamennyi Ambar | Trans-Urals | MBA | -18.4 | 11.2 | Homo sapiens | Hanks et al. 2018 |
| KA-5 A0975 | Kamennyi Ambar | Trans-Urals | MBA | -17.9 | 12.8 | Homo sapiens | Hanks et al. 2018 |
| KA-5 A0977 | Kamennyi Ambar | Trans-Urals | MBA | -16.8 | 15.0 | Homo sapiens | Hanks et al. 2018 |
| KA-5 A0980 | Kamennyi Ambar | Trans-Urals | MBA | -18.1 | 12.4 | Homo sapiens | Hanks et al. 2018 |

| | | | | | | | |
|---|---|---|---|---|---|---|---|
| KA-5 A0983 | Kamennyi Ambar | Trans-Urals | MBA | -18.0 | 13.6 | Homo sapiens | Hanks et al. 2018 |
| KA-5 A0990 | Kamennyi Ambar | Trans-Urals | MBA | -18.3 | 12.0 | Homo sapiens | Hanks et al. 2018 |
| KA-5 A0991 | Kamennyi Ambar | Trans-Urals | MBA | -18.3 | 12.2 | Homo sapiens | Hanks et al. 2018 |
| KA-5 A0992 | Kamennyi Ambar | Trans-Urals | MBA | -18.2 | 12.1 | Homo sapiens | Hanks et al. 2018 |
| KA-5 A0993 | Kamennyi Ambar | Trans-Urals | MBA | -18.2 | 12.6 | Homo sapiens | Hanks et al. 2018 |
| KA-5 A0997 | Kamennyi Ambar | Trans-Urals | MBA | -18.0 | 12.9 | Homo sapiens | Hanks et al. 2018 |
| KA-5 A1005 | Kamennyi Ambar | Trans-Urals | MBA | -17.7 | 13.4 | Homo sapiens | Hanks et al. 2018 |
| KA-5 A1008 | Kamennyi Ambar | Trans-Urals | MBA | -18.2 | 14.2 | Homo sapiens | Hanks et al. 2018 |
| KA-5 A1009 | Kamennyi Ambar | Trans-Urals | MBA | -18.3 | 11.7 | Homo sapiens | Hanks et al. 2018 |
| KA-5 A1013 | Kamennyi Ambar | Trans-Urals | MBA | -19.0 | 10.6 | Homo sapiens | Hanks et al. 2018 |
| KA-5 A1016 | Kamennyi Ambar | Trans-Urals | MBA | -19.0 | 10.9 | Homo sapiens | Hanks et al. 2018 |
| KA-5 A1017 | Kamennyi Ambar | Trans-Urals | MBA | -19.3 | 11.3 | Homo sapiens | Hanks et al. 2018 |
| KA-5 A1024 | Kamennyi Ambar | Trans-Urals | MBA | -17.8 | 12.9 | Homo sapiens | Hanks et al. 2018 |
| KA-5 A0976 | Kamennyi Ambar | Trans-Urals | MBA | -18.0 | 12.6 | Homo sapiens | Hanks et al. 2018 |
| KA-5 A0978 | Kamennyi Ambar | Trans-Urals | MBA | -17.5 | 14.5 | Homo sapiens | Hanks et al. 2018 |
| KA-5 A0982 | Kamennyi Ambar | Trans-Urals | MBA | -17.8 | 13.6 | Homo sapiens | Hanks et al. 2018 |
| KA-5 A0992 | Kamennyi Ambar | Trans-Urals | MBA | -18.2 | 12.1 | Homo sapiens | Hanks et al. 2018 |
| KA-5 A0996 | Kamennyi Ambar | Trans-Urals | MBA | -18.5 | 11.5 | Homo sapiens | Hanks et al. 2018 |
| KA-5 A1012 | Kamennyi Ambar | Trans-Urals | MBA | -18.8 | 11.2 | Homo sapiens | Hanks et al. 2018 |
| KA-5 A1018 | Kamennyi Ambar | Trans-Urals | MBA | -19.1 | 11.1 | Homo sapiens | Hanks et al. 2018 |
| KA-5 A0939 | Kamennyi Ambar | Trans-Urals | MBA | -18.2 | 12.2 | Homo sapiens | Hanks et al. 2018 |
| KA-5 A0948 | Kamennyi Ambar | Trans-Urals | MBA | -19.0 | 11.4 | Homo sapiens | Hanks et al. 2018 |
| KA-5 A0957 | Kamennyi Ambar | Trans-Urals | MBA | -17.6 | 13.9 | Homo sapiens | Hanks et al. 2018 |
| KA-5 A0986 | Kamennyi Ambar | Trans-Urals | MBA | -19.4 | 14.8 | Homo sapiens | Hanks et al. 2018 |
| KA-5 A0988 | Kamennyi Ambar | Trans-Urals | MBA | -18.0 | 13.8 | Homo sapiens | Hanks et al. 2018 |
| KA-5 A1014 | Kamennyi Ambar | Trans-Urals | MBA | -18.3 | 12.8 | Homo sapiens | Hanks et al. 2018 |
| KA-5 A0937 | Kamennyi Ambar | Trans-Urals | MBA | -17.9 | 14.2 | Homo sapiens | Hanks et al. 2018 |

| | | | | | | | |
|---|---|---|---|---|---|---|---|
| KA-5 A0971 | Kamennyi Ambar | Trans-Urals | MBA | -19.1 | 11.6 | Homo sapiens | Hanks et al. 2018 |
| KA-5 A0981 | Kamennyi Ambar | Trans-Urals | MBA | -18.4 | 12.2 | Homo sapiens | Hanks et al. 2018 |
| KA-5 A0985 | Kamennyi Ambar | Trans-Urals | MBA | -19.7 | 10.9 | Homo sapiens | Hanks et al. 2018 |
| KA-5 A0989 | Kamennyi Ambar | Trans-Urals | MBA | -18.5 | 12.5 | Homo sapiens | Hanks et al. 2018 |
| KA-5 A0967 | Kamennyi Ambar | Trans-Urals | MBA | -19.0 | 12.3 | Homo sapiens | Hanks et al. 2018 |
| KA-5 A0972 | Kamennyi Ambar | Trans-Urals | MBA | -18.3 | 15.2 | Homo sapiens | Hanks et al. 2018 |
| KA-5 A0973 | Kamennyi Ambar | Trans-Urals | MBA | -17.9 | 14.1 | Homo sapiens | Hanks et al. 2018 |
| KA-5 A0979 | Kamennyi Ambar | Trans-Urals | MBA | -18.2 | 12.3 | Homo sapiens | Hanks et al. 2018 |
| KA-5 A0994 | Kamennyi Ambar | Trans-Urals | MBA | -19.0 | 10.5 | Homo sapiens | Hanks et al. 2018 |
| KA-5 A0995 | Kamennyi Ambar | Trans-Urals | MBA | -18.4 | 12.6 | Homo sapiens | Hanks et al. 2018 |
| KA-5 A1011 | Kamennyi Ambar | Trans-Urals | MBA | -18.2 | 12.3 | Homo sapiens | Hanks et al. 2018 |
| KA-5 A0941 | Kamennyi Ambar | Trans-Urals | MBA | -18.2 | 13.5 | Homo sapiens | Hanks et al. 2018 |
| KA-5 A0947 | Kamennyi Ambar | Trans-Urals | MBA | -19.4 | 10.6 | Homo sapiens | Hanks et al. 2018 |
| KA-5 A0956 | Kamennyi Ambar | Trans-Urals | MBA | -17.9 | 14.5 | Homo sapiens | Hanks et al. 2018 |
| KA-5 A0987 | Kamennyi Ambar | Trans-Urals | MBA | -17.5 | 13.2 | Homo sapiens | Hanks et al. 2018 |
| KZ18 | Novoilinovka | Trans-Urals | MBA | -18.6 | 11.4 | Homo sapiens | Motuzaite Matuzeviciute et al. 2016 |
| KZ19 | Novoilinovka | Trans-Urals | MBA | -19.1 | 10.8 | Homo sapiens | Motuzaite Matuzeviciute et al. 2016 |
| KZ20 | Novoilinovka | Trans-Urals | MBA | -19.1 | 11.4 | Homo sapiens | Motuzaite Matuzeviciute et al. 2016 |
| KZ21 | Novoilinovka | Trans-Urals | MBA | -18.7 | 11.2 | Homo sapiens | Motuzaite Matuzeviciute et al. 2016 |
| KZ22 | Novoilinovka | Trans-Urals | MBA | -18.9 | 11.0 | Homo sapiens | Motuzaite Matuzeviciute et al. 2016 |
| KZ23 | Novoilinovka | Trans-Urals | MBA | -18.9 | 11.1 | Homo sapiens | Motuzaite Matuzeviciute et al. 2016 |
| KZ24 | Novoilinovka | Trans-Urals | MBA | -18.8 | 11.2 | Homo sapiens | Motuzaite Matuzeviciute et al. 2016 |
| KZ25 | Novoilinovka | Trans-Urals | MBA | -19.2 | 11.3 | Homo sapiens | Motuzaite Matuzeviciute et al. 2016 |
| L 3001 | Lisakovsk | Trans-Urals | LBA | -18.8 | 11.5 | Homo sapiens | Ventresca Miller et al. 2014 |
| L 3004 | Lisakovsk | Trans-Urals | LBA | -19.0 | 11.6 | Homo sapiens | Ventresca Miller et al. 2014 |
| L 3013 | Lisakovsk | Trans-Urals | LBA | -18.9 | 12.5 | Homo sapiens | Ventresca Miller et al. 2014 |
| L3016 | Lisakovsk | Trans-Urals | LBA | -18.9 | 9.9 | Homo sapiens | Ventresca Miller et al. 2014 |

| | | | | | | | |
|---|---|---|---|---|---|---|---|
| L 3036 | Lisakovsk | Trans-Urals | LBA | -18.5 | 12.0 | Homo sapiens | Ventresca Miller et al. 2014 |
| L 3070 | Lisakovsk | Trans-Urals | LBA | -18.7 | 12.0 | Homo sapiens | Ventresca Miller et al. 2014 |
| L 3071 | Lisakovsk | Trans-Urals | LBA | -18.6 | 13.9 | Homo sapiens | Ventresca Miller et al. 2014 |
| L 3081 | Lisakovsk | Trans-Urals | LBA | -18.9 | 12.1 | Homo sapiens | Ventresca Miller et al. 2014 |
| L 3093 | Lisakovsk | Trans-Urals | LBA | -19.4 | 11.5 | Homo sapiens | Ventresca Miller et al. 2014 |
| L 3102 | Lisakovsk | Trans-Urals | LBA | -18.7 | 12.4 | Homo sapiens | Ventresca Miller et al. 2014 |
| L3105 | Lisakovsk | Trans-Urals | LBA | -19.0 | 12.0 | Homo sapiens | Ventresca Miller et al. 2014 |
| L 3110 | Lisakovsk | Trans-Urals | LBA | -19.3 | 11.2 | Homo sapiens | Ventresca Miller et al. 2014 |
| L 3112 | Lisakovsk | Trans-Urals | LBA | -18.8 | 13.5 | Homo sapiens | Ventresca Miller et al. 2014 |
| L 3130 | Lisakovsk | Trans-Urals | LBA | -18.9 | 12.8 | Homo sapiens | Ventresca Miller et al. 2014 |
| L 3137 | Lisakovsk | Trans-Urals | LBA | -17.5 | 14.3 | Homo sapiens | Ventresca Miller et al. 2014 |
| L 3139 | Lisakovsk | Trans-Urals | LBA | -19.0 | 12.4 | Homo sapiens | Ventresca Miller et al. 2014 |
| L3142 | Lisakovsk | Trans-Urals | LBA | -19.0 | 11.2 | Homo sapiens | Ventresca Miller et al. 2014 |
| L 3150 | Lisakovsk | Trans-Urals | LBA | -19.0 | 11.9 | Homo sapiens | Ventresca Miller et al. 2014 |
| L 3155 | Lisakovsk | Trans-Urals | LBA | -17.6 | 13.1 | Homo sapiens | Ventresca Miller et al. 2014 |
| L 3160 | Lisakovsk | Trans-Urals | LBA | -18.5 | 12.5 | Homo sapiens | Ventresca Miller et al. 2014 |
| L 3161 | Lisakovsk | Trans-Urals | LBA | -18.6 | 10.9 | Homo sapiens | Ventresca Miller et al. 2014 |
| L 3165 | Lisakovsk | Trans-Urals | LBA | -18.8 | 11.3 | Homo sapiens | Ventresca Miller et al. 2014 |
| L 3167 | Lisakovsk | Trans-Urals | LBA | -18.3 | 12.2 | Homo sapiens | Ventresca Miller et al. 2014 |
| L 3168 | Lisakovsk | Trans-Urals | LBA | -19.0 | 11.7 | Homo sapiens | Ventresca Miller et al. 2014 |
| L 3170 | Lisakovsk | Trans-Urals | LBA | -19.1 | 12.0 | Homo sapiens | Ventresca Miller et al. 2014 |
| L 3173 | Lisakovsk | Trans-Urals | LBA | -18.8 | 12.0 | Homo sapiens | Ventresca Miller et al. 2014 |
| L 3178 | Lisakovsk | Trans-Urals | LBA | -19.7 | 10.0 | Homo sapiens | Ventresca Miller et al. 2014 |
| L 3184 | Lisakovsk | Trans-Urals | LBA | -18.7 | 11.9 | Homo sapiens | Ventresca Miller et al. 2014 |
| ISI 4 | Isiney I | Trans-Urals | LBA2 | -18.4 | 11.9 | Homo sapiens | Privat 2004 |
| ISI 5 | Isiney I | Trans-Urals | LBA2 | -20.3 | 11.4 | Homo sapiens | Privat 2004 |
| ISI 3 | Isiney I | Trans-Urals | LBA2 | -19.1 | 10.5 | Homo sapiens | Privat 2004 |

| | | | | | | | |
|---|---|---|---|---|---|---|---|
| ISI 1 | Isiney I | Trans-Urals | LBA2 | -18.8 | 11.6 | Homo sapiens | Privat 2004 |
| ISI 6 | Isiney I | Trans-Urals | LBA2 | -18.8 | 11.6 | Homo sapiens | Privat 2004 |
| ISI 2 | Isiney I | Trans-Urals | LBA2 | -18.8 | 11.3 | Homo sapiens | Privat 2004 |
| GAY 1 | Gayovsky I | Trans-Urals | IA | -20.7 | 12.7 | Homo sapiens | Privat 2004 |
| GAY 3 | Gayovsky I | Trans-Urals | IA | -21.5 | 12.8 | Homo sapiens | Privat 2004 |
| GAY 4 | Gayovsky I | Trans-Urals | IA | -20.0 | 11.4 | Homo sapiens | Privat 2004 |
| GAY 5 | Gayovsky I | Trans-Urals | IA | -20.6 | 12.4 | Homo sapiens | Privat 2004 |
| GAY 6 | Gayovsky I | Trans-Urals | IA | -20.3 | 11.0 | Homo sapiens | Privat 2004 |
| GAY 7 | Gayovsky I | Trans-Urals | IA | -20.3 | 12.0 | Homo sapiens | Privat 2004 |
| KUR 10 | Kurtuguz I | Trans-Urals | IA | -18.1 | 11.7 | Homo sapiens | Privat 2004 |
| KUR 4 | Kurtuguz I | Trans-Urals | IA | -17.9 | 12.9 | Homo sapiens | Privat 2004 |
| KUR 5 | Kurtuguz I | Trans-Urals | IA | -19.8 | 12.7 | Homo sapiens | Privat 2004 |
| KUR 6 | Kurtuguz I | Trans-Urals | IA | -17.5 | 12.6 | Homo sapiens | Privat 2004 |
| KUR 7 | Kurtuguz I | Trans-Urals | IA | -21.6 | 13.0 | Homo sapiens | Privat 2004 |
| KUR 8 | Kurtuguz I | Trans-Urals | IA | -21.4 | 13.0 | Homo sapiens | Privat 2004 |
| KUR 9 | Kurtuguz I | Trans-Urals | IA | -20.3 | 14.3 | Homo sapiens | Privat 2004 |
| MUR 1 | Murzino I | Trans-Urals | IA | -20.5 | 12.5 | Homo sapiens | Privat 2004 |
| MUR 2 | Murzino I | Trans-Urals | IA | -21.1 | 12.1 | Homo sapiens | Privat 2004 |
| MUR 4 | Murzino I | Trans-Urals | IA | -20.4 | 13.0 | Homo sapiens | Privat 2004 |
| MUR 5 | Murzino I | Trans-Urals | IA | -22.8 | 13.4 | Homo sapiens | Privat 2004 |
| MUR 6 | Murzino I | Trans-Urals | IA | -20.9 | 14.9 | Homo sapiens | Privat 2004 |
| MUR 7 | Murzino I | Trans-Urals | IA | -21.0 | 12.4 | Homo sapiens | Privat 2004 |
| POB 1 | Pobyeda | Trans-Urals | IA | -18.0 | 13.0 | Homo sapiens | Privat 2004 |
| POB 2 | Pobyeda | Trans-Urals | IA | -17.9 | 13.8 | Homo sapiens | Privat 2004 |
| POB 4 | Pobyeda | Trans-Urals | IA | -18.3 | 12.6 | Homo sapiens | Privat 2004 |
| POB 5 | Pobyeda | Trans-Urals | IA | -18.4 | 13.0 | Homo sapiens | Privat 2004 |
| POB 6 | Pobyeda | Trans-Urals | IA | -18.7 | 13.1 | Homo sapiens | Privat 2004 |

| | | | | | | | |
|---|---|---|---|---|---|---|---|
| POB 7 | Pobyeda | Trans-Urals | IA | -18.8 | 12.4 | Homo sapiens | Privat 2004 |
| POB 8 | Pobyeda | Trans-Urals | IA | -18.8 | 12.6 | Homo sapiens | Privat 2004 |
| SHA 1 | Shaidurikha | Trans-Urals | IA | -19.9 | 11.9 | Homo sapiens | Privat 2004 |
| SHA 2 | Shaidurikha | Trans-Urals | IA | -19.9 | 12.8 | Homo sapiens | Privat 2004 |
| SHA 3 | Shaidurikha | Trans-Urals | IA | -20.6 | 12.0 | Homo sapiens | Privat 2004 |
| SHA 4 | Shaidurikha | Trans-Urals | IA | -19.1 | 12.1 | Homo sapiens | Privat 2004 |
| SHA 5 | Shaidurikha | Trans-Urals | IA | -20.0 | 12.4 | Homo sapiens | Privat 2004 |
| SKA 2 | Skaty I | Trans-Urals | IA | -19.6 | 10.8 | Homo sapiens | Privat 2004 |
| SKA 3 | Skaty I | Trans-Urals | IA | -20.2 | 11.1 | Homo sapiens | Privat 2004 |
| BKAR 15 | Bolshekaragansky | Trans-Urals | MBA | -19.7 | 4.7 | Equus caballus | Privat 2004 |
| BKAR 17 | Bolshekaragansky | Trans-Urals | MBA | -18.5 | 6.4 | Ovis aries | Privat 2004 |
| KA-5, K4, Pit 9 | Kamenni Ambar V | Trans-Urals | MBA | -20.8 | 3.2 | Equus | Hanks et al. 2018 |
| KA-5, K4, Pit 8 | Kamenni Ambar V | Trans-Urals | MBA | -20.6 | 4.9 | Equus | Hanks et al. 2018 |
| KA-5, K4, Pit – | Kamenni Ambar V | Trans-Urals | MBA | -20.0 | 4.1 | Equus | Hanks et al. 2018 |
| KAM 3 | Kamenni Ambar V | Trans-Urals | MBA | -19.5 | 5.2 | Equus caballus | Privat 2004 |
| KAM 4 | Kamenni Ambar V | Trans-Urals | MBA | -19.9 | 6.5 | Equus caballus | Privat 2004 |
| KAM 5 | Kamenni Ambar V | Trans-Urals | MBA | -19.8 | 5.4 | Equus caballus | Privat 2004 |
| KAM 6 | Kamenni Ambar V | Trans-Urals | MBA | -20.1 | 6.3 | Equus caballus | Privat 2004 |
| KAM 7 | Kamenni Ambar V | Trans-Urals | MBA | -20.0 | 4.4 | Equus caballus | Privat 2004 |
| BKAR 16 | Bolshekaragansky | Trans-Urals | MBA | -19.0 | 6.9 | Bos taurus | Privat 2004 |
| KA-5, K4, Pit 9 | Kamenni Ambar V | Trans-Urals | MBA | -19.5 | 6.5 | Bos | Hanks et al. 2018 |
| KA-5, K4, Pit 2, A | Kamenni Ambar V | Trans-Urals | MBA | -19.8 | 6.4 | Bos | Hanks et al. 2018 |
| KA-5, K4, Pit 2, B | Kamenni Ambar V | Trans-Urals | MBA | -18.7 | 8.8 | Bos | Hanks et al. 2018 |
| KAM 16 | Kamenni Ambar V | Trans-Urals | MBA | -18.4 | 9.4 | Bos taurus | Privat 2004 |
| KAM 17 | Kamenni Ambar V | Trans-Urals | MBA | -19.1 | 5.4 | Bos taurus | Privat 2004 |
| KAM 18 | Kamenni Ambar V | Trans-Urals | MBA | -18.6 | 6.1 | Bos taurus | Privat 2004 |
| KAM 10 | Kamenni Ambar V | Trans-Urals | MBA | -17.9 | 8.1 | ovicaprid | Privat 2004 |

| | | | | | | | |
|---|---|---|---|---|---|---|---|
| KAM 11 | Kamenni Ambar V | Trans-Urals | MBA | -17.3 | 8.8 | ovicaprid | Privat 2004 |
| KAM 12 | Kamenni Ambar V | Trans-Urals | MBA | -18.4 | 7.1 | ovicaprid | Privat 2004 |
| KAM 13 | Kamenni Ambar V | Trans-Urals | MBA | -18.2 | 8.4 | ovicaprid | Privat 2004 |
| KAM 8 | Kamenni Ambar V | Trans-Urals | MBA | -19.0 | 4.3 | ovicaprid | Privat 2004 |
| KAM 9 | Kamenni Ambar V | Trans-Urals | MBA | -17.1 | 9.4 | ovicaprid | Privat 2004 |
| KA-5, K4, Pit 2 | Kamenni Ambar V | Trans-Urals | MBA | -19.0 | 7.9 | Ovis/Capra | Hanks et al. 2018 |
| KA-5, K4, Pit 8 | Kamenni Ambar V | Trans-Urals | MBA | -18.5 | 6.1 | Ovis/Capra | Hanks et al. 2018 |
| BAI 1 | Baitovo | Trans-Urals | IA | -20.0 | 5.4 | Bos | Privat 2004 |
| BAI 2 | Baitovo | Trans-Urals | IA | -21.4 | 5.7 | Bos | Privat 2004 |
| BAI 3 | Baitovo | Trans-Urals | IA | -20.1 | 5.7 | Bos | Privat 2004 |
| BAI 4 | Baitovo | Trans-Urals | IA | -20.7 | 6.2 | Bos | Privat 2004 |
| BAI 7 | Baitovo | Trans-Urals | IA | -21.3 | 5.6 | Equus | Privat 2004 |
| BAI 8 | Baitovo | Trans-Urals | IA | -21.5 | 4.1 | Equus | Privat 2004 |
| BAI 9 | Baitovo | Trans-Urals | IA | -21.7 | 4.5 | Equus | Privat 2004 |
| MUR 9 | Murzino | Trans-Urals | IA | -20.5 | 7.3 | Bos | Privat 2004 |
| PAV 1 | Pavlinova | Trans-Urals | IA | -20.8 | 5.9 | Bos | Privat 2004 |
| PAV 18 | Pavlinova | Trans-Urals | IA | -21.3 | 5.4 | Bos | Privat 2004 |
| PAV 19 | Pavlinova | Trans-Urals | IA | -20.7 | 5.6 | Bos | Privat 2004 |
| PAV 2 | Pavlinova | Trans-Urals | IA | -20.4 | 6.2 | Bos | Privat 2004 |
| PAV 3 | Pavlinova | Trans-Urals | IA | -20.4 | 5.6 | Bos | Privat 2004 |
| PAV 4 | Pavlinova | Trans-Urals | IA | -20.4 | 4.0 | Bos | Privat 2004 |
| PAV 5 | Pavlinova | Trans-Urals | IA | -20.9 | 6.5 | Bos | Privat 2004 |
| PAV 10 | Pavlinova | Trans-Urals | IA | -21.1 | 4.1 | Equus | Privat 2004 |
| PAV 20 | Pavlinova | Trans-Urals | IA | -21.4 | 4.6 | Equus | Privat 2004 |
| PAV 21 | Pavlinova | Trans-Urals | IA | -20.1 | 7.5 | Equus | Privat 2004 |
| PAV 6 | Pavlinova | Trans-Urals | IA | -21.1 | 6.3 | Equus | Privat 2004 |
| PAV 7 | Pavlinova | Trans-Urals | IA | -20.7 | 4.3 | Equus | Privat 2004 |

| | | | | | | | |
|---|---|---|---|---|---|---|---|
| PAV 8 | Pavlinova | Trans-Urals | IA | -21.0 | 4.6 | Equus | Privat 2004 |
| PAV 9 | Pavlinova | Trans-Urals | IA | -20.3 | 4.0 | Equus | Privat 2004 |
| PAV 11 | Pavlinova | Trans-Urals | IA | -17.7 | 8.8 | ovicaprid | Privat 2004 |
| PAV 12 | Pavlinova | Trans-Urals | IA | -18.1 | 9.4 | ovicaprid | Privat 2004 |
| PAV 13 | Pavlinova | Trans-Urals | IA | -19.6 | 9.1 | ovicaprid | Privat 2004 |
| PAV 14 | Pavlinova | Trans-Urals | IA | -18.7 | 8.7 | ovicaprid | Privat 2004 |
| PAV 15 | Pavlinova | Trans-Urals | IA | -20.3 | 6.5 | ovicaprid | Privat 2004 |
| PAV 16 | Pavlinova | Trans-Urals | IA | -19.9 | 4.6 | ovicaprid | Privat 2004 |
| PAV 17 | Pavlinova | Trans-Urals | IA | -18.9 | 8.4 | ovicaprid | Privat 2004 |
| SKA 1 | Skaty | Trans-Urals | IA | -21.2 | 4.1 | Equus | Privat 2004 |
| BES 1 | Bestamak | Trans-Urals | MBA | -20.0 | 6.2 | Equus caballus | Privat 2004 |
| BES 2 | Bestamak | Trans-Urals | MBA | -17.7 | 7.8 | Ovis aries | Privat 2004 |
| L3067-HORSE | Lisakovsk | Trans-Urals | LBA | -20.2 | 4.6 | Equus | Ventresca Miller et al. 2014 |
| L3177-SHEEP | Lisakovsk | Trans-Urals | LBA | -19.4 | 6.0 | Ovis | Ventresca Miller et al. 2014 |
| BES 3 | Bestamak | Trans-Urals | MBA | -18.5 | 8.4 | Bos taurus | Privat 2004 |
| L3023-COW | Lisakovsk | Trans-Urals | LBA | -19.0 | 8.3 | Bos | Ventresca Miller et al. 2014 |
| L3159-S G | Lisakovsk | Trans-Urals | LBA | -18.8 | 7.1 | Ovis/Capra | Ventresca Miller et al. 2014 |
| L 0-1 S G | Lisakovsk | Trans-Urals | LBA | -19.4 | 7.4 | Ovis/Capra | Ventresca Miller et al. 2014 |
| GM_F79 | Lisakovsk | Trans-Urals | LBA | -18.7 | 7.8 | caprine | Motuzaite Matuzeviciute et al. 2016 |
| GM_F80 | Lisakovsk | Trans-Urals | LBA | -19.8 | 5.3 | caprine | Motuzaite Matuzeviciute et al. 2016 |
| GM_F82 | Lisakovsk | Trans-Urals | LBA | -19.1 | 6.1 | caprine | Motuzaite Matuzeviciute et al. 2016 |
| GM_F82 | Lisakovsk | Trans-Urals | LBA | -19.1 | 6.1 | caprine | Motuzaite Matuzeviciute et al. 2016 |
| GM_F83 | Lisakovsk | Trans-Urals | LBA | -19.2 | 6.1 | caprine | Motuzaite Matuzeviciute et al. 2016 |
| GM_F83 | Lisakovsk | Trans-Urals | LBA | -19.2 | 6.1 | caprine | Motuzaite Matuzeviciute et al. 2016 |
| GM_F84 | Lisakovsk | Trans-Urals | LBA | -18.8 | 6.2 | caprine | Motuzaite Matuzeviciute et al. 2016 |
| GM_F85 | Lisakovsk | Trans-Urals | LBA | -19.5 | 6.0 | caprine | Motuzaite Matuzeviciute et al. 2016 |
| GM_F86 | Lisakovsk | Trans-Urals | LBA | -19.5 | 5.7 | caprine | Motuzaite Matuzeviciute et al. 2016 |

| | | | | | | | | |
|---|---|---|---|---|---|---|---|---|
| GM_F86 | Lisakovsk | Trans-Urals | LBA | -19.5 | 5.7 | caprine | Motuzaite Matuzeviciute et al. 2016 | |
| GM_F101 | Lisakovsk | Trans-Urals | LBA | -19.8 | 5.4 | cattle | Motuzaite Matuzeviciute et al. 2016 | |
| GM_F102 | Lisakovsk | Trans-Urals | LBA | -19.8 | 6.5 | cattle | Motuzaite Matuzeviciute et al. 2016 | |
| GM_F87 | Lisakovsk | Trans-Urals | LBA | -19.8 | 5.8 | cattle | Motuzaite Matuzeviciute et al. 2016 | |
| GM_F89 | Lisakovsk | Trans-Urals | LBA | -19.8 | 5.7 | cattle | Motuzaite Matuzeviciute et al. 2016 | |
| GM_F90 | Lisakovsk | Trans-Urals | LBA | -19.8 | 7.0 | cattle | Motuzaite Matuzeviciute et al. 2016 | |
| GM_F95 | Lisakovsk | Trans-Urals | LBA | -19.4 | 5.6 | cattle | Motuzaite Matuzeviciute et al. 2016 | |
| GM_F96 | Lisakovsk | Trans-Urals | LBA | -19.3 | 5.3 | cattle | Motuzaite Matuzeviciute et al. 2016 | |
| GM_F97 | Lisakovsk | Trans-Urals | LBA | -19.7 | 6.6 | cattle | Motuzaite Matuzeviciute et al. 2016 | |
| GM_F98 | Lisakovsk | Trans-Urals | LBA | -19.5 | 6.1 | cattle | Motuzaite Matuzeviciute et al. 2016 | |
| GM_F99 | Lisakovsk | Trans-Urals | LBA | -19.1 | 5.9 | cattle | Motuzaite Matuzeviciute et al. 2016 | |
| GM_F94 | Lisakovsk | Trans-Urals | LBA | -20.1 | 5.8 | horse | Motuzaite Matuzeviciute et al. 2016 | |
| GM_F93 | Lisakovsk | Trans-Urals | LBA | -20.4 | 6.1 | horse | Motuzaite Matuzeviciute et al. 2016 | |
| GM_F91 | Lisakovsk | Trans-Urals | LBA | -20.4 | 6.5 | horse | Motuzaite Matuzeviciute et al. 2016 | |
| GM_F100 | Lisakovsk | Trans-Urals | LBA | -20.8 | 4.3 | horse | Motuzaite Matuzeviciute et al. 2016 | |
| Not Available | Afanas’eva Gora | Minusinsk Basin | LN | -19.1 | 13.4 | Homo sapiens | Svyatko et al. 2007 | * |
| Not Available | Afanas'eva Gora | Minusinsk Basin | LN | -18.9 | 13.1 | Homo sapiens | Svyatko et al. 2007 | * |
| Not Available | Afanas'eva Gora | Minusinsk Basin | LN | -19.1 | 12.3 | Homo sapiens | Svyatko et al. 2007 | * |
| Not Available | Afanas'eva Gora | Minusinsk Basin | LN | -19.2 | 12.2 | Homo sapiens | Svyatko et al. 2007 | * |
| Not Available | Afanas'eva Gora | Minusinsk Basin | LN | -19.4 | 12.5 | Homo sapiens | Svyatko et al. 2007 | * |
| Not Available | Afanas'eva Gora | Minusinsk Basin | LN | -18.6 | 12.2 | Homo sapiens | Svyatko et al. 2007 | * |
| Not Available | Afanas'eva Gora | Minusinsk Basin | LN | -19.0 | 11.7 | Homo sapiens | Svyatko et al. 2007 | * |
| Not Available | Karasuk III | Minusinsk Basin | LN | -18.5 | 11.9 | Homo sapiens | Svyatko et al. 2007 | * |
| Not Available | Karasuk III | Minusinsk Basin | LN | -18.8 | 12.0 | Homo sapiens | Svyatko et al. 2007 | * |
| Not Available | Karasuk III | Minusinsk Basin | LN | -19.0 | 12.6 | Homo sapiens | Svyatko et al. 2007 | * |
| Not Available | Karasuk III | Minusinsk Basin | LN | -19.1 | 11.8 | Homo sapiens | Svyatko et al. 2007 | * |
| Not Available | Karasuk III | Minusinsk Basin | LN | -19.2 | 11.5 | Homo sapiens | Svyatko et al. 2007 | * |

| | | | | | | | | |
|---|---|---|---|---|---|---|---|---|
| Not Available | Karasuk III | Minusinsk Basin | LN | -19.1 | 11.3 | Homo sapiens | Svyatko et al. 2007 | * |
| Not Available | Karasuk III | Minusinsk Basin | LN | -19.0 | 11.2 | Homo sapiens | Svyatko et al. 2007 | * |
| Not Available | Karasuk III | Minusinsk Basin | LN | -19.3 | 11.2 | Homo sapiens | Svyatko et al. 2007 | * |
| Not Available | Karasuk III | Minusinsk Basin | LN | -18.7 | 11.4 | Homo sapiens | Svyatko et al. 2007 | * |
| Not Available | Karasuk III | Minusinsk Basin | LN | -18.6 | 11.8 | Homo sapiens | Svyatko et al. 2007 | * |
| Not Available | Karasuk III | Minusinsk Basin | LN | -19.0 | 11.3 | Homo sapiens | Svyatko et al. 2007 | * |
| Not Available | Karasuk III | Minusinsk Basin | LN | -19.0 | 11.1 | Homo sapiens | Svyatko et al. 2007 | * |
| Not Available | Karasuk III | Minusinsk Basin | LN | -19.1 | 11.5 | Homo sapiens | Svyatko et al. 2007 | * |
| Not Available | Karasuk III | Minusinsk Basin | LN | -19.3 | 11.2 | Homo sapiens | Svyatko et al. 2007 | * |
| Not Available | Karasuk III | Minusinsk Basin | LN | -19.6 | 11.1 | Homo sapiens | Svyatko et al. 2007 | * |
| Not Available | Karasuk III | Minusinsk Basin | LN | -19.2 | 10.8 | Homo sapiens | Svyatko et al. 2007 | * |
| Not Available | Karasuk III | Minusinsk Basin | LN | -19.3 | 11.6 | Homo sapiens | Svyatko et al. 2007 | * |
| Not Available | Karasuk III | Minusinsk Basin | LN | -19.3 | 10.8 | Homo sapiens | Svyatko et al. 2007 | * |
| Not Available | Karasuk III | Minusinsk Basin | LN | -19.5 | 10.6 | Homo sapiens | Svyatko et al. 2007 | * |
| Not Available | Karasuk III | Minusinsk Basin | LN | -19.9 | 11.3 | Homo sapiens | Svyatko et al. 2007 | * |
| Not Available | Uknown | Minusinsk Basin | LN | -19.1 | 13.4 | Homo sapiens | Svyatko et al. 2007 | * |
| Not Available | Uknown | Minusinsk Basin | LN | -18.9 | 13.1 | Homo sapiens | Svyatko et al. 2007 | * |
| Not Available | Uknown | Minusinsk Basin | LN | -19.0 | 11.4 | Homo sapiens | Svyatko et al. 2007 | * |
| Not Available | Uknown | Minusinsk Basin | LN | -19.1 | 12.4 | Homo sapiens | Svyatko et al. 2007 | * |
| Not Available | Uknown | Minusinsk Basin | LN | -19.2 | 12.3 | Homo sapiens | Svyatko et al. 2007 | * |
| Not Available | Uknown | Minusinsk Basin | LN | -19.4 | 12.6 | Homo sapiens | Svyatko et al. 2007 | * |
| Not Available | Uknown | Minusinsk Basin | LN | -18.8 | 12.0 | Homo sapiens | Svyatko et al. 2007 | * |
| Not Available | Uknown | Minusinsk Basin | LN | -18.6 | 11.8 | Homo sapiens | Svyatko et al. 2007 | * |
| Not Available | Uknown | Minusinsk Basin | LN | -18.6 | 12.2 | Homo sapiens | Svyatko et al. 2007 | * |
| Not Available | Uknown | Minusinsk Basin | LN | -18.7 | 11.4 | Homo sapiens | Svyatko et al. 2007 | * |
| Not Available | Uknown | Minusinsk Basin | LN | -19.0 | 11.1 | Homo sapiens | Svyatko et al. 2007 | * |
| Not Available | Uknown | Minusinsk Basin | LN | -19.1 | 11.4 | Homo sapiens | Svyatko et al. 2007 | * |

| | | | | | | | | |
|---|---|---|---|---|---|---|---|---|
| Not Available | Uknown | Minusinsk Basin | LN | -19.2 | 11.5 | Homo sapiens | Svyatko et al. 2007 | * |
| Not Available | Uknown | Minusinsk Basin | LN | -19.1 | 11.6 | Homo sapiens | Svyatko et al. 2007 | * |
| Not Available | Uknown | Minusinsk Basin | LN | -19.3 | 11.6 | Homo sapiens | Svyatko et al. 2007 | * |
| Not Available | Uknown | Minusinsk Basin | LN | -19.1 | 11.8 | Homo sapiens | Svyatko et al. 2007 | * |
| Not Available | Uknown | Minusinsk Basin | LN | -19.6 | 11.2 | Homo sapiens | Svyatko et al. 2007 | * |
| Not Available | Uknown | Minusinsk Basin | LN | -19.6 | 11.1 | Homo sapiens | Svyatko et al. 2007 | * |
| Not Available | Uknown | Minusinsk Basin | LN | -19.3 | 11.2 | Homo sapiens | Svyatko et al. 2007 | * |
| Not Available | Uknown | Minusinsk Basin | LN | -19.2 | 10.8 | Homo sapiens | Svyatko et al. 2007 | * |
| Not Available | Uknown | Minusinsk Basin | LN | -19.5 | 10.6 | Homo sapiens | Svyatko et al. 2007 | * |
| Not Available | Uknown | Minusinsk Basin | LN | -19.8 | 10.3 | Homo sapiens | Svyatko et al. 2007 | * |
| Not Available | Uknown | Minusinsk Basin | LN | -16.9 | 13.0 | Homo sapiens | Svyatko et al. 2007 | * |
| Not Available | Uknown | Minusinsk Basin | LN | -16.7 | 12.8 | Homo sapiens | Svyatko et al. 2007 | * |
| Not Available | Uknown | Minusinsk Basin | EBA | -18.6 | 10.5 | Homo sapiens | Svyatko et al. 2007 | * |
| Not Available | Uknown | Minusinsk Basin | EBA | -18.6 | 10.9 | Homo sapiens | Svyatko et al. 2007 | * |
| Not Available | Uknown | Minusinsk Basin | EBA | -18.2 | 10.7 | Homo sapiens | Svyatko et al. 2007 | * |
| Not Available | Uknown | Minusinsk Basin | EBA | -18.1 | 10.7 | Homo sapiens | Svyatko et al. 2007 | * |
| Not Available | Uknown | Minusinsk Basin | EBA | -18.2 | 10.9 | Homo sapiens | Svyatko et al. 2007 | * |
| Not Available | Uknown | Minusinsk Basin | EBA | -18.0 | 11.0 | Homo sapiens | Svyatko et al. 2007 | * |
| Not Available | Uknown | Minusinsk Basin | EBA | -17.9 | 11.2 | Homo sapiens | Svyatko et al. 2007 | * |
| Not Available | Uknown | Minusinsk Basin | EBA | -17.7 | 11.0 | Homo sapiens | Svyatko et al. 2007 | * |
| Not Available | Uknown | Minusinsk Basin | EBA | -18.4 | 11.0 | Homo sapiens | Svyatko et al. 2007 | * |
| Not Available | Uknown | Minusinsk Basin | EBA | -18.6 | 11.1 | Homo sapiens | Svyatko et al. 2007 | * |
| Not Available | Uknown | Minusinsk Basin | EBA | -18.8 | 11.0 | Homo sapiens | Svyatko et al. 2007 | * |
| Not Available | Uknown | Minusinsk Basin | EBA | -18.7 | 11.1 | Homo sapiens | Svyatko et al. 2007 | * |
| Not Available | Uknown | Minusinsk Basin | EBA | -18.7 | 11.2 | Homo sapiens | Svyatko et al. 2007 | * |
| Not Available | Uknown | Minusinsk Basin | EBA | -18.6 | 11.2 | Homo sapiens | Svyatko et al. 2007 | * |
| Not Available | Uknown | Minusinsk Basin | EBA | -18.5 | 11.2 | Homo sapiens | Svyatko et al. 2007 | * |

| | | | | | | | | |
|---|---|---|---|---|---|---|---|---|
| Not Available | Uknown | Minusinsk Basin | EBA | -18.5 | 11.2 | Homo sapiens | Svyatko et al. 2007 | * |
| Not Available | Uknown | Minusinsk Basin | EBA | -18.5 | 11.3 | Homo sapiens | Svyatko et al. 2007 | * |
| Not Available | Uknown | Minusinsk Basin | EBA | -18.5 | 11.4 | Homo sapiens | Svyatko et al. 2007 | * |
| Not Available | Uknown | Minusinsk Basin | EBA | -18.4 | 11.5 | Homo sapiens | Svyatko et al. 2007 | * |
| Not Available | Uknown | Minusinsk Basin | EBA | -18.5 | 11.5 | Homo sapiens | Svyatko et al. 2007 | * |
| Not Available | Uknown | Minusinsk Basin | EBA | -18.4 | 11.3 | Homo sapiens | Svyatko et al. 2007 | * |
| Not Available | Uknown | Minusinsk Basin | EBA | -18.3 | 11.2 | Homo sapiens | Svyatko et al. 2007 | * |
| Not Available | Uknown | Minusinsk Basin | EBA | -18.2 | 11.3 | Homo sapiens | Svyatko et al. 2007 | * |
| Not Available | Uknown | Minusinsk Basin | EBA | -18.2 | 11.4 | Homo sapiens | Svyatko et al. 2007 | * |
| Not Available | Uknown | Minusinsk Basin | EBA | -18.2 | 11.4 | Homo sapiens | Svyatko et al. 2007 | * |
| Not Available | Uknown | Minusinsk Basin | EBA | -18.1 | 11.6 | Homo sapiens | Svyatko et al. 2007 | * |
| Not Available | Uknown | Minusinsk Basin | EBA | -18.0 | 11.6 | Homo sapiens | Svyatko et al. 2007 | * |
| Not Available | Uknown | Minusinsk Basin | EBA | -17.8 | 11.7 | Homo sapiens | Svyatko et al. 2007 | * |
| Not Available | Uknown | Minusinsk Basin | EBA | -19.1 | 11.0 | Homo sapiens | Svyatko et al. 2007 | * |
| Not Available | Uknown | Minusinsk Basin | EBA | -19.0 | 11.3 | Homo sapiens | Svyatko et al. 2007 | * |
| Not Available | Uknown | Minusinsk Basin | EBA | -18.9 | 11.3 | Homo sapiens | Svyatko et al. 2007 | * |
| Not Available | Uknown | Minusinsk Basin | EBA | -18.8 | 11.2 | Homo sapiens | Svyatko et al. 2007 | * |
| Not Available | Uknown | Minusinsk Basin | EBA | -18.8 | 11.3 | Homo sapiens | Svyatko et al. 2007 | * |
| Not Available | Uknown | Minusinsk Basin | EBA | -18.7 | 11.2 | Homo sapiens | Svyatko et al. 2007 | * |
| Not Available | Uknown | Minusinsk Basin | EBA | -18.7 | 11.4 | Homo sapiens | Svyatko et al. 2007 | * |
| Not Available | Uknown | Minusinsk Basin | EBA | -18.7 | 11.5 | Homo sapiens | Svyatko et al. 2007 | * |
| Not Available | Uknown | Minusinsk Basin | EBA | -18.8 | 11.5 | Homo sapiens | Svyatko et al. 2007 | * |
| Not Available | Uknown | Minusinsk Basin | EBA | -18.8 | 11.6 | Homo sapiens | Svyatko et al. 2007 | * |
| Not Available | Uknown | Minusinsk Basin | EBA | -18.8 | 11.7 | Homo sapiens | Svyatko et al. 2007 | * |
| Not Available | Uknown | Minusinsk Basin | EBA | -18.8 | 11.8 | Homo sapiens | Svyatko et al. 2007 | * |
| Not Available | Uknown | Minusinsk Basin | EBA | -18.7 | 11.8 | Homo sapiens | Svyatko et al. 2007 | * |
| Not Available | Uknown | Minusinsk Basin | EBA | -18.6 | 11.7 | Homo sapiens | Svyatko et al. 2007 | * |

| | | | | | | | | |
|---|---|---|---|---|---|---|---|---|
| Not Available | Uknown | Minusinsk Basin | EBA | -18.6 | 11.7 | Homo sapiens | Svyatko et al. 2007 | * |
| Not Available | Uknown | Minusinsk Basin | EBA | -18.7 | 11.5 | Homo sapiens | Svyatko et al. 2007 | * |
| Not Available | Uknown | Minusinsk Basin | EBA | -18.7 | 11.6 | Homo sapiens | Svyatko et al. 2007 | * |
| Not Available | Uknown | Minusinsk Basin | EBA | -18.8 | 11.7 | Homo sapiens | Svyatko et al. 2007 | * |
| Not Available | Uknown | Minusinsk Basin | EBA | -18.7 | 11.7 | Homo sapiens | Svyatko et al. 2007 | * |
| Not Available | Uknown | Minusinsk Basin | EBA | -18.5 | 12.0 | Homo sapiens | Svyatko et al. 2007 | * |
| Not Available | Uknown | Minusinsk Basin | EBA | -18.5 | 11.9 | Homo sapiens | Svyatko et al. 2007 | * |
| Not Available | Uknown | Minusinsk Basin | EBA | -18.4 | 11.9 | Homo sapiens | Svyatko et al. 2007 | * |
| Not Available | Uknown | Minusinsk Basin | EBA | -18.1 | 11.9 | Homo sapiens | Svyatko et al. 2007 | * |
| Not Available | Uknown | Minusinsk Basin | EBA | -18.5 | 12.2 | Homo sapiens | Svyatko et al. 2007 | * |
| Not Available | Uknown | Minusinsk Basin | EBA | -18.4 | 12.3 | Homo sapiens | Svyatko et al. 2007 | * |
| Not Available | Uknown | Minusinsk Basin | EBA | -18.5 | 12.4 | Homo sapiens | Svyatko et al. 2007 | * |
| Not Available | Uknown | Minusinsk Basin | EBA | -18.5 | 12.4 | Homo sapiens | Svyatko et al. 2007 | * |
| Not Available | Uknown | Minusinsk Basin | EBA | -18.6 | 12.3 | Homo sapiens | Svyatko et al. 2007 | * |
| Not Available | Uknown | Minusinsk Basin | EBA | -18.6 | 12.5 | Homo sapiens | Svyatko et al. 2007 | * |
| Not Available | Uknown | Minusinsk Basin | EBA | -18.6 | 12.2 | Homo sapiens | Svyatko et al. 2007 | * |
| Not Available | Uknown | Minusinsk Basin | EBA | -18.9 | 12.2 | Homo sapiens | Svyatko et al. 2007 | * |
| Not Available | Uknown | Minusinsk Basin | EBA | -19.0 | 11.7 | Homo sapiens | Svyatko et al. 2007 | * |
| Not Available | Uknown | Minusinsk Basin | EBA | -19.0 | 11.6 | Homo sapiens | Svyatko et al. 2007 | * |
| Not Available | Uknown | Minusinsk Basin | EBA | -19.2 | 11.6 | Homo sapiens | Svyatko et al. 2007 | * |
| Not Available | Uknown | Minusinsk Basin | EBA | -19.2 | 11.7 | Homo sapiens | Svyatko et al. 2007 | * |
| Not Available | Uknown | Minusinsk Basin | EBA | -19.1 | 11.9 | Homo sapiens | Svyatko et al. 2007 | * |
| Not Available | Uknown | Minusinsk Basin | EBA | -19.0 | 11.9 | Homo sapiens | Svyatko et al. 2007 | * |
| Not Available | Uknown | Minusinsk Basin | EBA | -19.5 | 11.7 | Homo sapiens | Svyatko et al. 2007 | * |
| Not Available | Uknown | Minusinsk Basin | EBA | -19.4 | 12.3 | Homo sapiens | Svyatko et al. 2007 | * |
| Not Available | Uknown | Minusinsk Basin | EBA | -19.2 | 12.4 | Homo sapiens | Svyatko et al. 2007 | * |
| Not Available | Uknown | Minusinsk Basin | EBA | -19.1 | 12.2 | Homo sapiens | Svyatko et al. 2007 | * |

| | | | | | | | | |
|---|---|---|---|---|---|---|---|---|
| Not Available | Uknown | Minusinsk Basin | EBA | -15.9 | 11.5 | Homo sapiens | Svyatko et al. 2007 | * |
| Not Available | Uknown | Minusinsk Basin | LBA | -17.6 | 11.4 | Homo sapiens | Svyatko et al. 2007 | * |
| Not Available | Uknown | Minusinsk Basin | LBA | -17.6 | 11.2 | Homo sapiens | Svyatko et al. 2007 | * |
| Not Available | Uknown | Minusinsk Basin | LBA | -17.5 | 11.3 | Homo sapiens | Svyatko et al. 2007 | * |
| Not Available | Uknown | Minusinsk Basin | LBA | -17.5 | 11.2 | Homo sapiens | Svyatko et al. 2007 | * |
| Not Available | Uknown | Minusinsk Basin | LBA | -17.6 | 11.2 | Homo sapiens | Svyatko et al. 2007 | * |
| Not Available | Uknown | Minusinsk Basin | LBA | -17.6 | 11.1 | Homo sapiens | Svyatko et al. 2007 | * |
| Not Available | Uknown | Minusinsk Basin | LBA | -18.0 | 10.9 | Homo sapiens | Svyatko et al. 2007 | * |
| Not Available | Uknown | Minusinsk Basin | LBA | -17.9 | 10.7 | Homo sapiens | Svyatko et al. 2007 | * |
| Not Available | Uknown | Minusinsk Basin | LBA | -18.0 | 10.4 | Homo sapiens | Svyatko et al. 2007 | * |
| Not Available | Uknown | Minusinsk Basin | LBA | -17.8 | 10.9 | Homo sapiens | Svyatko et al. 2007 | * |
| Not Available | Uknown | Minusinsk Basin | LBA | -17.7 | 10.9 | Homo sapiens | Svyatko et al. 2007 | * |
| Not Available | Uknown | Minusinsk Basin | LBA | -17.7 | 10.8 | Homo sapiens | Svyatko et al. 2007 | * |
| Not Available | Uknown | Minusinsk Basin | LBA | -17.4 | 10.4 | Homo sapiens | Svyatko et al. 2007 | * |
| Not Available | Uknown | Minusinsk Basin | LBA | -17.3 | 10.8 | Homo sapiens | Svyatko et al. 2007 | * |
| Not Available | Uknown | Minusinsk Basin | LBA | -17.2 | 10.7 | Homo sapiens | Svyatko et al. 2007 | * |
| Not Available | Uknown | Minusinsk Basin | LBA | -16.7 | 10.3 | Homo sapiens | Svyatko et al. 2007 | * |
| Not Available | Uknown | Minusinsk Basin | LBA | -17.2 | 10.9 | Homo sapiens | Svyatko et al. 2007 | * |
| Not Available | Uknown | Minusinsk Basin | LBA | -17.2 | 11.1 | Homo sapiens | Svyatko et al. 2007 | * |
| Not Available | Uknown | Minusinsk Basin | LBA | -16.9 | 11.3 | Homo sapiens | Svyatko et al. 2007 | * |
| Not Available | Uknown | Minusinsk Basin | LBA | -16.9 | 11.6 | Homo sapiens | Svyatko et al. 2007 | * |
| Not Available | Uknown | Minusinsk Basin | LBA | -17.2 | 12.0 | Homo sapiens | Svyatko et al. 2007 | * |
| Not Available | Uknown | Minusinsk Basin | LBA | -17.0 | 12.2 | Homo sapiens | Svyatko et al. 2007 | * |
| Not Available | Uknown | Minusinsk Basin | LBA | -16.8 | 12.5 | Homo sapiens | Svyatko et al. 2007 | * |
| Not Available | Uknown | Minusinsk Basin | LBA | -16.8 | 11.9 | Homo sapiens | Svyatko et al. 2007 | * |
| Not Available | Uknown | Minusinsk Basin | LBA | -16.6 | 11.8 | Homo sapiens | Svyatko et al. 2007 | * |
| Not Available | Uknown | Minusinsk Basin | LBA | -16.5 | 11.3 | Homo sapiens | Svyatko et al. 2007 | * |

| | | | | | | | | |
|---|---|---|---|---|---|---|---|---|
| Not Available | Uknown | Minusinsk Basin | LBA | -16.5 | 11.3 | Homo sapiens | Svyatko et al. 2007 | * |
| Not Available | Uknown | Minusinsk Basin | LBA | -16.5 | 11.1 | Homo sapiens | Svyatko et al. 2007 | * |
| Not Available | Uknown | Minusinsk Basin | LBA | -16.4 | 11.0 | Homo sapiens | Svyatko et al. 2007 | * |
| Not Available | Uknown | Minusinsk Basin | LBA | -16.1 | 10.7 | Homo sapiens | Svyatko et al. 2007 | * |
| Not Available | Uknown | Minusinsk Basin | LBA | -16.1 | 10.7 | Homo sapiens | Svyatko et al. 2007 | * |
| Not Available | Uknown | Minusinsk Basin | LBA | -15.9 | 10.6 | Homo sapiens | Svyatko et al. 2007 | * |
| Not Available | Uknown | Minusinsk Basin | LBA | -15.9 | 10.2 | Homo sapiens | Svyatko et al. 2007 | * |
| Not Available | Uknown | Minusinsk Basin | LBA | -15.9 | 11.0 | Homo sapiens | Svyatko et al. 2007 | * |
| Not Available | Uknown | Minusinsk Basin | LBA | -15.9 | 11.1 | Homo sapiens | Svyatko et al. 2007 | * |
| Not Available | Uknown | Minusinsk Basin | LBA | -15.9 | 11.5 | Homo sapiens | Svyatko et al. 2007 | * |
| Not Available | Uknown | Minusinsk Basin | LBA | -16.1 | 11.7 | Homo sapiens | Svyatko et al. 2007 | * |
| Not Available | Uknown | Minusinsk Basin | LBA | -16.1 | 11.7 | Homo sapiens | Svyatko et al. 2007 | * |
| Not Available | Uknown | Minusinsk Basin | LBA | -16.0 | 11.7 | Homo sapiens | Svyatko et al. 2007 | * |
| Not Available | Uknown | Minusinsk Basin | LBA | -15.9 | 9.9 | Homo sapiens | Svyatko et al. 2007 | * |
| Not Available | Uknown | Minusinsk Basin | LBA | -15.8 | 10.8 | Homo sapiens | Svyatko et al. 2007 | * |
| Not Available | Uknown | Minusinsk Basin | LBA | -15.6 | 11.3 | Homo sapiens | Svyatko et al. 2007 | * |
| Not Available | Uknown | Minusinsk Basin | LBA | -15.8 | 11.7 | Homo sapiens | Svyatko et al. 2007 | * |
| Not Available | Uknown | Minusinsk Basin | LBA | -15.7 | 11.6 | Homo sapiens | Svyatko et al. 2007 | * |
| Not Available | Uknown | Minusinsk Basin | LBA | -15.5 | 11.9 | Homo sapiens | Svyatko et al. 2007 | * |
| Not Available | Uknown | Minusinsk Basin | LBA | -15.5 | 11.3 | Homo sapiens | Svyatko et al. 2007 | * |
| Not Available | Uknown | Minusinsk Basin | LBA | -15.4 | 11.5 | Homo sapiens | Svyatko et al. 2007 | * |
| Not Available | Uknown | Minusinsk Basin | LBA | -15.0 | 11.6 | Homo sapiens | Svyatko et al. 2007 | * |
| Not Available | Uknown | Minusinsk Basin | LBA | -14.9 | 11.8 | Homo sapiens | Svyatko et al. 2007 | * |
| Not Available | Uknown | Minusinsk Basin | LBA | -14.8 | 11.6 | Homo sapiens | Svyatko et al. 2007 | * |
| Not Available | Uknown | Minusinsk Basin | LBA | -14.7 | 11.4 | Homo sapiens | Svyatko et al. 2007 | * |
| Not Available | Uknown | Minusinsk Basin | LBA | -14.6 | 11.7 | Homo sapiens | Svyatko et al. 2007 | * |
| Not Available | Uknown | Minusinsk Basin | LBA | -14.5 | 11.7 | Homo sapiens | Svyatko et al. 2007 | * |

| | | | | | | | | |
|---|---|---|---|---|---|---|---|---|
| Not Available | Uknown | Minusinsk Basin | LBA | -14.3 | 11.8 | Homo sapiens | Svyatko et al. 2007 | * |
| Not Available | Uknown | Minusinsk Basin | LBA | -14.2 | 11.4 | Homo sapiens | Svyatko et al. 2007 | * |
| Not Available | Uknown | Minusinsk Basin | LBA | -14.5 | 10.6 | Homo sapiens | Svyatko et al. 2007 | * |
| Not Available | Uknown | Minusinsk Basin | LBA | -14.3 | 10.7 | Homo sapiens | Svyatko et al. 2007 | * |
| Not Available | Uknown | Minusinsk Basin | LBA | -14.2 | 10.6 | Homo sapiens | Svyatko et al. 2007 | * |
| Not Available | Uknown | Minusinsk Basin | LBA | -15.3 | 12.8 | Homo sapiens | Svyatko et al. 2007 | * |
| Not Available | Uknown | Minusinsk Basin | LBA | -14.4 | 12.3 | Homo sapiens | Svyatko et al. 2007 | * |
| Not Available | Uknown | Minusinsk Basin | LBA | -14.4 | 12.4 | Homo sapiens | Svyatko et al. 2007 | * |
| Not Available | Uknown | Minusinsk Basin | LBA | -14.2 | 12.2 | Homo sapiens | Svyatko et al. 2007 | * |
| Not Available | Uknown | Minusinsk Basin | LBA | -13.5 | 11.6 | Homo sapiens | Svyatko et al. 2007 | * |
| Not Available | Uknown | Minusinsk Basin | LBA | -13.2 | 12.6 | Homo sapiens | Svyatko et al. 2007 | * |
| Not Available | Uknown | Minusinsk Basin | LBA | -11.8 | 12.5 | Homo sapiens | Svyatko et al. 2007 | * |
| Not Available | Uknown | Minusinsk Basin | LBA | -12.0 | 11.7 | Homo sapiens | Svyatko et al. 2007 | * |
| Not Available | Uknown | Minusinsk Basin | LBA | -12.5 | 11.8 | Homo sapiens | Svyatko et al. 2007 | |
| Not Available | Aymyrlyg | Minusinsk Basin | IA | -14.2 | 13.0 | Homo sapiens | Murphy et al. 2013 | |
| Not Available | Aymyrlyg | Minusinsk Basin | IA | -15.3 | 14.0 | Homo sapiens | Murphy et al. 2013 | |
| Not Available | Aymyrlyg | Minusinsk Basin | IA | -15.7 | 13.8 | Homo sapiens | Murphy et al. 2013 | |
| Not Available | Aymyrlyg | Minusinsk Basin | IA | -13.6 | 13.0 | Homo sapiens | Murphy et al. 2013 | |
| Not Available | Aymyrlyg | Minusinsk Basin | IA | -13.9 | 12.3 | Homo sapiens | Murphy et al. 2013 | |
| Not Available | Aymyrlyg | Minusinsk Basin | IA | -13.2 | 11.8 | Homo sapiens | Murphy et al. 2013 | |
| Not Available | Aymyrlyg | Minusinsk Basin | IA | -17.2 | 14.0 | Homo sapiens | Murphy et al. 2013 | |
| Not Available | Aymyrlyg | Minusinsk Basin | IA | -16.2 | 13.2 | Homo sapiens | Murphy et al. 2013 | |
| Not Available | Aymyrlyg | Minusinsk Basin | IA | -15.0 | 13.2 | Homo sapiens | Murphy et al. 2013 | |
| Not Available | Aymyrlyg | Minusinsk Basin | IA | -15.2 | 12.8 | Homo sapiens | Murphy et al. 2013 | |
| Not Available | Aymyrlyg | Minusinsk Basin | IA | -13.9 | 12.9 | Homo sapiens | Murphy et al. 2013 | |
| Not Available | Aymyrlyg | Minusinsk Basin | IA | -16.2 | 13.9 | Homo sapiens | Murphy et al. 2013 | |
| Not Available | Aymyrlyg | Minusinsk Basin | IA | -16.8 | 13.6 | Homo sapiens | Murphy et al. 2013 | |

| | | | | | | | |
|---|---|---|---|---|---|---|---|
| Not Available | Aymyrlyg | Minusinsk Basin | IA | -14.1 | 13.0 | Homo sapiens | Murphy et al. 2013 |
| Not Available | Aymyrlyg | Minusinsk Basin | IA | -17.5 | 13.8 | Homo sapiens | Murphy et al. 2013 |
| Not Available | Aymyrlyg | Minusinsk Basin | IA | -14.9 | 13.1 | Homo sapiens | Murphy et al. 2013 |
| Not Available | Aymyrlyg | Minusinsk Basin | IA | -17.2 | 12.9 | Homo sapiens | Murphy et al. 2013 |
| Not Available | Aymyrlyg | Minusinsk Basin | IA | -15.1 | 13.1 | Homo sapiens | Murphy et al. 2013 |
| Not Available | Aymyrlyg | Minusinsk Basin | IA | -14.7 | 13.5 | Homo sapiens | Murphy et al. 2013 |
| Not Available | Aymyrlyg | Minusinsk Basin | IA | -15.5 | 13.3 | Homo sapiens | Murphy et al. 2013 |
| Not Available | Aymyrlyg | Minusinsk Basin | IA | -16.1 | 13.4 | Homo sapiens | Murphy et al. 2013 |
| Not Available | Ai-Dai | Minusinsk Basin | IA | -14.7 | 10.2 | Homo sapiens | Murphy et al. 2013 |
| Not Available | Ai-Dai | Minusinsk Basin | IA | -15.3 | 10.6 | Homo sapiens | Murphy et al. 2013 |
| Not Available | Ai-Dai | Minusinsk Basin | IA | -15.0 | 11.8 | Homo sapiens | Murphy et al. 2013 |
| Not Available | Ai-Dai | Minusinsk Basin | IA | -13.8 | 11.5 | Homo sapiens | Murphy et al. 2013 |
| Not Available | Ai-Dai | Minusinsk Basin | IA | -12.8 | 9.9 | Homo sapiens | Murphy et al. 2013 |
| Not Available | Ai-Dai | Minusinsk Basin | IA | -14.2 | 10.4 | Homo sapiens | Murphy et al. 2013 |
| Not Available | Ai-Dai | Minusinsk Basin | IA | -16.2 | 10.2 | Homo sapiens | Murphy et al. 2013 |
| Not Available | Ai-Dai | Minusinsk Basin | IA | -16.1 | 10.3 | Homo sapiens | Murphy et al. 2013 |
| Not Available | Ai-Dai | Minusinsk Basin | IA | -16.6 | 10.6 | Homo sapiens | Murphy et al. 2013 |
| Not Available | Ai-Dai | Minusinsk Basin | IA | -15.2 | 11.2 | Homo sapiens | Murphy et al. 2013 |
| Not Available | Ai-Dai | Minusinsk Basin | IA | -14.4 | 10.2 | Homo sapiens | Murphy et al. 2013 |
| Not Available | Ai-Dai | Minusinsk Basin | IA | -15.2 | 11.0 | Homo sapiens | Murphy et al. 2013 |
| Not Available | Ai-Dai | Minusinsk Basin | IA | -14.6 | 10.2 | Homo sapiens | Murphy et al. 2013 |
| Not Available | Ai-Dai | Minusinsk Basin | IA | -15.6 | 10.2 | Homo sapiens | Murphy et al. 2013 |
| Not Available | Ai-Dai | Minusinsk Basin | IA | -15.5 | 10.8 | Homo sapiens | Murphy et al. 2013 |
| Not Available | Ai-Dai | Minusinsk Basin | IA | -16.6 | 11.2 | Homo sapiens | Murphy et al. 2013 |
| Not Available | Ai-Dai | Minusinsk Basin | IA | -15.3 | 10.4 | Homo sapiens | Murphy et al. 2013 |
| Not Available | Ai-Dai | Minusinsk Basin | IA | -15.3 | 11.0 | Homo sapiens | Murphy et al. 2013 |
| Not Available | Tagar Culture | Minusinsk Basin | IA | -19.4 | 13.5 | Homo sapiens | Svyatko et al. 2007 * |

| | | | | | | | | |
|---|---|---|---|---|---|---|---|---|
| Not Available | Tagar Culture | Minusinsk Basin | IA | -19.2 | 13.5 | Homo sapiens | Svyatko et al. 2007 | * |
| Not Available | Tagar Culture | Minusinsk Basin | IA | -18.6 | 13.0 | Homo sapiens | Svyatko et al. 2007 | * |
| Not Available | Tagar Culture | Minusinsk Basin | IA | -18.8 | 12.8 | Homo sapiens | Svyatko et al. 2007 | * |
| Not Available | Tagar Culture | Minusinsk Basin | IA | -18.5 | 11.7 | Homo sapiens | Svyatko et al. 2007 | * |
| Not Available | Tagar Culture | Minusinsk Basin | IA | -17.8 | 12.2 | Homo sapiens | Svyatko et al. 2007 | * |
| Not Available | Tagar Culture | Minusinsk Basin | IA | -17.4 | 12.3 | Homo sapiens | Svyatko et al. 2007 | * |
| Not Available | Tagar Culture | Minusinsk Basin | IA | -17.6 | 11.9 | Homo sapiens | Svyatko et al. 2007 | * |
| Not Available | Tagar Culture | Minusinsk Basin | IA | -17.6 | 12.0 | Homo sapiens | Svyatko et al. 2007 | * |
| Not Available | Tagar Culture | Minusinsk Basin | IA | -17.9 | 11.4 | Homo sapiens | Svyatko et al. 2007 | * |
| Not Available | Tagar Culture | Minusinsk Basin | IA | -17.6 | 11.5 | Homo sapiens | Svyatko et al. 2007 | * |
| Not Available | Tagar Culture | Minusinsk Basin | IA | -17.6 | 11.5 | Homo sapiens | Svyatko et al. 2007 | * |
| Not Available | Tagar Culture | Minusinsk Basin | IA | -17.4 | 11.4 | Homo sapiens | Svyatko et al. 2007 | * |
| Not Available | Tagar Culture | Minusinsk Basin | IA | -17.2 | 11.5 | Homo sapiens | Svyatko et al. 2007 | * |
| Not Available | Tagar Culture | Minusinsk Basin | IA | -17.2 | 11.5 | Homo sapiens | Svyatko et al. 2007 | * |
| Not Available | Tagar Culture | Minusinsk Basin | IA | -17.1 | 11.5 | Homo sapiens | Svyatko et al. 2007 | * |
| Not Available | Tagar Culture | Minusinsk Basin | IA | -16.8 | 11.1 | Homo sapiens | Svyatko et al. 2007 | * |
| Not Available | Tagar Culture | Minusinsk Basin | IA | -16.4 | 11.1 | Homo sapiens | Svyatko et al. 2007 | * |
| Not Available | Tagar Culture | Minusinsk Basin | IA | -16.1 | 11.3 | Homo sapiens | Svyatko et al. 2007 | * |
| Not Available | Tagar Culture | Minusinsk Basin | IA | -16.0 | 11.5 | Homo sapiens | Svyatko et al. 2007 | * |
| Not Available | Tagar Culture | Minusinsk Basin | IA | -15.3 | 12.3 | Homo sapiens | Svyatko et al. 2007 | * |
| Not Available | Tagar Culture | Minusinsk Basin | IA | -15.8 | 12.6 | Homo sapiens | Svyatko et al. 2007 | * |
| Not Available | Tagar Culture | Minusinsk Basin | IA | -14.7 | 12.0 | Homo sapiens | Svyatko et al. 2007 | * |
| Not Available | Tagar Culture | Minusinsk Basin | IA | -15.1 | 11.3 | Homo sapiens | Svyatko et al. 2007 | * |
| Not Available | Tagar Culture | Minusinsk Basin | IA | -14.8 | 11.6 | Homo sapiens | Svyatko et al. 2007 | * |
| Not Available | Tagar Culture | Minusinsk Basin | IA | -14.3 | 11.8 | Homo sapiens | Svyatko et al. 2007 | * |
| Not Available | Tagar Culture | Minusinsk Basin | IA | -14.7 | 10.6 | Homo sapiens | Svyatko et al. 2007 | * |
| Not Available | Tagar Culture | Minusinsk Basin | IA | -15.7 | 10.4 | Homo sapiens | Svyatko et al. 2007 | * |

| | | | | | | | | |
|---|---|---|---|---|---|---|---|---|
| Not Available | Tagar Culture | Minusinsk Basin | IA | -16.0 | 10.8 | Homo sapiens | Svyatko et al. 2007 | * |
| Not Available | Tagar Culture | Minusinsk Basin | IA | -16.0 | 11.0 | Homo sapiens | Svyatko et al. 2007 | * |
| Not Available | Tagar Culture | Minusinsk Basin | IA | -17.0 | 10.4 | Homo sapiens | Svyatko et al. 2007 | * |
| Not Available | Tagar Culture | Minusinsk Basin | IA | -17.1 | 10.8 | Homo sapiens | Svyatko et al. 2007 | * |
| Not Available | Tagar Culture | Minusinsk Basin | IA | -17.3 | 10.4 | Homo sapiens | Svyatko et al. 2007 | * |
| Not Available | Tagar Culture | Minusinsk Basin | IA | -17.5 | 10.1 | Homo sapiens | Svyatko et al. 2007 | * |
| Not Available | Tagar Culture | Minusinsk Basin | IA | -17.5 | 10.9 | Homo sapiens | Svyatko et al. 2007 | * |
| Not Available | Tagar Culture | Minusinsk Basin | IA | -17.6 | 11.2 | Homo sapiens | Svyatko et al. 2007 | * |
| Not Available | Tagar Culture | Minusinsk Basin | IA | -18.9 | 10.4 | Homo sapiens | Svyatko et al. 2007 | * |
| Not Available | Tagar Culture | Minusinsk Basin | IA | -18.7 | 10.2 | Homo sapiens | Svyatko et al. 2007 | * |
| Not Available | Tagar Culture | Minusinsk Basin | IA | -18.2 | 10.6 | Homo sapiens | Svyatko et al. 2007 | * |
| Not Available | Tagar Culture | Minusinsk Basin | IA | -18.2 | 10.4 | Homo sapiens | Svyatko et al. 2007 | * |
| Not Available | Ai-Dai | Minusinsk Basin | IA | -20.0 | 7.5 | Herbivore | Murphy et al. 2013 | |
| Not Available | Aymyrlyg | Minusinsk Basin | IA | -20.9 | 7.2 | Horse | Murphy et al. 2013 | |
| Not Available | Chilannygtakh | Minusinsk Basin | IA | -19.0 | 6.2 | Goat | Svyatko et al. 2007 | * |
| Not Available | Chilannygtakh | Minusinsk Basin | IA | -21.4 | 4.1 | Horse | Svyatko et al. 2007 | * |
| Not Available | Itkol I | Minusinsk Basin | MBA | -21.1 | 4.4 | Horse | Svyatko et al. 2007 | * |
| Not Available | Kamenniy Ostrov | Minusinsk Basin | LBA/EIA | -20.3 | 7.2 | Horse | Svyatko et al. 2007 | * |
| Not Available | Kamenniy Ostrov | Minusinsk Basin | LBA/EIA | -19.1 | 5.8 | Horse, immature | Svyatko et al. 2007 | * |
| Not Available | Sukhanikha | Minusinsk Basin | EBA | -20.7 | 3.8 | Goat | Svyatko et al. 2007 | * |
| Not Available | Sukhanikha | Minusinsk Basin | EBA | -18.8 | 6.8 | Goat, immature | Svyatko et al. 2007 | * |
| Not Available | Ai-Dai | Minusinsk Basin | IA | -20.8 | 6.3 | Cattle | Svyatko et al. 2007 | * |
| Not Available | Aymyrlyg | Minusinsk Basin | IA | -19.1 | 7.1 | Sheep/goat | Murphy et al. 2013 | |
| Not Available | Aymyrlyg | Minusinsk Basin | IA | -19.0 | 9.4 | Sheep/goat | Murphy et al. 2013 | |
| Not Available | Bolshaya Erba II | Minusinsk Basin | IA | -19.9 | 6.0 | Sheep | Svyatko et al. 2007 | * |
| Not Available | Itkol III | Minusinsk Basin | LBA | -18.3 | 5.1 | Sheep | Svyatko et al. 2007 | * |
| Not Available | Sukhanikha | Minusinsk Basin | EBA | -20.8 | 3.8 | Ovicaprid | Svyatko et al. 2007 | * |

| | | | | | | | | |
|---|---|---|---|---|---|---|---|---|
| Not Available | Sukhanikha | Minusinsk Basin | EBA | -20.8 | 3.8 | sheep/goat | Svyatko et al. 2007 | * |
| Not Available | Sukhanikha | Minusinsk Basin | EBA | -20.7 | 3.8 | sheep/goat | Svyatko et al. 2007 | * |
| Not Available | Ulaanzuukh | C. Mongolia | MBA | -17.1 | 12.3 | Homo sapiens | Fenner et al 2014 | |
| Not Available | Ulaanzuukh | C. Mongolia | MBA | -17.4 | 12.5 | Homo sapiens | Fenner et al 2014 | |
| Not Available | Ulaanzuukh | C. Mongolia | MBA | -16.9 | 12.4 | Homo sapiens | Fenner et al 2014 | |
| Not Available | Ulaanzuukh | C. Mongolia | MBA | -18.3 | 12.6 | Homo sapiens | Fenner et al 2014 | |
| Not Available | Ulaanzuukh | C. Mongolia | MBA | -16.5 | 13.8 | Homo sapiens | Fenner et al 2014 | |
| Not Available | Ulaanzuukh | C. Mongolia | MBA | -17.1 | 12.0 | Homo sapiens | Fenner et al 2014 | |
| Not Available | Ulaanzuukh | C. Mongolia | MBA | -16.5 | 12.9 | Homo sapiens | Fenner et al 2014 | |
| Not Available | Ulaanzuukh | C. Mongolia | MBA | -16.3 | 13.6 | Homo sapiens | Fenner et al 2014 | |
| Not Available | Ulaanzuukh | C. Mongolia | MBA | -16.8 | 13.0 | Homo sapiens | Fenner et al 2014 | |
| Not Available | Ulaanzuukh | C. Mongolia | MBA | -16.6 | 10.8 | Homo sapiens | Fenner et al 2014 | |
| Not Available | Ulaanzuukh | C. Mongolia | MBA | -17.7 | 10.9 | Homo sapiens | Fenner et al 2014 | |
| Not Available | Ulaanzuukh | C. Mongolia | MBA | -16.0 | 11.9 | Homo sapiens | Fenner et al 2014 | |
| Not Available | Ulaanzuukh | C. Mongolia | MBA | -18.5 | 10.6 | Homo sapiens | Fenner et al 2014 | |
| EX 07.01 | BGC | C. Mongolia | LBA/EIA | -16.6 | 14.0 | Homo sapiens | Machicek 2010; Machicek 2011 | |
| EX 07.07 | BGC | C. Mongolia | LBA/EIA | -17.8 | 9.0 | Homo sapiens | Machicek 2010; Machicek 2011 | |
| EX 07.19B | BGC | C. Mongolia | LBA/EIA | -17.3 | 13.3 | Homo sapiens | Machicek 2010; Machicek 2011 | |
| EX 8.17 | BGC | C. Mongolia | LBA/EIA | -17.5 | 12.9 | Homo sapiens | Machicek 2010; Machicek 2011 | |
| B60 | Daram uul | C. Mongolia | LBA/EIA | -17.2 | 11.8 | Homo sapiens | Yoneda et al. 2016 | |
| D038 | Tevsh | C. Mongolia | LBA/EIA | -17.3 | 13.9 | Homo sapiens | Yoneda et al. 2016 | |
| No. 4 | Tevsh | C. Mongolia | LBA/EIA | -16.5 | 14.5 | Homo sapiens | Yoneda et al. 2016 | |
| No. 1 | Tevsh | C. Mongolia | LBA/EIA | -15.4 | 15.8 | Homo sapiens | Yoneda et al. 2016 | |
| No. 4 | Daram uul | C. Mongolia | LBA/EIA | -17.8 | 12.9 | Homo sapiens | Yoneda et al. 2016 | |
| No. 3 | Daram uul | C. Mongolia | LBA/EIA | -17.0 | 9.8 | Homo sapiens | Yoneda et al. 2016 | |
| EX 08.25 | BGC | C. Mongolia | LIA | -15.2 | 14.0 | Homo sapiens | Machicek 2010; Machicek 2011 | |
| EX 03.03 | BGC | C. Mongolia | LIA | -14.9 | 13.0 | Homo sapiens | Machicek 2010; Machicek 2011 | |

| | | | | | | | |
|---|---|---|---|---|---|---|---|
| EX 03.07 | BGC | C. Mongolia | LIA | -13.8 | 13.7 | Homo sapiens | Machicek 2010; Machicek 2011 |
| EX 04.01 | BGC | C. Mongolia | LIA | -15.1 | 13.3 | Homo sapiens | Machicek 2010; Machicek 2011 |
| EX 04.02 | BGC | C. Mongolia | LIA | -16.5 | 12.8 | Homo sapiens | Machicek 2010; Machicek 2011 |
| EX 04.10 | BGC | C. Mongolia | LIA | -15.7 | 14.7 | Homo sapiens | Machicek 2010; Machicek 2011 |
| EX 05.02 | BGC | C. Mongolia | LIA | -15.2 | 13.5 | Homo sapiens | Machicek 2010; Machicek 2011 |
| EX 06.08 | BGC | C. Mongolia | LIA | -14.6 | 13.1 | Homo sapiens | Machicek 2010; Machicek 2011 |
| EX 06.09 | BGC | C. Mongolia | LIA | -16.4 | 12.2 | Homo sapiens | Machicek 2010; Machicek 2011 |
| EX 07.16 | BGC | C. Mongolia | LIA | -17.0 | 13.5 | Homo sapiens | Machicek 2010; Machicek 2011 |
| EX 07.25b | BGC | C. Mongolia | LIA | -16.1 | 13.4 | Homo sapiens | Machicek 2010; Machicek 2011 |
| EX 08.02 | BGC | C. Mongolia | LIA | -16.3 | 14.8 | Homo sapiens | Machicek 2010; Machicek 2011 |
| EX 08.03 | BGC | C. Mongolia | LIA | -14.8 | 14.8 | Homo sapiens | Machicek 2010; Machicek 2011 |
| EX 08.04a | BGC | C. Mongolia | LIA | -15.7 | 14.9 | Homo sapiens | Machicek 2010; Machicek 2011 |
| EX 08.04b | BGC | C. Mongolia | LIA | -17.3 | 13.6 | Homo sapiens | Machicek 2010; Machicek 2011 |
| EX 08.05 | BGC | C. Mongolia | LIA | -15.0 | 15.5 | Homo sapiens | Machicek 2010; Machicek 2011 |
| EX 08.06 | BGC | C. Mongolia | LIA | -16.5 | 12.8 | Homo sapiens | Machicek 2010; Machicek 2011 |
| EX 08.08 | BGC | C. Mongolia | LIA | -14.7 | 13.4 | Homo sapiens | Machicek 2010; Machicek 2011 |
| EX 08.13 | BGC | C. Mongolia | LIA | -14.3 | 13.6 | Homo sapiens | Machicek 2010; Machicek 2011 |
| EX 08.19 | BGC | C. Mongolia | LIA | -16.3 | 13.2 | Homo sapiens | Machicek 2010; Machicek 2011 |
| EX 05.04 | BGC | C. Mongolia | LIA | -15.2 | 14.2 | Homo sapiens | Machicek 2010; Machicek 2011 |
| EX 05.05 | BGC | C. Mongolia | LIA | -15.4 | 13.7 | Homo sapiens | Machicek 2010; Machicek 2011 |
| BGC0302FB | BGC | C. Mongolia | LIA | -16.0 | 10.5 | Ovis/Capra | Machicek 2010; Machicek 2011 |
| BGC0802FB | BGC | C. Mongolia | LIA | -13.2 | 12.6 | Ovis/Capra | Machicek 2010; Machicek 2011 |
| BGC0803FB | BGC | C. Mongolia | LIA | -15.4 | 11.6 | Bos sp. | Machicek 2010; Machicek 2011 |
| BGC0804FB | BGC | C. Mongolia | LIA | -16.8 | 11.4 | Bos sp. | Machicek 2010; Machicek 2011 |
| BGC0813FB | BGC | C. Mongolia | LIA | -16.5 | 9.4 | Ovis/Capra | Machicek 2010; Machicek 2011 |
| BGC 128 | BGC | C. Mongolia | LIA | -16.9 | 11.2 | Ovis | Makarewicz 2015 |
| BGC 128 | BGC | C. Mongolia | LIA | -15.8 | 11.3 | Ovis/Capra | Makarewicz 2015 |

| | | | | | | | |
|---|---|---|---|---|---|---|---|
| BGC 128 | BGC | C. Mongolia | LIA | -16.7 | 11.0 | Ovis | Makarewicz 2015 |
| BGC 128 | BGC | C. Mongolia | LIA | -16.4 | 11.3 | Ovis/Capra | Makarewicz 2015 |
| BGC 576 | BGC | C. Mongolia | LIA | -18.1 | 9.1 | Capra | Makarewicz 2015 |
| BGC 576 | BGC | C. Mongolia | LIA | -16.2 | 10.2 | Ovis/Capra | Makarewicz 2015 |
| BGC 576 | BGC | C. Mongolia | LIA | -16.9 | 9.5 | Ovis | Makarewicz 2015 |
| BGC 576 | BGC | C. Mongolia | LIA | -17.3 | 7.3 | Ovis/Capra | Makarewicz 2015 |
| BGC0806FB | BGC | C. Mongolia | LIA | -17.2 | 11.4 | Ovis/Capra | Machicek 2010; Machicek 2011 |
| D010 | Daram uul | C. Mongolia | LBA/EIA | -18.9 | 7.7 | Bos | Yoneda et al. 2016 |
| D042 | Daram uul | C. Mongolia | LBA/EIA | -20.0 | 5.1 | equus | Yoneda et al. 2016 |
| D012 | Daram uul | C. Mongolia | LBA/EIA | -19.3 | 5.4 | equus | Yoneda et al. 2016 |
| D048 | Daram uul | C. Mongolia | LBA/EIA | -17.8 | 8.0 | Bos | Yoneda et al. 2016 |
| D047 | Daram uul | C. Mongolia | LBA/EIA | -18.4 | 6.9 | equus | Yoneda et al. 2016 |
| D021 | Daram uul | C. Mongolia | LBA/EIA | -16.5 | 7.5 | Bos | Yoneda et al. 2016 |
| EX 97.1 | Egiin Gol | N. Mongolia | LBA/EIA | -17.6 | 11.2 | Homo sapiens | Machicek 2010; Machicek 2011 |
| EG EX 00.4 | Egiin Gol | N. Mongolia | LBA/EIA | -17.7 | 11.1 | Homo sapiens | Machicek 2010; Machicek 2011 |
| EG EX 00.6a | Egiin Gol | N. Mongolia | LBA/EIA | -18.0 | 11.6 | Homo sapiens | Machicek 2010; Machicek 2011 |
| EG EX 00.9 | Egiin Gol | N. Mongolia | LBA/EIA | -17.2 | 12.1 | Homo sapiens | Machicek 2010; Machicek 2011 |
| EG EX 00.12 | Egiin Gol | N. Mongolia | LBA/EIA | -18.0 | 11.8 | Homo sapiens | Machicek 2010; Machicek 2011 |
| EX 00.14 | Egiin Gol | N. Mongolia | LIA | -14.3 | 12.9 | Homo sapiens | Machicek 2010; Machicek 2011 |
| EG BUR 45 | Egiin Gol | N. Mongolia | LIA | -16.1 | 12.3 | Homo sapiens | Machicek 2010; Machicek 2011 |
| EG BUR 46a | Egiin Gol | N. Mongolia | LIA | -17.5 | 13.3 | Homo sapiens | Machicek 2010; Machicek 2011 |
| EX 99.08 | Egiin Gol | N. Mongolia | LIA | -16.9 | 14.0 | Homo sapiens | Machicek 2010; Machicek 2011 |
| EGS T-12 | Egiin Gol | N. Mongolia | LIA | -16.1 | 12.4 | Homo sapiens | Machicek 2010; Machicek 2011 |
| BT T-23 | Egiin Gol | N. Mongolia | LIA | -14.6 | 13.1 | Homo sapiens | Machicek 2010; Machicek 2011 |
| EG T-27 | Egiin Gol | N. Mongolia | LIA | -16.4 | 12.5 | Homo sapiens | Machicek 2010; Machicek 2011 |
| BT T-35 | Egiin Gol | N. Mongolia | LIA | -14.8 | 12.1 | Homo sapiens | Machicek 2010; Machicek 2011 |
| BT T-37a | Egiin Gol | N. Mongolia | LIA | -17.5 | 11.7 | Homo sapiens | Machicek 2010; Machicek 2011 |

| | | | | | | | |
|---|---|---|---|---|---|---|---|
| BT T-48 | Egiin Gol | N. Mongolia | LIA | -16.3 | 11.7 | Homo sapiens | Machicek 2010; Machicek 2011 |
| BT T-51 | Egiin Gol | N. Mongolia | LIA | -16.0 | 11.9 | Homo sapiens | Machicek 2010; Machicek 2011 |
| BT T-52 | Egiin Gol | N. Mongolia | LIA | -15.1 | 11.5 | Homo sapiens | Machicek 2010; Machicek 2011 |
| BT T-54 | Egiin Gol | N. Mongolia | LIA | -16.1 | 12.0 | Homo sapiens | Machicek 2010; Machicek 2011 |
| BT T-56 | Egiin Gol | N. Mongolia | LIA | -15.8 | 12.9 | Homo sapiens | Machicek 2010; Machicek 2011 |
| BT T-60 | Egiin Gol | N. Mongolia | LIA | -17.1 | 12.3 | Homo sapiens | Machicek 2010; Machicek 2011 |
| EGT-64 | Egiin Gol | N. Mongolia | LIA | -14.5 | 11.5 | Homo sapiens | Machicek 2010; Machicek 2011 |
| BT T-68 | Egiin Gol | N. Mongolia | LIA | -16.0 | 11.7 | Homo sapiens | Machicek 2010; Machicek 2011 |
| EGT-72 | Egiin Gol | N. Mongolia | LIA | -16.7 | 12.6 | Homo sapiens | Machicek 2010; Machicek 2011 |
| EGIT - 74 | Egiin Gol | N. Mongolia | LIA | -16.4 | 11.1 | Homo sapiens | Machicek 2010; Machicek 2011 |
| BT T-75 | Egiin Gol | N. Mongolia | LIA | -17.0 | 12.1 | Homo sapiens | Machicek 2010; Machicek 2011 |
| EGT-78 | Egiin Gol | N. Mongolia | LIA | -16.4 | 12.1 | Homo sapiens | Machicek 2010; Machicek 2011 |
| BT T-84 | Egiin Gol | N. Mongolia | LIA | -15.9 | 10.9 | Homo sapiens | Machicek 2010; Machicek 2011 |
| BT T-88 | Egiin Gol | N. Mongolia | LIA | -15.7 | 11.9 | Homo sapiens | Machicek 2010; Machicek 2011 |
| EGT-92 | Egiin Gol | N. Mongolia | LIA | -15.4 | 10.9 | Homo sapiens | Machicek 2010; Machicek 2011 |
| EGT12FB | Egiin Gol | N. Mongolia | LIA | -19.4 | 7.2 | Ovis/Capra | Machicek 2010; Machicek 2011 |
| EGT18FB | Egiin Gol | N. Mongolia | LIA | -18.2 | 8.3 | Bos | Machicek 2010; Machicek 2011 |
| EFT32FB | Egiin Gol | N. Mongolia | LIA | -18.3 | 8.0 | Bos | Machicek 2010; Machicek 2011 |
| EFT37FB | Egiin Gol | N. Mongolia | LIA | -18.0 | 8.7 | Bos | Machicek 2010; Machicek 2011 |
| EGT48FB | Egiin Gol | N. Mongolia | LIA | -17.7 | 8.3 | Bos | Machicek 2010; Machicek 2011 |
| EFT59FB | Egiin Gol | N. Mongolia | LIA | -18.0 | 8.4 | Bos | Machicek 2010; Machicek 2011 |
| EGT67FB | Egiin Gol | N. Mongolia | LIA | -19.0 | 7.3 | Bos | Machicek 2010; Machicek 2011 |
| EGT77FB | Egiin Gol | N. Mongolia | LIA | -18.0 | 8.6 | Ovis/Capra | Machicek 2010; Machicek 2011 |
| EFT81FB | Egiin Gol | N. Mongolia | LIA | -18.4 | 8.7 | Ovis/Capra | Machicek 2010; Machicek 2011 |
| EFT81FB | Egiin Gol | N. Mongolia | LIA | -18.9 | 7.9 | Ovis/Capra | Machicek 2010; Machicek 2011 |
| GM_028 | Oi-Dzailau VII | S.E. Kazakhstan | LBA2 | -18.0 | 12.8 | Homo sapiens | Motuzaite Matuzeviciute et al. 2015 |
| GM_029 | Oi-Dzailau VII | S.E. Kazakhstan | LBA2 | -18.5 | 13.8 | Homo sapiens | Motuzaite Matuzeviciute et al. 2015 |

| | | | | | | | |
|---|---|---|---|---|---|---|---|
| GM_025 | Oi-Dzailau VII | S.E. Kazakhstan | LBA2 | -16.9 | 13.9 | Homo sapiens | Motuzaite Matuzeviciute et al. 2015 |
| GM_033 | Oi-Dzailau VII | S.E. Kazakhstan | LBA2 | -16.1 | 13.1 | Homo sapiens | Motuzaite Matuzeviciute et al. 2015 |
| GM_035 | Oi-Dzailau VII | S.E. Kazakhstan | LBA2 | -14.8 | 14.5 | Homo sapiens | Motuzaite Matuzeviciute et al. 2015 |
| GM_034 | Oi-Dzailau VII | S.E. Kazakhstan | LBA2 | -14.4 | 13.2 | Homo sapiens | Motuzaite Matuzeviciute et al. 2015 |
| GM_020 | Oi-Dzailau VII | S.E. Kazakhstan | LBA2 | -14.5 | 13.4 | Homo sapiens | Motuzaite Matuzeviciute et al. 2015 |
| GM_026 | Oi-Dzailau VII | S.E. Kazakhstan | LBA2 | -13.7 | 14.6 | Homo sapiens | Motuzaite Matuzeviciute et al. 2015 |
| GM_024 | Oi-Dzailau VII | S.E. Kazakhstan | LBA2 | -13.3 | 14.7 | Homo sapiens | Motuzaite Matuzeviciute et al. 2015 |
| GM_027 | Oi-Dzailau VII | S.E. Kazakhstan | LBA2 | -14.1 | 14.3 | Homo sapiens | Motuzaite Matuzeviciute et al. 2015 |
| GM_022 | Oi-Dzailau VII | S.E. Kazakhstan | LBA2 | -16.8 | 13.7 | Homo sapiens | Motuzaite Matuzeviciute et al. 2015 |
| GM_036 | Kyzyl Bulak-1 | S.E. Kazakhstan | LBA1 | -16.3 | 12.8 | Homo sapiens | Motuzaite Matuzeviciute et al. 2015 |
| GM_031 | Kyzyl Bulak-1 | S.E. Kazakhstan | LBA1 | -18.4 | 10.2 | Homo sapiens | Motuzaite Matuzeviciute et al. 2015 |
| GM_021 | Kyzyl Bulak-1 | S.E. Kazakhstan | LBA1 | -19.0 | 9.7 | Homo sapiens | Motuzaite Matuzeviciute et al. 2015 |
| GM_030 | Kyzyl Bulak-1 | S.E. Kazakhstan | LBA1 | -17.6 | 10.2 | Homo sapiens | Motuzaite Matuzeviciute et al. 2015 |
| GM_049 | Karatuma | S.E. Kazakhstan | EIA | -15.0 | 13.4 | Homo sapiens | Motuzaite Matuzeviciute et al. 2015 |
| GM_050 | Karatuma | S.E. Kazakhstan | EIA | -15.1 | 14.8 | Homo sapiens | Motuzaite Matuzeviciute et al. 2015 |
| GM_051 | Karatuma | S.E. Kazakhstan | EIA | -16.0 | 11.7 | Homo sapiens | Motuzaite Matuzeviciute et al. 2015 |
| GM_053 | Karatuma | S.E. Kazakhstan | EIA | -16.3 | 12.1 | Homo sapiens | Motuzaite Matuzeviciute et al. 2015 |
| GM_054 | Karatuma | S.E. Kazakhstan | EIA | -15.5 | 14.2 | Homo sapiens | Motuzaite Matuzeviciute et al. 2015 |
| GM_055 | Karatuma | S.E. Kazakhstan | EIA | -15.2 | 11.1 | Homo sapiens | Motuzaite Matuzeviciute et al. 2015 |
| GM_058 | Karatuma | S.E. Kazakhstan | EIA | -16.0 | 12.0 | Homo sapiens | Motuzaite Matuzeviciute et al. 2015 |
| GM_059 | Karatuma | S.E. Kazakhstan | EIA | -14.5 | 12.4 | Homo sapiens | Motuzaite Matuzeviciute et al. 2015 |
| GM_062 | Karatuma | S.E. Kazakhstan | EIA | -14.9 | 9.0 | Homo sapiens | Motuzaite Matuzeviciute et al. 2015 |
| GM_063 | Karatuma | S.E. Kazakhstan | EIA | -15.0 | 13.5 | Homo sapiens | Motuzaite Matuzeviciute et al. 2015 |
| GM_065 | Karatuma | S.E. Kazakhstan | EIA | -16.2 | 12.1 | Homo sapiens | Motuzaite Matuzeviciute et al. 2015 |
| GM_068 | Karatuma | S.E. Kazakhstan | EIA | -14.4 | 13.0 | Homo sapiens | Motuzaite Matuzeviciute et al. 2015 |
| GM_070 | Karatuma | S.E. Kazakhstan | EIA | -16.2 | 12.1 | Homo sapiens | Motuzaite Matuzeviciute et al. 2015 |
| GM_072 | Karatuma | S.E. Kazakhstan | EIA | -13.2 | 12.4 | Homo sapiens | Motuzaite Matuzeviciute et al. 2015 |

| | | | | | | | |
|---|---|---|---|---|---|---|---|
| GM_074 | Karatuma | S.E. Kazakhstan | EIA | -12.9 | 17.2 | Homo sapiens | Motuzaite Matuzeviciute et al. 2015 |
| GM_075 | Karatuma | S.E. Kazakhstan | EIA | -16.9 | 10.7 | Homo sapiens | Motuzaite Matuzeviciute et al. 2015 |
| GM_076 | Karatuma | S.E. Kazakhstan | EIA | -14.7 | 13.5 | Homo sapiens | Motuzaite Matuzeviciute et al. 2015 |
| GM_077 | Karatuma | S.E. Kazakhstan | EIA | -16.5 | 12.5 | Homo sapiens | Motuzaite Matuzeviciute et al. 2015 |
| GM_079 | Karatuma | S.E. Kazakhstan | EIA | -15.4 | 12.7 | Homo sapiens | Motuzaite Matuzeviciute et al. 2015 |
| GM_080 | Karatuma | S.E. Kazakhstan | EIA | -16.0 | 12.2 | Homo sapiens | Motuzaite Matuzeviciute et al. 2015 |
| GM_082 | Karatuma | S.E. Kazakhstan | EIA | -15.9 | 12.3 | Homo sapiens | Motuzaite Matuzeviciute et al. 2015 |
| GM_083 | Karatuma | S.E. Kazakhstan | EIA | -15.8 | 12.7 | Homo sapiens | Motuzaite Matuzeviciute et al. 2015 |
| GM_086 | Karatuma | S.E. Kazakhstan | EIA | -16.1 | 13.4 | Homo sapiens | Motuzaite Matuzeviciute et al. 2015 |
| GM_087 | Karatuma | S.E. Kazakhstan | EIA | -16.3 | 10.7 | Homo sapiens | Motuzaite Matuzeviciute et al. 2015 |
| GM_090 | Karatuma | S.E. Kazakhstan | EIA | -16.8 | 12.9 | Homo sapiens | Motuzaite Matuzeviciute et al. 2015 |
| GM_092 | Karatuma | S.E. Kazakhstan | EIA | -15.1 | 12.3 | Homo sapiens | Motuzaite Matuzeviciute et al. 2015 |
| GM_095 | Karatuma | S.E. Kazakhstan | EIA | -15.2 | 13.3 | Homo sapiens | Motuzaite Matuzeviciute et al. 2015 |
| GM_098 | Karatuma | S.E. Kazakhstan | EIA | -13.8 | 13.2 | Homo sapiens | Motuzaite Matuzeviciute et al. 2015 |
| GM_099 | Karatuma | S.E. Kazakhstan | EIA | -15.8 | 11.7 | Homo sapiens | Motuzaite Matuzeviciute et al. 2015 |
| GM_109 | Karatuma | S.E. Kazakhstan | EIA | -17.0 | 9.0 | Homo sapiens | Motuzaite Matuzeviciute et al. 2015 |
| GM_077B | Karatuma | S.E. Kazakhstan | EIA | -14.0 | 12.6 | Homo sapiens | Motuzaite Matuzeviciute et al. 2015 |
| GM_077A | Karatuma | S.E. Kazakhstan | EIA | -14.8 | 13.5 | Homo sapiens | Motuzaite Matuzeviciute et al. 2015 |
| GM_F78 | Karatuma | S.E. Kazakhstan | EIA | -16.5 | 11.3 | Homo sapiens | Motuzaite Matuzeviciute et al. 2015 |
| GM_148 | Karatuma | S.E. Kazakhstan | EIA | -15.1 | 12.5 | Homo sapiens | Motuzaite Matuzeviciute et al. 2015 |
| GM_155 | Karatuma | S.E. Kazakhstan | EIA | -13.5 | 11.9 | Homo sapiens | Motuzaite Matuzeviciute et al. 2015 |
| GM_156 | Karatuma | S.E. Kazakhstan | EIA | -15.7 | 11.4 | Homo sapiens | Motuzaite Matuzeviciute et al. 2015 |
| GM_157 | Karatuma | S.E. Kazakhstan | EIA | -16.4 | 10.6 | Homo sapiens | Motuzaite Matuzeviciute et al. 2015 |
| GM_082 | Karatuma | S.E. Kazakhstan | EIA | -16.8 | 13.7 | Homo sapiens | Motuzaite Matuzeviciute et al. 2015 |
| GM_158 | Karatuma | S.E. Kazakhstan | EIA | -15.2 | 8.8 | Homo sapiens | Motuzaite Matuzeviciute et al. 2015 |
| GM_046 | Kargaly-I | S.E. Kazakhstan | EIA | -13.6 | 12.4 | Homo sapiens | Motuzaite Matuzeviciute et al. 2015 |
| GM_047 | Kargaly-I | S.E. Kazakhstan | EIA | -15.5 | 12.5 | Homo sapiens | Motuzaite Matuzeviciute et al. 2015 |

| | | | | | | | |
|---|---|---|---|---|---|---|---|
| GM_044 | Kargaly-I | S.E. Kazakhstan | EIA | -14.2 | 10.4 | Homo sapiens | Motuzaite Matuzeviciute et al. 2015 |
| GM_045 | Kargaly-I | S.E. Kazakhstan | EIA | -15.0 | 12.0 | Homo sapiens | Motuzaite Matuzeviciute et al. 2015 |
| GM_037 | Alatau-1 | S.E. Kazakhstan | EIA | -16.2 | 10.9 | Homo sapiens | Motuzaite Matuzeviciute et al. 2015 |
| GM_038 | Alatau-1 | S.E. Kazakhstan | EIA | -17.3 | 10.0 | Homo sapiens | Motuzaite Matuzeviciute et al. 2015 |
| GM_039 | Alatau-1 | S.E. Kazakhstan | EIA | -15.9 | 11.6 | Homo sapiens | Motuzaite Matuzeviciute et al. 2015 |
| GM_040 | Kamenka | S.E. Kazakhstan | EIA | -16.0 | 10.3 | Homo sapiens | Motuzaite Matuzeviciute et al. 2015 |
| GM_042 | Kamenka | S.E. Kazakhstan | EIA | -15.3 | 9.7 | Homo sapiens | Motuzaite Matuzeviciute et al. 2015 |
| GM_041 | Kamenka | S.E. Kazakhstan | EIA | -15.5 | 12.3 | Homo sapiens | Motuzaite Matuzeviciute et al. 2015 |
| GM_F76 | Karatuma | S.E. Kazakhstan | EIA | -16.7 | 10.7 | Caprine | Motuzaite Matuzeviciute et al. 2015 |
| GM_F77 | Karatuma | S.E. Kazakhstan | EIA | -16.8 | 10.7 | Caprine | Motuzaite Matuzeviciute et al. 2015 |
| GM_F82 | Kyzyl-Bulak | S.E. Kazakhstan | MBA | -19.0 | 9.6 | Caprine | Motuzaite Matuzeviciute et al. 2015 |
| GM_F78 | Karatuma | S.E. Kazakhstan | EIA | -17.0 | 9.9 | Caprine | Motuzaite Matuzeviciute et al. 2015 |
| GM_F04 | Butakty | S.E. Kazakhstan | Med. | -18.4 | 7.4 | Caprine | Motuzaite Matuzeviciute et al. 2015 |
| GM_F023 | Oi-Dzailau VII | S.E. Kazakhstan | MBA | -19.4 | 7.6 | Caprine | Motuzaite Matuzeviciute et al. 2015 |
| KZ062 | Akimbek | C. Kazakhstan | LBA | -18.4 | 14.4 | Homo sapiens | Lightfoot et al 2014 |
| KZ063 | Akimbek | C. Kazakhstan | LBA | -18.1 | 14.1 | Homo sapiens | Lightfoot et al 2014 |
| KZ064 | Akimbek | C. Kazakhstan | LBA | -18.2 | 13.3 | Homo sapiens | Lightfoot et al 2014 |
| KZ065 | Akimbek | C. Kazakhstan | LBA | -18.4 | 12.9 | Homo sapiens | Lightfoot et al 2014 |
| KZ087 | Akimbek | C. Kazakhstan | LBA | -18.5 | 15.5 | Homo sapiens | Lightfoot et al 2014 |
| KZ050 | Aschisu | C. Kazakhstan | LBA | -18.9 | 12.4 | Homo sapiens | Lightfoot et al 2014 |
| KZ052 | Aschisu | C. Kazakhstan | LBA | -17.2 | 14.2 | Homo sapiens | Lightfoot et al 2014 |
| KZ054 | Aschisu | C. Kazakhstan | LBA | -18.7 | 11.9 | Homo sapiens | Lightfoot et al 2014 |
| KZ055 | Aschisu | C. Kazakhstan | LBA | -18.6 | 11.7 | Homo sapiens | Lightfoot et al 2014 |
| KZ056 | Aschisu | C. Kazakhstan | LBA | -19.1 | 11.5 | Homo sapiens | Lightfoot et al 2014 |
| KZ057 | Aschisu | C. Kazakhstan | LBA | -18.4 | 12.8 | Homo sapiens | Lightfoot et al 2014 |
| KZ058 | Aschisu | C. Kazakhstan | LBA | -18.8 | 11.7 | Homo sapiens | Lightfoot et al 2014 |
| KZ059 | Aschisu | C. Kazakhstan | LBA | -18.4 | 12.5 | Homo sapiens | Lightfoot et al 2014 |

| | | | | | | | |
|---|---|---|---|---|---|---|---|
| KZ076 | Aschisu | C. Kazakhstan | LBA | -17.8 | 13.6 | Homo sapiens | Lightfoot et al 2014 |
| KZ051 | Ayapbergen | C. Kazakhstan | LBA | -18.5 | 12.3 | Homo sapiens | Lightfoot et al 2014 |
| KZ042 | Dariya | C. Kazakhstan | LBA | -18.0 | 14.3 | Homo sapiens | Lightfoot et al 2014 |
| KZ043 | Dariya | C. Kazakhstan | LBA | -18.1 | 14.2 | Homo sapiens | Lightfoot et al 2014 |
| KZ044 | Dariya | C. Kazakhstan | LBA | -17.7 | 14.2 | Homo sapiens | Lightfoot et al 2014 |
| KZ102 | Kairakty | C. Kazakhstan | LBA | -19.0 | 11.6 | Homo sapiens | Lightfoot et al 2014 |
| KZ061 | Kopa-I | C. Kazakhstan | LBA | -18.3 | 12.5 | Homo sapiens | Lightfoot et al 2014 |
| KZ077 | Kyzylkol | C. Kazakhstan | LBA | -18.2 | 14.0 | Homo sapiens | Lightfoot et al 2014 |
| KZ078 | Kyzylkol | C. Kazakhstan | LBA | -18.3 | 13.8 | Homo sapiens | Lightfoot et al 2014 |
| KZ079 | Kyzylkol | C. Kazakhstan | LBA | -18.9 | 13.1 | Homo sapiens | Lightfoot et al 2014 |
| KZ080 | Kyzylkol | C. Kazakhstan | LBA | -18.4 | 15.1 | Homo sapiens | Lightfoot et al 2014 |
| KZ081 | Kyzylkol | C. Kazakhstan | LBA | -18.8 | 13.4 | Homo sapiens | Lightfoot et al 2014 |
| KZ082 | Kyzylkol | C. Kazakhstan | LBA | -18.9 | 13.0 | Homo sapiens | Lightfoot et al 2014 |
| KZ083 | Kyzylkol | C. Kazakhstan | LBA | -19.2 | 13.2 | Homo sapiens | Lightfoot et al 2014 |
| KZ084 | Kyzylkol | C. Kazakhstan | LBA | -18.4 | 14.4 | Homo sapiens | Lightfoot et al 2014 |
| KZ027 | Nurataldy | C. Kazakhstan | LBA | -18.6 | 14.3 | Homo sapiens | Lightfoot et al 2014 |
| KZ045 | Tashik | C. Kazakhstan | LBA | -18.7 | 12.6 | Homo sapiens | Lightfoot et al 2014 |
| KZ046 | Tashik | C. Kazakhstan | LBA | -18.7 | 13.1 | Homo sapiens | Lightfoot et al 2014 |
| KZ047 | Tashik | C. Kazakhstan | LBA | -18.6 | 13.8 | Homo sapiens | Lightfoot et al 2014 |
| KZ048 | Tashik | C. Kazakhstan | LBA | -18.5 | 13.3 | Homo sapiens | Lightfoot et al 2014 |
| KZ049 | Tashik | C. Kazakhstan | LBA | -18.5 | 13.0 | Homo sapiens | Lightfoot et al 2014 |
| KZ067 | Tashik | C. Kazakhstan | LBA | -18.2 | 13.6 | Homo sapiens | Lightfoot et al 2014 |
| KZ068 | Tashik | C. Kazakhstan | LBA | -18.9 | 12.7 | Homo sapiens | Lightfoot et al 2014 |
| KZ069 | Tashik | C. Kazakhstan | LBA | -18.1 | 14.7 | Homo sapiens | Lightfoot et al 2014 |
| KZ070 | Tashik | C. Kazakhstan | LBA | -18.3 | 14.0 | Homo sapiens | Lightfoot et al 2014 |
| KZ071 | Tashik | C. Kazakhstan | LBA | -18.2 | 14.9 | Homo sapiens | Lightfoot et al 2014 |
| KZ072 | Tashik | C. Kazakhstan | LBA | -18.7 | 12.6 | Homo sapiens | Lightfoot et al 2014 |

| | | | | | | | |
|---|---|---|---|---|---|---|---|
| KZ073 | Tashik | C. Kazakhstan | LBA | -18.2 | 13.1 | Homo sapiens | Lightfoot et al 2014 |
| KZ088 | Tashik | C. Kazakhstan | LBA | -18.7 | 12.9 | Homo sapiens | Lightfoot et al 2014 |
| KZ089 | Tashik | C. Kazakhstan | LBA | -18.0 | 12.9 | Homo sapiens | Lightfoot et al 2014 |
| KZ090 | Tashik | C. Kazakhstan | LBA | -18.3 | 12.5 | Homo sapiens | Lightfoot et al 2014 |
| KZ066 | Tasyrbai | C. Kazakhstan | LBA | -18.6 | 11.4 | Homo sapiens | Lightfoot et al 2014 |
| KZ093 | Temirkash | C. Kazakhstan | LBA | -18.8 | 12.7 | Homo sapiens | Lightfoot et al 2014 |
| KZ094 | Temirkash | C. Kazakhstan | LBA | -17.6 | 13.3 | Homo sapiens | Lightfoot et al 2014 |
| KZ104 | Kent | C. Kazakhstan | FBA | -16.0 | 13.5 | Homo sapiens | Lightfoot et al 2014 |
| KZ092 | Kyzyl | C. Kazakhstan | FBA | -16.8 | 13.0 | Homo sapiens | Lightfoot et al 2014 |
| KZ097 | Karatugai | C. Kazakhstan | FBA | -17.7 | 14.0 | Homo sapiens | Lightfoot et al 2014 |
| KZ098 | Karatugai | C. Kazakhstan | FBA | -17.8 | 13.9 | Homo sapiens | Lightfoot et al 2014 |
| KZ099 | Karatugai | C. Kazakhstan | FBA | -18.1 | 14.3 | Homo sapiens | Lightfoot et al 2014 |
| KZ100 | Karatugai | C. Kazakhstan | FBA | -18.8 | 12.7 | Homo sapiens | Lightfoot et al 2014 |
| KZ096 | Karatugai | C. Kazakhstan | FBA | -18.4 | 12.8 | Homo sapiens | Lightfoot et al 2014 |
| KZ075 | Kudryavaya Sopka-1 | C. Kazakhstan | FBA | -18.1 | 15.3 | Homo sapiens | Lightfoot et al 2014 |
| KZ085 | Kudryavaya Sopka-1 | C. Kazakhstan | FBA | -18.4 | 15.7 | Homo sapiens | Lightfoot et al 2014 |
| KZF48 | Aschisu | C. Kazakhstan | LBA | -18.6 | 7.9 | Herbivore | Lightfoot et al 2014 |
| KZF49 | Aschisu | C. Kazakhstan | LBA | -18.4 | 8.0 | Herbivore | Lightfoot et al 2014 |
| KZF50 | Aschisu | C. Kazakhstan | LBA | -18.2 | 12.7 | Herbivore | Lightfoot et al 2014 |
| KZF51 | Aschisu | C. Kazakhstan | LBA | -19.8 | 6.2 | Herbivore | Lightfoot et al 2014 |
| KZF52 | Aschisu | C. Kazakhstan | LBA | -20.0 | 6.7 | Herbivore | Lightfoot et al 2014 |
| KZF46 | Tashik | C. Kazakhstan | LBA | -18.6 | 13.3 | Herbivore | Lightfoot et al 2014 |
| KZF47 | Tashik | C. Kazakhstan | LBA | -18.6 | 13.2 | Herbivore | Lightfoot et al 2014 |
| KZF54 | Tashik | C. Kazakhstan | LBA | -19.0 | 7.0 | Herbivore | Lightfoot et al 2014 |
| KZF55 | Tashik | C. Kazakhstan | LBA | -18.9 | 7.6 | Herbivore | Lightfoot et al 2014 |
| KZF56 | Tashik | C. Kazakhstan | LBA | -17.9 | 8.5 | Herbivore | Lightfoot et al 2014 |
| KZF57 | Tashik | C. Kazakhstan | LBA | -20.3 | 5.6 | Herbivore | Lightfoot et al 2014 |

| | | | | | | | |
|---|---|---|---|---|---|---|---|
| KZF58 | Tashik | C. Kazakhstan | LBA | -20.2 | 5.7 | Herbivore | Lightfoot et al 2014 |
| KZF60 | Tashik | C. Kazakhstan | LBA | -20.1 | 6.0 | Herbivore | Lightfoot et al 2014 |
| KZF61 | Tashik | C. Kazakhstan | LBA | -19.0 | 7.7 | Herbivore | Lightfoot et al 2014 |
| KZF62 | Tashik | C. Kazakhstan | LBA | -18.7 | 7.9 | Herbivore | Lightfoot et al 2014 |
| KZF63 | Tashik | C. Kazakhstan | LBA | -19.2 | 7.4 | Herbivore | Lightfoot et al 2014 |
| KZF64 | Tashik | C. Kazakhstan | LBA | -19.0 | 7.6 | Herbivore | Lightfoot et al 2014 |
| KZF65 | Tashik | C. Kazakhstan | LBA | -19.1 | 7.4 | Herbivore | Lightfoot et al 2014 |
| KZF66 | Tashik | C. Kazakhstan | LBA | -19.1 | 7.2 | Herbivore | Lightfoot et al 2014 |
| KZF67 | Tashik | C. Kazakhstan | LBA | -19.8 | 7.6 | Herbivore | Lightfoot et al 2014 |
| KZF68 | Tashik | C. Kazakhstan | LBA | -19.4 | 8.3 | Herbivore | Lightfoot et al 2014 |
| KZF70 | Tashik | C. Kazakhstan | LBA | -19.4 | 7.5 | Herbivore | Lightfoot et al 2014 |
| KZF71 | Tashik | C. Kazakhstan | LBA | -18.7 | 8.0 | Herbivore | Lightfoot et al 2014 |
| KZF02 | Tegiszhol | C. Kazakhstan | LBA | -19.5 | 7.8 | Herbivore | Lightfoot et al 2014 |
| KZF03 | Tegiszhol | C. Kazakhstan | LBA | -18.9 | 7.6 | Herbivore | Lightfoot et al 2014 |
| KZF04 | Tegiszhol | C. Kazakhstan | LBA | -19.1 | 7.9 | Herbivore | Lightfoot et al 2014 |
| KZF05 | Temirkash | C. Kazakhstan | LBA | -18.8 | 8.7 | Herbivore | Lightfoot et al 2014 |
| KZF06 | Temirkash | C. Kazakhstan | LBA | -20.4 | 4.8 | Herbivore | Lightfoot et al 2014 |
| KZF06B | Temirkash | C. Kazakhstan | LBA | -19.2 | 7.7 | Herbivore | Lightfoot et al 2014 |
| KZF07 | Temirkash | C. Kazakhstan | LBA | -18.6 | 8.6 | Herbivore | Lightfoot et al 2014 |
| KZF09 | Temirkash | C. Kazakhstan | LBA | -20.4 | 5.9 | Herbivore | Lightfoot et al 2014 |
| KZF10 | Temirkash | C. Kazakhstan | LBA | -18.6 | 7.8 | Herbivore | Lightfoot et al 2014 |
| KZF11 | Temirkash | C. Kazakhstan | LBA | -18.9 | 8.8 | Herbivore | Lightfoot et al 2014 |
| KZF13 | Temirkash | C. Kazakhstan | LBA | -19.7 | 6.2 | Herbivore | Lightfoot et al 2014 |
| KZF14 | Temirkash | C. Kazakhstan | LBA | -19.3 | 8.2 | Herbivore | Lightfoot et al 2014 |
| KZF14B | Temirkash | C. Kazakhstan | LBA | -18.8 | 6.9 | Herbivore | Lightfoot et al 2014 |
| KZF15 | Temirkash | C. Kazakhstan | LBA | -19.6 | 7.6 | Herbivore | Lightfoot et al 2014 |
| KZF16 | Temirkash | C. Kazakhstan | LBA | -19.1 | 6.3 | Herbivore | Lightfoot et al 2014 |

| | | | | | | | |
|---|---|---|---|---|---|---|---|
| KZF17 | Temirkash | C. Kazakhstan | LBA | -18.8 | 6.3 | Herbivore | Lightfoot et al 2014 |
| KZF18 | Temirkash | C. Kazakhstan | LBA | -19.4 | 8.3 | Herbivore | Lightfoot et al 2014 |
| KZF19 | Temirkash | C. Kazakhstan | LBA | -18.4 | 7.5 | Herbivore | Lightfoot et al 2014 |
| KZF20 | Temirkash | C. Kazakhstan | LBA | -18.8 | 7.1 | Herbivore | Lightfoot et al 2014 |
| KZF21 | Temirkash | C. Kazakhstan | LBA | -20.6 | 5.7 | Herbivore | Lightfoot et al 2014 |
| KZF22 | Temirkash | C. Kazakhstan | LBA | -19.4 | 6.9 | Herbivore | Lightfoot et al 2014 |
| KZF23 | Temirkash | C. Kazakhstan | LBA | -18.8 | 6.1 | Herbivore | Lightfoot et al 2014 |
| KZF24 | Temirkash | C. Kazakhstan | LBA | -20.3 | 6.1 | Herbivore | Lightfoot et al 2014 |
| KZF25 | Temirkash | C. Kazakhstan | LBA | -20.2 | 5.8 | Herbivore | Lightfoot et al 2014 |
| KZF26 | Temirkash | C. Kazakhstan | LBA | -19.7 | 8.7 | Herbivore | Lightfoot et al 2014 |
| KZF27 | Temirkash | C. Kazakhstan | LBA | -19.6 | 8.4 | Herbivore | Lightfoot et al 2014 |
| KZF28 | Temirkash | C. Kazakhstan | LBA | -18.8 | 7.7 | Herbivore | Lightfoot et al 2014 |
| KZF29 | Temirkash | C. Kazakhstan | LBA | -18.7 | 8.2 | Herbivore | Lightfoot et al 2014 |
| KZF30 | Temirkash | C. Kazakhstan | LBA | -20.6 | 6.3 | Herbivore | Lightfoot et al 2014 |
| KZF31 | Temirkash | C. Kazakhstan | LBA | -18.8 | 7.7 | Herbivore | Lightfoot et al 2014 |
| KZF32 | Temirkash | C. Kazakhstan | LBA | -19.7 | 7.1 | Herbivore | Lightfoot et al 2014 |
| KZF33 | Temirkash | C. Kazakhstan | LBA | -18.3 | 7.7 | Herbivore | Lightfoot et al 2014 |
| KZF34 | Temirkash | C. Kazakhstan | LBA | -19.1 | 7.3 | Herbivore | Lightfoot et al 2014 |
| KZF35 | Temirkash | C. Kazakhstan | LBA | -19.0 | 8.8 | Herbivore | Lightfoot et al 2014 |
| KZF37 | Temirkash | C. Kazakhstan | LBA | -18.4 | 6.7 | Herbivore | Lightfoot et al 2014 |
| KZF38 | Temirkash | C. Kazakhstan | LBA | -18.7 | 5.3 | Herbivore | Lightfoot et al 2014 |
| KZF39 | Temirkash | C. Kazakhstan | LBA | -19.1 | 6.0 | Herbivore | Lightfoot et al 2014 |
| KZF40 | Temirkash | C. Kazakhstan | LBA | -20.6 | 6.0 | Herbivore | Lightfoot et al 2014 |
| KZF41 | Temirkash | C. Kazakhstan | LBA | -19.1 | 7.7 | Herbivore | Lightfoot et al 2014 |
| KZF43B | Temirkash | C. Kazakhstan | LBA | -19.3 | 7.3 | Herbivore | Lightfoot et al 2014 |
| KZF43 | Temirkash | C. Kazakhstan | LBA | -19.5 | 5.5 | Herbivore | Lightfoot et al 2014 |
| KZF44 | Temirkash | C. Kazakhstan | LBA | -17.9 | 7.1 | Herbivore | Lightfoot et al 2014 |

| | | | | | | | |
|---|---|---|---|---|---|---|---|
| Al_01 | Itkul (Bolshoi Mys) | S.W. Siberia | NL/CL | -19.7 | 13.4 | Homo sapiens | Motuzaite Matuzeviciute et al. 2016 |
| Al_2A | Itkul (Bolshoi Mys) | S.W. Siberia | NL/CL | -21.0 | 12.9 | Homo sapiens | Motuzaite Matuzeviciute et al. 2016 |
| Al_02B | Itkul (Bolshoi Mys) | S.W. Siberia | NL/CL | -21.1 | 12.6 | Homo sapiens | Motuzaite Matuzeviciute et al. 2016 |
| Al_03 | Itkul (Bolshoi Mys) | S.W. Siberia | NL/CL | -21.7 | 14.3 | Homo sapiens | Motuzaite Matuzeviciute et al. 2016 |
| Al_04 | Itkul (Bolshoi Mys) | S.W. Siberia | NL/CL | -19.9 | 12.6 | Homo sapiens | Motuzaite Matuzeviciute et al. 2016 |
| Al_05 | Itkul (Bolshoi Mys) | S.W. Siberia | NL/CL | -21.3 | 14.6 | Homo sapiens | Motuzaite Matuzeviciute et al. 2016 |
| Al_07 | Itkul (Bolshoi Mys) | S.W. Siberia | NL/CL | -21.1 | 13.3 | Homo sapiens | Motuzaite Matuzeviciute et al. 2016 |
| Al_08 | Itkul (Bolshoi Mys) | S.W. Siberia | NL/CL | -21.0 | 13.4 | Homo sapiens | Motuzaite Matuzeviciute et al. 2016 |
| Al_09 | Itkul (Bolshoi Mys) | S.W. Siberia | NL/CL | -22.1 | 15.6 | Homo sapiens | Motuzaite Matuzeviciute et al. 2016 |
| Al_13 | Ust-Isha | S.W. Siberia | NL/CL | -21.6 | 15.9 | Homo sapiens | Motuzaite Matuzeviciute et al. 2016 |
| Al_15 | Ust-Isha | S.W. Siberia | NL/CL | -21.7 | 15.5 | Homo sapiens | Motuzaite Matuzeviciute et al. 2016 |
| Al_16 | Ust-Isha | S.W. Siberia | NL/CL | -21.4 | 15.5 | Homo sapiens | Motuzaite Matuzeviciute et al. 2016 |
| Al_31 | Firsovo-XI | S.W. Siberia | NL/CL | -22.8 | 13.3 | Homo sapiens | Motuzaite Matuzeviciute et al. 2016 |
| Al_32 | Firsovo-XI | S.W. Siberia | NL/CL | -22.6 | 12.5 | Homo sapiens | Motuzaite Matuzeviciute et al. 2016 |
| Al_33 | Firsovo-XI | S.W. Siberia | NL/CL | -22.9 | 13.5 | Homo sapiens | Motuzaite Matuzeviciute et al. 2016 |
| Al_34 | Firsovo-XI | S.W. Siberia | NL/CL | -23.4 | 14.1 | Homo sapiens | Motuzaite Matuzeviciute et al. 2016 |
| Al_35 | Firsovo-XI | S.W. Siberia | NL/CL | -22.7 | 13.2 | Homo sapiens | Motuzaite Matuzeviciute et al. 2016 |
| Al_36 | Firsovo-XI | S.W. Siberia | NL/CL | -22.8 | 13.4 | Homo sapiens | Motuzaite Matuzeviciute et al. 2016 |
| Al_37 | Firsovo-XI | S.W. Siberia | NL/CL | -22.6 | 13.0 | Homo sapiens | Motuzaite Matuzeviciute et al. 2016 |
| Al_38 | Firsovo-XI | S.W. Siberia | NL/CL | -23.3 | 14.2 | Homo sapiens | Motuzaite Matuzeviciute et al. 2016 |
| Al_39 | Firsovo-XI | S.W. Siberia | NL/CL | -22.5 | 12.8 | Homo sapiens | Motuzaite Matuzeviciute et al. 2016 |
| Al_50 | Solonts-5 | S.W. Siberia | NL/CL | -19.9 | 13.7 | Homo sapiens | Motuzaite Matuzeviciute et al. 2016 |
| Al_51 | Solonts-5 | S.W. Siberia | NL/CL | -18.6 | 12.5 | Homo sapiens | Motuzaite Matuzeviciute et al. 2016 |
| Al_52 | Solonts-5 | S.W. Siberia | NL/CL | -20.8 | 15.3 | Homo sapiens | Motuzaite Matuzeviciute et al. 2016 |
| Al_53 | Solonts-5 | S.W. Siberia | NL/CL | -21.6 | 14.0 | Homo sapiens | Motuzaite Matuzeviciute et al. 2016 |
| Al_54 | Solonts-5 | S.W. Siberia | NL/CL | -20.7 | 13.4 | Homo sapiens | Motuzaite Matuzeviciute et al. 2016 |
| Al_55 | Solonts-5 | S.W. Siberia | NL/CL | -20.6 | 12.9 | Homo sapiens | Motuzaite Matuzeviciute et al. 2016 |

| | | | | | | | |
|---|---|---|---|---|---|---|---|
| Al_17 | Tuzovskiye Bugry-I | S.W. Siberia | NL/CL | -21.8 | 14.3 | Homo sapiens | Motuzaite Matuzeviciute et al. 2016 |
| Al_18 | Tuzovskiye Bugry-I | S.W. Siberia | NL/CL | -22.3 | 13.5 | Homo sapiens | Motuzaite Matuzeviciute et al. 2016 |
| Al_19 | Tuzovskiye Bugry-I | S.W. Siberia | NL/CL | -22.3 | 13.7 | Homo sapiens | Motuzaite Matuzeviciute et al. 2016 |
| Al_20 | Tuzovskiye Bugry-I | S.W. Siberia | NL/CL | -22.4 | 13.7 | Homo sapiens | Motuzaite Matuzeviciute et al. 2016 |
| Al_21 | Tuzovskiye Bugry-I | S.W. Siberia | NL/CL | -22.5 | 14.0 | Homo sapiens | Motuzaite Matuzeviciute et al. 2016 |
| Al_22 | Tuzovskiye Bugry-I | S.W. Siberia | NL/CL | -22.3 | 13.8 | Homo sapiens | Motuzaite Matuzeviciute et al. 2016 |
| Al_23 | Tuzovskiye Bugry-I | S.W. Siberia | NL/CL | -21.6 | 14.2 | Homo sapiens | Motuzaite Matuzeviciute et al. 2016 |
| Al_24 | Tuzovskiye Bugry-I | S.W. Siberia | NL/CL | -22.0 | 13.6 | Homo sapiens | Motuzaite Matuzeviciute et al. 2016 |
| Al_25 | Tuzovskiye Bugry-I | S.W. Siberia | NL/CL | -20.9 | 14.6 | Homo sapiens | Motuzaite Matuzeviciute et al. 2016 |
| Al_26 | Tuzovskiye Bugry-I | S.W. Siberia | NL/CL | -22.5 | 13.6 | Homo sapiens | Motuzaite Matuzeviciute et al. 2016 |
| Al_27 | Tuzovskiye Bugry-I | S.W. Siberia | NL/CL | -22.4 | 13.4 | Homo sapiens | Motuzaite Matuzeviciute et al. 2016 |
| Al_28 | Tuzovskiye Bugry-I | S.W. Siberia | NL/CL | -23.4 | 14.5 | Homo sapiens | Motuzaite Matuzeviciute et al. 2016 |
| Al_29 | Tuzovskiye Bugry-I | S.W. Siberia | NL/CL | -22.7 | 13.8 | Homo sapiens | Motuzaite Matuzeviciute et al. 2016 |
| Al_45 | Teleutsky Vzvoz-I | S.W. Siberia | EBA | -19.4 | 13.1 | Homo sapiens | Motuzaite Matuzeviciute et al. 2016 |
| Al_46 | Teleutsky Vzvoz-I | S.W. Siberia | EBA | -20.1 | 12.6 | Homo sapiens | Motuzaite Matuzeviciute et al. 2016 |
| Al_47 | Teleutsky Vzvoz-I | S.W. Siberia | EBA | -21.0 | 13.2 | Homo sapiens | Motuzaite Matuzeviciute et al. 2016 |
| Al_49 | Teleutsky Vzvoz-I | S.W. Siberia | EBA | -18.3 | 12.4 | Homo sapiens | Motuzaite Matuzeviciute et al. 2016 |
| Al_40 | Firsovo- XIV | S.W. Siberia | MBA | -19.9 | 10.6 | Homo sapiens | Motuzaite Matuzeviciute et al. 2016 |
| Al_41 | Firsovo- XIV | S.W. Siberia | MBA | -19.8 | 11.4 | Homo sapiens | Motuzaite Matuzeviciute et al. 2016 |
| Al_42 | Firsovo- XIV | S.W. Siberia | MBA | -19.4 | 10.9 | Homo sapiens | Motuzaite Matuzeviciute et al. 2016 |
| Al_43 | Firsovo- XIV | S.W. Siberia | MBA | -19.1 | 11.3 | Homo sapiens | Motuzaite Matuzeviciute et al. 2016 |
| Al_44 | Firsovo- XIV | S.W. Siberia | MBA | -19.3 | 10.5 | Homo sapiens | Motuzaite Matuzeviciute et al. 2016 |
| CHA 16 | Chicha | S.W. Siberia | LBA/EIA | -20.2 | 14.4 | Homo sapiens | Privat et al. 2005 |
| CHA 37 | Chicha | S.W. Siberia | LBA/EIA | -20.0 | 13.8 | Homo sapiens | Privat et al. 2005 |
| CHA 14 | Chicha | S.W. Siberia | LBA/EIA | -19.9 | 15.9 | Homo sapiens | Privat et al. 2005 |
| CHA 13 | Chicha | S.W. Siberia | LBA/EIA | -19.8 | 15.5 | Homo sapiens | Privat et al. 2005 |
| CHA 1 | Chicha | S.W. Siberia | LBA/EIA | -19.8 | 12.5 | Homo sapiens | Privat et al. 2005 |

| | | | | | | | |
|---|---|---|---|---|---|---|---|
| CHA 33 | Chicha | S.W. Siberia | LBA/EIA | -19.8 | 14.9 | Homo sapiens | Privat et al. 2005 |
| CHA 32 | Chicha | S.W. Siberia | LBA/EIA | -19.3 | 15.1 | Homo sapiens | Privat et al. 2005 |
| CHA 15 | Chicha | S.W. Siberia | LBA/EIA | -19.2 | 15.0 | Homo sapiens | Privat et al. 2005 |
| CHA 35 | Chicha | S.W. Siberia | LBA/EIA | -19.1 | 14.8 | Homo sapiens | Privat et al. 2005 |
| CHA 34 | Chicha | S.W. Siberia | LBA/EIA | -18.6 | 14.6 | Homo sapiens | Privat et al. 2005 |
| CHA 36 | Chicha | S.W. Siberia | LBA/EIA | -18.6 | 13.3 | Homo sapiens | Privat et al. 2005 |
| CHA 11 | Chicha | S.W. Siberia | LBA/EIA | -20.2 | 6.2 | Equus caballus | Privat et al. 2005 |
| CHA 3 | Chicha | S.W. Siberia | LBA/EIA | -20.3 | 4.4 | Equus caballus | Privat et al. 2005 |
| CHA 38 | Chicha | S.W. Siberia | LBA/EIA | -20.3 | 4.6 | Equus caballus | Privat et al. 2005 |
| CHA 39 | Chicha | S.W. Siberia | LBA/EIA | -20.3 | 5.0 | Equus caballus | Privat et al. 2005 |
| CHA 40 | Chicha | S.W. Siberia | LBA/EIA | -20.9 | 4.8 | Equus caballus | Privat et al. 2005 |
| CHA 42 | Chicha | S.W. Siberia | LBA/EIA | -19.6 | 6.6 | Equus caballus | Privat et al. 2005 |
| CHA 5 | Chicha | S.W. Siberia | LBA/EIA | -19.4 | 5.7 | Ovis aries | Privat et al. 2005 |
| CHA 10 | Chicha | S.W. Siberia | LBA/EIA | -20.1 | 7.3 | Bos taurus | Privat et al. 2005 |
| CHA 12 | Chicha | S.W. Siberia | LBA/EIA | -19.8 | 9.2 | Bos taurus | Privat et al. 2005 |
| CHA 4 | Chicha | S.W. Siberia | LBA/EIA | -20.3 | 4.5 | Bos taurus | Privat et al. 2005 |
| CHA 43 | Chicha | S.W. Siberia | LBA/EIA | -19.0 | 7.0 | Bos taurus | Privat et al. 2005 |
| SS1F | TavdinskyGrotto | S.W. Siberia | NL/CL | -19.9 | 4.9 | Big mammal (probably equus) | Motuzaite Matuzeviciute et al. 2016 |
| SS3F | TavdinskyGrotto | S.W. Siberia | NL/CL | -20.2 | 5.7 | Big mammal/horse | Motuzaite Matuzeviciute et al. 2016 |
| SS5F | TavdinskyGrotto | S.W. Siberia | NL/CL | -18.1 | 4.4 | Equus | Motuzaite Matuzeviciute et al. 2016 |
| SS7F | TavdinskyGrotto | S.W. Siberia | NL/CL | -21.0 | 5.8 | Big mammal | Motuzaite Matuzeviciute et al. 2016 |
| SS8F | TavdinskyGrotto | S.W. Siberia | NL/CL | -19.3 | 6.9 | Big mammal | Motuzaite Matuzeviciute et al. 2016 |
| AlF_01 | TeleutskyVzvoz1 | S.W. Siberia | EBA | -19.0 | 6.0 | Ovicaprid | Motuzaite Matuzeviciute et al. 2016 |
| AlF_02 | TeleutskyVzvoz1 | S.W. Siberia | EBA | -19.5 | 6.1 | Bovine | Motuzaite Matuzeviciute et al. 2016 |
| AlF_03 | TeleutskyVzvoz1 | S.W. Siberia | EBA | -18.4 | 6.0 | Bovine | Motuzaite Matuzeviciute et al. 2016 |
| AlF_04 | TeleutskyVzvoz1 | S.W. Siberia | EBA | -19.6 | 6.9 | Bovine | Motuzaite Matuzeviciute et al. 2016 |
| AlF_05 | TeleutskyVzvoz1 | S.W. Siberia | EBA | -19.7 | 6.9 | Ovicaprid | Motuzaite Matuzeviciute et al. 2016 |

| | | | | | | | |
|---|---|---|---|---|---|---|---|
| AlF_06 | TeleutskyVzvoz1 | S.W. Siberia | EBA | -19.3 | 6.5 | Ovicaprid | Motuzaite Matuzeviciute et al. 2016 |
| AlF_07 | Kolyvanskoye1 | S.W. Siberia | EBA | -20.7 | 3.3 | Bovine | Motuzaite Matuzeviciute et al. 2016 |
| AlF_08 | Kolyvanskoye1 | S.W. Siberia | EBA | -19.1 | 6.4 | Bovine | Motuzaite Matuzeviciute et al. 2016 |
| AlF_09 | Kolyvanskoye1 | S.W. Siberia | EBA | -17.7 | 5.7 | Ovicaprid | Motuzaite Matuzeviciute et al. 2016 |
| AlF_10 | BerezovayaLuka | S.W. Siberia | EBA | -19.0 | 5.2 | Ovicaprid | Motuzaite Matuzeviciute et al. 2016 |
| AlF_11 | BerezovayaLuka | S.W. Siberia | EBA | -19.6 | 6.9 | Bovine | Motuzaite Matuzeviciute et al. 2016 |
| AlF_12 | BerezovayaLuka | S.W. Siberia | EBA | -18.5 | 8.1 | Bovine | Motuzaite Matuzeviciute et al. 2016 |

**Supplemental Figures and Tables**

Figure S1 Carbon ($\delta^{13}C$) and nitrogen ($\delta^{13}N$) isotopic spacing of human bone collagen relative to livestock bone collagen by region.

Figure S2 Isotopic niches of humans by region. Constructed using SIBER (Stable Isotope Bayesian Ellipses in R), R program (2016).

Figure S3 Emergence and intensity of millet and wheat/barley consumption by humans across the Eurasian steppe over time: a) 5000-2600 cal BC, b) 2600-1600 cal BC, and c) 1600-1500 cal BC. Basemap constructed in ArcGIS 10 with hillshade generated using http://www.naturalearthdata.com (freely available). Regions generated in Adobe Illustrator CS6.

Figure S4 Emergence and intensity of millet and wheat/barley consumption by humans across the Eurasian steppe over time: a) 1500-1300 cal BC, b) 1300-1100 cal BC, and c) 1100-1000 cal BC. Basemap constructed in ArcGIS 10 with hillshade generated using http://www.naturalearthdata.com (freely available). Regions generated in Adobe Illustrator CS6.

Figure S5 Emergence and intensity of millet and wheat/barley consumption by humans across the Eurasian steppe over time: a) 1000-500 cal BC, b) 500-200 cal BC, and c) 200 BC – AD 400. Basemap constructed in ArcGIS 10 with hillshade generated using http://www.naturalearthdata.com (freely available). Regions generated in Adobe Illustrator CS6.

Table S1 Site information by region

Table S2 T-tests of regression line slopes (humans compared to herbivores)

Table S3 Tukey's test of significance, comparing human populations in different time slices

Table S4 Comparison of carbon and nitrogen isotope values (minimum, maximum, mean, and standard deviation)

Table S5 Original carbon and nitrogen stable isotope data with references
